# Patterns of care of brain tumor-related epilepsy. A cohort study done in Italian Epilepsy Center

**DOI:** 10.1371/journal.pone.0180470

**Published:** 2017-07-17

**Authors:** Marta Maschio, Ettore Beghi, Marina M. L. Casazza, Gabriella Colicchio, Cinzia Costa, Paola Banfi, Stefano Quadri, Paolo Aloisi, Anna Teresa Giallonardo, Carla Buttinelli, Giada Pauletto, Salvatore Striano, Andrea Salmaggi, Riccardo Terenzi, Ornella Daniele, Giovanni Crichiutti, Francesco Paladin, Rosario Rossi, Giulia Prato, Federico Vigevano, Roberto De Simone, Federica Ricci, Marina Saladini, Fabrizio Monti, Susanna Casellato, Tiziano Zanoni, Diana Giannarelli, Giuliano Avanzini, Umberto Aguglia, BTRE Study Group

**Affiliations:** 1 UOSD di Neurologia, Centro per la Cura dell’Epilessia Tumorale, Istituto Nazionale Tumori Regina Elena, Roma, Italia; 2 IRCCS-Istituto di Ricerche Farmacologiche “Mario Negri”, Milano, Italia; 3 Istituto Nazionale Neurologico “Carlo Besta”, Milano, Italia; 4 Neurochirurgia, Università Cattolica Policlinico “A. Gemelli”, Roma, Italia; 5 Clinica Neurologica, Università degli Studi di Perugia, Ospedale SM Misericordia, Perugia, Italia; 6 Divisione di Neurologia, Ospedale di Circolo, Varese, Italia; 7 USC di Neurologia Centro Regionale Epilessia ASST Papa Giovanni XXIII, Bergamo, Italia; 8 UOC di Neurofisiopatogia, Centro per l’Epilessia, L’Aquila, Italia; 9 Centro Epilessia, Università “La Sapienza”, Policlinico Umberto I, Roma, Italia; 10 Dipartimento di Neurologia, Università “La Sapienza”, Ospedale S. Andrea, Roma, Italia; 11 SOC di Neurologia, Azienda Ospedaliera Universitaria di Udine, Udine, Italia; 12 Centro Epilessia, Università degli studi di Napoli “Federico II”, Policlinico Federico II, Napoli, Italia; 13 Dipartimento di Neuroscienze, ASST, Lecco, Italia; 14 UO Neurologia, Ospedale Villa S Pietro, Roma, Italia; 15 Centro per la Diagnosi e Cura dell’Epilessia, UOC Neurologia, Palermo, Italia; 16 Clinica Pediatrica, Servizio Epilessia Infantile, Azienda Ospedaliera Università di Udine, Udine, Italia; 17 UOC Neurologia, Centro Epilessie, Ospedale S Giovanni e Paolo, Venezia, Italia; 18 UO Neurologia, Ospedale S Francesco, Nuoro, Italia; 19 Centro Epilessie, U.O. Neuropsichiatria Infantile, Istituto Gaslini, Genova, Italia; 20 Dipartimento di Neuroscienze, Ospedale Pediatrico Bambin Gesù, Roma, Italia; 21 UOC Neurologia, Ospedale Sant’Eugenio, Roma, Italia; 22 S.C. Neuropsichiatria Infantile, O.I.R.M., A.O. Città della salute e della scienza, Torino, Italia; 23 Clinica Neurologica,Università di Padova, Padova, Italia; 24 Centro per la Diagnosi e Cura delle Epilessie, UOC Neurologia, Trieste, Italia; 25 Centro per la Diagnosi e Cura delle Epilessie dell’ Età Evolutiva, UOC di NPI, AOU, Sassari, Italia; 26 UO Neurologia, Azienda Ospedaliera Integrata-Universitaria, Verona, Italia; 27 Unità di Biostatistica, Istituto Nazionale Tumori Regina Elena, Roma, Italia; 28 Centro Regionale Epilessia, Università Magna Grecia di Catanzaro, Ospedale Riuniti, Reggio Calabria, Italia; University of Modena and Reggio Emilia, ITALY

## Abstract

Epilepsy is the most common comorbidity in patients with brain tumors. Study Aims: To define characteristics of brain tumor-related epilepsy (BTRE) patients and identify patterns of care. Nationwide, multicenter retrospective cohort study. Medical records of BTRE patients seen from 1/1/2010 to 12/31/2011, followed for at least one month were examined. Information included age, sex, tumor type/treatments, epilepsy characteristics, antiepileptic drugs (AEDs). Time to modify first AED due to inefficacy and/or toxicity was assessed with the Kaplan-Meier method and Cox proportional hazard models were used to identify predictors of treatment outcome. Enrolled were 808 patients (447 men, 361 women) from 26 epilepsy centers. Follow-up ranged 1 to 423 months (median 18 months). 732 patients underwent surgery, 483 chemotherapy (CT), 508 radiotherapy. All patients were treated with AEDs. Levetiracetam was the most common drug. 377 patients (46.7%) were still on first drug at end of follow-up, 338 (41.8%) needed treatment modifications (uncontrolled seizures, 229; side effects, 101; poor compliance, 22). Treatment discontinuation for lack of efficacy was associated with younger age, chemotherapy, and center with <20 cases. Treatment discontinuation for side effects was associated with female sex, enzyme-inducing drugs and center with > 20 cases. About one-half of patients with BTRE were on first AED at end of follow-up. Levetiracetam was the most common drug. A non enzyme-inducing AED was followed by a lower risk of drug discontinuation for SE.

## Introduction

Epilepsy represents the most common comorbidity in patients with brain tumors (BT), with an incidence varying from 35 to 70% [1–5]. Patients with brain tumor-related epilepsy (BTRE) require a multidisciplinary approach to provide optimal care because both epilepsy and the underlying tumor need to be addressed.

In Italy, there is a high number of epilepsy centers. However, to date there has been no organized means of collecting data on the number of BTRE patients accessing these centers or the type of care they are receiving. Thus, the Italian League Against Epilepsy (LICE) BTRE Study Group was established in June 2012. Presently, there are 35 centers adhering to the study group.

Aim of this survey was to identify number and baseline characteristics of BTRE patients and patterns of care in epilepsy centers, paying specific attention to 1) pharmacological and non-pharmacological treatments; 2) outcome of treatment of seizures with reasons for discontinuation of assigned drugs.

## Materials and methods

This is a nationwide, multicenter retrospective cohort study. All 35 centers were invited to participate in the study on a voluntary basis. A patient was included in the study if (s)he had a BT (diagnosed by neuroimaging/biopsy/surgery) and one or more seizures in close temporal association with tumor diagnosis. Patients with history of seizures preceding tumor diagnosis and judged by the caring physician to be unrelated to the tumor site were excluded. With reference to the onset of seizures after the diagnosis, we did not use a specific cut-off as we could not exclude the occurrence of seizures even after prolonged periods of time. Each center was required to send anonymized data regarding BTRE patients seen from January 1/2010 to December 31/2011 and followed for at least one month. The information included age, sex, tumor histology and site, surgery, dates of chemotherapy, radiotherapy courses, date of tumor diagnosis, date and type of first seizure, seizure types, date and type of first and subsequent antiepileptic drugs (AEDs), treatment changes, and last follow-up date. The date of modification of the first AED due to lack of efficacy and/or toxicity was a specific endpoint. Each participating center adhered to a standard protocol for patient follow-up in which the use of AEDs was made in accordance with the guidelines of the International League Against Epilepsy (ILAE) [6].

The information was collected through a formatted Excel worksheet developed by an *ad hoc* committee of the LICE BTRE Study Group and agreed upon by all participating centers. Control of quality and completeness of collected data was performed before analysis; centers were requested to answer specific queries in the event that further clarification was necessary.

In order to reduce selection bias, local investigators were asked to screen all patients present in the centers’ archives and enroll all consecutive patients fulfilling the selection criteria.

### Statistical analysis

Qualitative variables were summarized with absolute frequencies and percentages, while means and standard deviations (SD), medians and range were used for quantitative items as appropriate. Quantitative variables, when needed, were dichotomized using median value as cut-off. Time to event analysis (e.g. time to modify first AED for inefficacy and/or toxicity) was performed with the Kaplan-Meier method and differences were evaluated with the log-rank test. Independent predictors of time to treatment change were assessed with Cox proportional hazard models. The risks were expressed as Hazard Ratios (HR) with 95% Confidence Intervals (95% CI). Different models were used according to age (continuous variable; categorical variable, median age or 35 years as cut-off value) and drugs (active principle; enzyme inducers vs. non-inducers). We decided to categorize patients using 35 years as cut-off value taking into account that the middle adulthood age starts at 35 years [7]. Missing values were reported for each item and no substitutions were made. As this was an exploratory study, a calculation of the sample size was not planned. Data were analyzed using the statistical package IMB SPSS Statistics v.21.0. This study was approved by the Ethical Committee of Regina Elena National Cancer Institute (Ethical Committee of the first author) (IRB:CE/90/12).

## Results

Twenty-six centers participated in this study, 13 from Northern Italy, 8 from Central Italy, and 5 from Southern Italy. Eight hundred and eight patients, 447 males (55.3%) and 361 females (44.7%), were enrolled. The number of patients enrolled in each center ranged from 1 to 224; 15 centers contributed with < 20 patients (smaller centers) and 5 with more than 50 patients (larger centers). The mean age at the first visit in the epilepsy center was 50.4 years (SD 17.1) and the median was 52 years.

Follow-up ranged from 1 month to 423 months with a median of 18 months. Two hundred and eighty-eight cases were followed for less than 12 months, 187 for 12–24 months, and 285 for more than 24 months. Follow-up duration was unknown in 40 cases. Among patients who had BT diagnosis after the appearance of seizures, the median time for diagnosis was 4 months. The Karnovsky Performance Status at the first visit (obtained in 765 patients) ranged between 20 and 100, with a mean score of 81.2 (SD 19.1). The general characteristics of the sample are illustrated in Table 1.

**Table 1:** Clinical characteristics and oncological therapies.

Variable	N	%	% without missing values
***Tumor type***			
Astrocytoma II	62	7.7	7.7
Astrocytoma III	67	8.3	8.4
Oligodendroglioma II	51	6.3	6.4
Oligodendroglioma III	34	4.2	4.2
Oligoastrocytoma II	40	5.0	5.0
Oligoastrocytoma III	52	6.4	6.5
Glioblastoma multiforme	268	33.1	33.5
Metastasis	88	10.9	11.0
Other	139	17.2	17.3
Missing	7	0.9	----
***Tumor site***			
Frontal	284	35.1	
Temporal	147	18.2	
Parietal	85	10.5	
Occipital	14	1.7	
Insula	11	1.4	
Multicentric	267	32.9	
***Seizure type***			
Simple partial	255	31.6	33.3
Complex partial	168	20.8	21.9
Simple partial with secondary generalization	110	13.6	14.4
Complex partial with secondary generalization	82	10.1	10.2
Simple and complex partial	19	2.4	2.5
Generalized tonic-clonic	132	16.3	17.2
Missing	42	5.2	----
***Status epilepticus***			
Convulsive	8	1.0	
Non convulsive	24	3.0	
None	776	96.0	
***Seizure timing***			
Before surgery	491	60.8	75.5
After surgery	152	18.8	23.4
Before and after surgery	7	0.9	1.1
Missing	158	19.6	----
***Surgery***			
Biopsy	44	6.0	
Gross total resection (> 90%)	470	64.2	
Partial resection (< 90%)	214	29.2	
None	4	0.5	
***Chemotherapy***			
Temozolomide	388	80.3	
Fotemustine	9	1.1	
Bevacizumab	1	0.2	
PCV	10	2.1	
Gliadel	7	1.4	
Other	50	10.3	
None	18	3.7	
***Radiotherapy***			
Whole brain RT	88	17.3	
IMRT	38	7.5	
Conformational RT	314	61.8	
Stereotaxic	12	2.4	
Radiosurgery	12	2.4	
None	44	8.7	

AED = Antiepileptic drugs; PCV = Procarbazine, CCNU, and Vincristine; RT = Radiotherapy; IMRT = intensity-modulated radiation therapy.

The most frequent tumor was glioblastoma (268 patients, 33.1%), followed by atypical meningioma (139 patients, 17.2%) and metastases (88 patients, 10.9%). Most frequent sites were frontal lobe (284 patients, 35.2%) and temporal lobe (158 patients, 19.6%). Multiple sites were involved in 250 patients (30.9%).

Focal seizures (FS) were the most frequent type (57.6%) with impairment of consciousness in 42.3%. Simple partial seizures (with or without secondary generalization) were the predominating type in patients with frontal (48.4%), parietal (63.1%) and occipital (50.0%) lesions while complex partial seizures prevailed in patients with temporal lesions (46.4%). Simple partial seizures were the commonest type in patients with multisite lesions (57.2%). Secondary generalized seizures occurred in 40.0% of cases and were associated with FS in 23.7%. Status epilepticus occurred in only 32 patients (4.0%) and was non convulsive in 24 (3%). Seizures occurred predominantly before surgery (491 cases, 60.8%).

Seven hundred and 32 patients (90.6%) received surgery and 470 (64.3%) had total tumor resection (Table 1). Among surgical patients, 693 (85.7%) had only one surgical intervention, 37(4.6%) had two, and only 2(0,2%) had three. Four hundred and 83 patients (59.8%) received chemotherapy (CT). Of them, 396 (82%) received only one cycle, 65 had two (13.5%), and 22 (4.5%) three or more cycles. Five hundred and eight patients (62.9%) received radiotherapy. Of these, 483 (59.8%) had only one cycle, 24 (3%) received two, and one (0.1%) received three cycles.

Four hundred and 21 patients used only one AED (52.1%), 241 patients two (29.8%), 82 patients three (10.1%), 39 patients four (4.8%), and 8 patients five (1%). Levetiracetam was the commonest drug (476 patients, 58.9%), followed by oxcarbazepine (176 patients, 21.8%), phenobarbital (168 patients, 20.8%), carbamazepine (152 patients, 18.8%), and valproate (104 patients, 12.9%). In patients in whom the first AED failed, levetiracetam was still the preferred option (178, 32.2%), followed by oxcarbazepine (73, 13.2%) and carbamazepine (53, 9.6%).

A total of 338 patients (41.8%) needed treatment modification, whereas 377 (46.7%) remained on the first drug until the end of follow-up (55 of them still experiencing seizures). Information on treatment changes was not available in 93 (11.5%) cases.

Five hundred sixty four patients (69.8%) were seizure-free at least follow-up.

Distribution of drugs by timing of administration is in Table 2. Carbamazepine, levetiracetam, oxcarbazepine, phenobarbital, phenytoin and valproate were most commonly used as first drug and tended to decrease thereafter. All other drugs tended to be used as a second or subsequent choice. Reasons for change of a drug included uncontrolled seizures (207 patients), side effects (SEs) (79 patients), SEs and uncontrolled seizures (22 patients), and poor compliance (22 patients). At the last follow-up available, 199 patients still had seizures (24.6%) while 564 were seizure-free (69.8%).

**Table 2:** Antiepileptic drugs by timing of administration.

Drug	First		Second		Third		Fourth		Fifth	
	N	%	N	%	N	%	N	%	N	%
Carbamazepine	99	12.2	34	9.2	15	11.6	3	6.4	1	12.5
Lacosamide	0		13	3.5	13	10.1	10	21.3	1	12.5
Levetiracetam	298	36.9	134	36.3	34	26.4	8	17.0	2	25.0
Lamotrigine	5	0.6	6	1.6	4	3.1	3	6.4	1	12.5
Oxcarbazepine	103	12.7	61	16.5	6	4.7	6	12.8	0	
Phenobarbital	140	17.3	22	6.0	6	4.7	0		0	
Pregabalin	2	0.2	4	1.1	2	1.6	1	2.1	1	12.5
Phenytoin	41	5.1	18	4.9	5	3.9	0		0	
Tiagabine	0		4	1.1	4	3.1	1	2.1	0	
Topiramate	16	2.0	19	5.1	17	13.2	5	10.6	1	
Valproate	65	8.0	25	6.8	13	10.1	2	4.3	0	12.5
Zonisamide	0		4	1.1	3	2.3	2	4.3	0	
Other	12	1.5	9	2.4	3	2.3	6	12.8	0	
2 + Drugs	8	0.8	16	4.4	4	3.1	0		1	12.5
Missing	19	2.4								
**Total**	**808**	**100.0**								

Survival analysis was done only in 567 patients from 23 centers.

**Fig 1:**
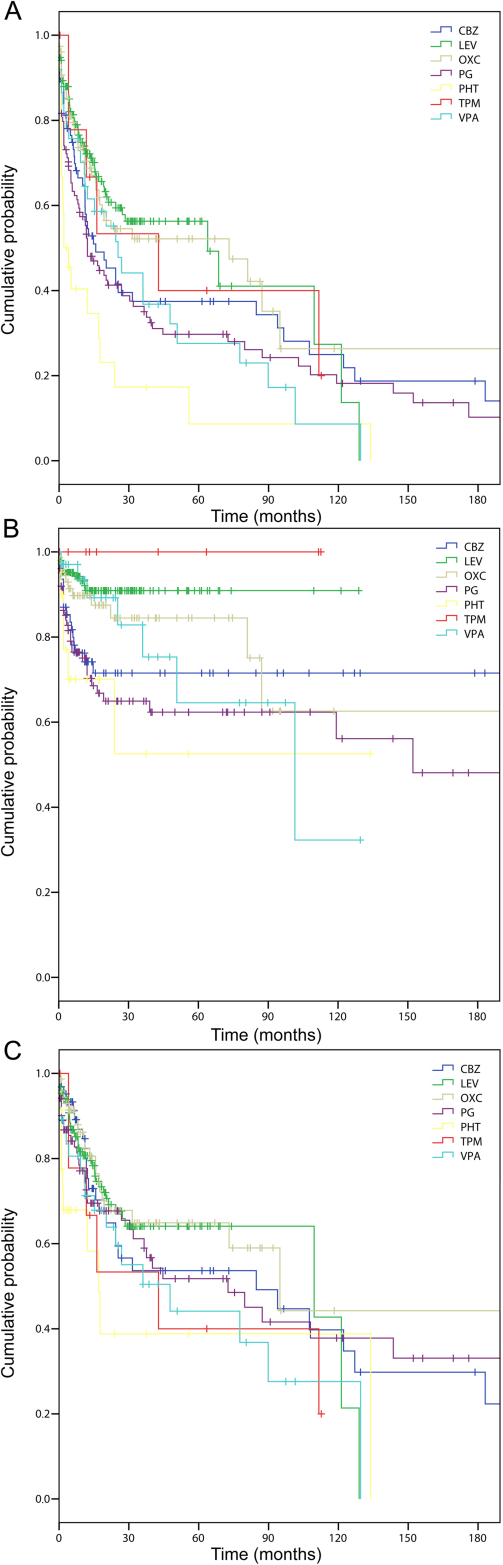
**Time to treatment withdrawal by assigned drug in general (A), for lack of efficacy (B), and for poor tolerability/toxicity (C).** CBZ = Carbamazepine; LEV = Levetiracetam; OXC = Oxcarbazepine; PG = Phenytoin; PHT = Phenobarbital; TPM = Topiramate; VPA = Valproate.

The cumulative time-dependent probability of remaining on first assigned treatment varied significantly across drugs (“Fig 1A”). “Fig 1B” shows time to treatment change of the first AED for lack of efficacy by drug type. Differences were not significant (P = 0.08), but with a tendency towards longer treatment times with levetiracetam and oxcarbazepine. “Fig 1C” shows the time to treatment change for SEs by drug type. Differences were significant (P<0.0001) with a longer retention time for levetiracetam and topiramate and a shorter retention time for phenobarbital and phenytoin.

Factors associated with a lower retention time for lack of efficacy included age 52 years or older and center with more than 20 cases. However, in multivariate analysis age lost significance and use of chemotherapy was associated with a significantly higher probability of drug withdrawal (Table 3). When using age as a continuous variable, age 35 years as cut-off, or drugs with or without induction potential in different models, the only independent predictors of drug discontinuation due to lack of efficacy were young age (HR 0.61; 95% CI 0.42–0.91) and center with less than 20 cases (HR 0.50; 95% CI 0.33–0.76; p = 0.001). When considering age as a continuous variable, HR was 0.98 (95% CI 0.97–0.99).

**Table 3:** Univariate and multivariate Cox models on time to change of first AED for lack of Efficacy.

Variable	Univariate	Multivariate 1	Multivariate 2
Sex (Men vs. Women)	1.08 (0.79–1.46)		
Age (≥52 vs. <52)	0.68 (0.49–0.93)		
Histology			
Low grade gliomas vs. meningiomas	1.44 (0.93–2.21)	1.40 (0.84–2.31)	1.40 (0.84–2.31)
GBM/High grade gliomas vs. meningiomas	1.02 (0.70–1.50)	0.78 (0.47–1.29)	0.78(0.47–1.29)
Metastases vs. meningiomas	0.65 (0.31–1.33)	0.48 (0.22–1.05)	0.48 (0.22–1.05)
Chemotherapy (Yes vs. No)	1.29 (0.93–1.78)	1.66 (1.08–2.54)	1.66 (1.08–2.54)
KPS (>80 vs.≤80)	1.16 (0.84–1.61)		
Interval from first seizure to first AED	0.99 (0.99–1.00)		
Center volume (≥20 patients vs. <20 patients)	0.58 (0.41–0.81)	0.43 (0.28–0.66)	0.43 (0.28–0.66)
First AED			
Levetiracetam vs. carbamazepine	0.90 (0.54–1.49)		
Oxcarbazepine vs. carbamazepine	0.80 (0.45–1.42)		
Phenobarbital vs. carbamazepine	1.07 (0.65–1.76)		
Phenytoin vs. carbamazepine	2.20 (1.08–4.47)		
Topiramate vs. carbamazepine	1.47 (0.60–3.60)		
Valproate vs. carbamazepine	1.41 (0.77–2.56)		
First AED			
Inducers vs. non-inducers	1.26 (0.90–1.77)		
Valproate vs. non-inducers	1.57 (0.94–2.62)		

AED = Antiepileptic drug; GBM = Glioblastoma multiforme; KPS = Karnovsky performance status

Multivariate 1: Model with First AED not grouped; Multivariate 2: model with grouped First AED.

Factors associated with a higher probability of discontinuation of the first AED due to SEs included female sex and use of a drug other than levetiracetam. In multivariate analysis, female sex had borderline significance while risk was increased in centers with more than 20 cases (Table 4). When using age as a continuous variable, age 35 years as cut-off, or drugs with or without induction potential in different models, the only independent predictors of higher probability of drug discontinuation due to SE, were use of enzyme inducing (EI) drugs (HR 3.33; 95% CI 2.02–5.51) and center with more than 20 cases (HR 2.11; 95% CI 1.01–4.40).

**Table 4:** Univariate and multivariate Cox models on time to change of first AED for adverse Effects.

Variable	Univariate	Multivariate 1	Multivariate 2
Sex (Men vs. Women)	0.63 (0.41–0.98)	0.63 (0.40–0.99)	
Age (≥52 vs. <52)	0.84 (0.54–1.30)		
Histology			
Low grade gliomas vs. meningiomas	0.96 (0.51–1.84)		
GBM/High grade gliomas vs. meningiomas	0.80 (0.47–1.37)		
Metastases vs. meningiomas	0.42 (0.47–1.37)		
Chemotherapy (Yes vs. No)	1.56 (1.01–2.41)		
KPS (>80 vs.≤80)	1.41 (0.88–2.26)		
Interval from first seizure to first AED	0.99 (0.99–1.00)		
Center volume (≥20 patients vs. <20 patients)	0.65 (0.79–3.44)	2.81 (1.02–7.72)	2.96 (1.08–8.13)
First AED			
Levetiracetam vs. carbamazepine	0.35 (0.16–0.78)	0.31 (0.14–0.71)	
Oxcarbazepine vs. carbamazepine	0.64 (0.29–1.39)	0.64 (0.29–1.42)	
Phenobarbital vs. carbamazepine	1.45 (0.78–2.68)	1.39 (0.73–2.63)	
Phenytoin vs. carbamazepine	1.74 (0.67–4.49)	1.40 (0.46–4.26)	
Topiramate vs. carbamazepine	Not evaluated	Not evaluated	
Valproate vs. carbamazepine	0.69 (0.34–2.05)	0.77 (0.31–1.93)	
First AED			
Inducers vs. non-inducers	3.02 (1.83–4.98)		3.05 (1.83–5.07)
Valproate vs. non-inducers	1.92 (0.82–4.50)		2.00 (0.85–4.70)

AED = Antiepileptic drug; GBM = Glioblastoma multiforme; KPS = Karnovsky performance status.

Multivariate 1: Model with First AED not grouped; Multivariate 2: model with grouped First AED.

To confirm the lower probability to discontinue non-enzyme inducing (non-EI) drugs, we stratified patients into three different groups: those treated with EI drugs (carbamazepine, phenytoin, phenobarbital), those in therapy with valproate, and those treated with non-EI drugs (levetiracetam, oxcarbazepine, topiramate). In all three groups (Tables 3 and 4) smaller centers and chemotherapy were associated with shorter treatment retention for lack of efficacy. We found, however, an increased risk of drug discontinuation for SEs in women, in patients receiving EI drugs, and in those followed in larger centers.

## Discussion

Our data show that in a retrospective cohort of patients with BTRE seen in a network of epilepsy centers and followed for a median of 18 months, glioblastoma was the most frequent tumor and FS were the predominating seizure type. More than 90% of cases received surgery, more than 60% received radiotherapy and almost 60% received chemotherapy. Almost 50% of cases were on first AED at end of follow-up and 75% were seizure-free. Levetiracetam was the most common drug, followed by oxcarbazepine, phenobarbital and carbamazepine. Seizure relapse was the most frequent explanation for the discontinuation of the first AED, followed by SE. The probability to change the first treatment for lack of efficacy was predicted by younger age, use of chemotherapy and being followed in a center with a small number of BTRE patients. Lack of efficacy was not associated with tumor histology or with type of assigned drug. Change of treatment due to SEs was predicted by management in a center with a large number of patients. Compared to men, women tended to discontinue the first drug more frequently due to SE. Treatment discontinuation for SEs was less frequent with levetiracetam when compared to carbamazepine.

Our data indicate that case volume has a significant impact on quality of care. Patients followed by large centers remain for a longer period of time on the first line AED before changing AED due to lack of efficacy, as compared to patients in small centers. To the contrary, patients followed in large centers spend less time on the first line therapy prior to changing it due to SE. The first situation could be explained by the fact that large centers tend to have experienced physicians who may choose the most effective drug. This is in line with other reports that suggest that the best outcome of patients with head and neck cancers is found in high volume centers [8]. In a comprehensive review of health services literature, hospital or physician volume or specialty has been found to affect outcome of cancer care [9]. Hospital volume has been associated with patterns of care reflecting the most current standards of care in patients with breast cancer [10]. High volume hospitals are associated with better outcomes also for different cancers types [11–17]. For many cancers, being cared for by centers with greater case volumes can prolong the time on a given treatment [8]. The high probability of discontinuation of an AED due to SEs in our patients followed in larger centers may instead reflect the higher attention paid by experienced physicians to the tolerability of the assigned treatment [18].

Chemotherapy was associated with a greater probability of changing the assigned AED for lack of efficacy. Our results appear in contrast with reports that claim a positive role of chemotherapy in seizure control [19,20]. The most important clinical implications of interactions between AEDs and chemotherapy are insufficient control of the tumor or epilepsy, or adverse treatment effects [21–24].

We found more women than men undergoing treatment changes because of unacceptable SE. Men and women are at different risk of experiencing SEs of treatment. Endocrine and reproductive SEs are different in men and women. In the SANAD study, failure of first AED was more common in women than in men [25]. However, this finding remains mostly unexplained because there was no significant difference in the way men and women were dosed, and it is unclear whether there is an important biological difference between sexes in drug response. The effect of sex was not confirmed when we divided the patients into three separate groups: 1) valproate 2) non-EI-AEDs 3) EI-AEDs. Future prospective studies with a particular focus on the role of sex in a patient’s vulnerability to SEs would be necessary.

Levetiracetam was the most common drug used at the start of treatment and in substitution of the first drug. Randomized controlled trials in partial epilepsy and additional studies in gliomas, indicate that levetiracetam is the agent of choice [26]. In a small randomized open-label trial comparing levetiracetam to pregabalin in patients with primary BTs, retention rates were 59% in the levetiracetam group, and 41% in the pregabalin group [27]. In a randomized, open-label trial comparing levetiracetam to phenytoin, the incidence of seizures was significantly less in the levetiracetam group (1.4%) than in the phenytoin group (15.1%) [28]. Among all first-choice AEDs in our patient population, the most favorable prognostic factor for time in therapy, prior to its substitution due to SE, seems to have been treatment with a non-EI-AED, particularly with levetiracetam. These data confirm the more favorable prognosis in patients treated with non-EI rather than EI-AEDs.

Studies on the use of AEDs in BT patients indicate that complete seizure control is not the only challenging goal but that reducing unpleasant SEs produced by AEDs is a serious concern as well [19,29]. SEs are mostly associated with the administration of older AEDs [1,5,27,29]. Limited data regarding AEDs and SEs in patients with BTRE indicate that SEs are less marked when newer AEDs (oxcarbazepine, levetiracetam, topiramate, gabapentin and pregabalin) are administered [22,30,31]. The only study comparing older AEDs with a newer drug (oxcarbazepine) in BTRE patients [31] showed no major differences between the two groups. Concerning safety and tolerability, however, the profiles differed significantly. Older AEDs were associated with more SEs than oxcarbazepine (42.9% vs 11.4%), including events leading to treatment discontinuation.

In our study only about 15% of cases received valproate as first AED or at some point during the course of the disease. Our data did not indicate differences in efficacy between valproate and other AEDs. In the only randomized placebo-controlled trial in adults with newly diagnosed BTs, valproate was not superior to placebo for efficacy [32]. Regarding toxicity as a reason for change, however, there was a significant difference between valproate and newer AEDs, the former being associated to a shorter retention time. Published data highlight the toxicity of valproate in BTRE patients: the drug may increase the hematologic toxicity of chemotherapy, presumably by inhibiting its metabolism, and impairing hemostasis [33–35]. These data are in contrast with recent reports that tend to support the use of valproate in BTRE patients due to its positive effect on survival. In fact, several retrospective studies in seizure patients with glioblastoma treated with chemotherapy provided evidence for a moderately improved survival with the use of valproate, possibly due to inhibition of histone deacetylase [33,36–38]. We think that there is a need to balance the possibility for increased survival with the need to guarantee maximum efficacy and minimum toxicity. Future studies are necessary to address these important issues.

This study has several limitations. First of all, our cohort has been identified in tertiary epilepsy centers and, as such, it does not represent the origin population of BTRE. Our findings are at variance with the results of an Italian regional registry [39] that was based on the participation of different specialists active in the region of interest. BTRE patients followed in neurological or neurosurgical wards are not represented here. As patients seen in epilepsy centers present the most severe epilepsy varieties, outcome of seizures in a population-based sample may be different from that seen in the present cohort. Second, this is a retrospective study. Data have been obtained from medical records where, in the absence of standardized and systematic collection of the required information, variables were available in non-standardized format and were occasionally missing. Third, treatment retention was assessed in an observational context. Physicians’ and patients’ judgment might have had strong influence on the decision to start/stop the assigned treatment. Fourth, this is a multicenter study. Management of the disease varies across centers and this may have a strong impact on study results. All these limitations imply a cautious interpretation of our findings. Based on these considerations, we are now carrying out an observational prospective multicenter study.

BTRE patients are often forced to take many therapies. Adverse effects of AED are common in these patients and can negatively influence their perception of quality of life; this needs to be taken into consideration when choosing an AED as drug efficacy should not be considered the most important selection criterion. This is especially true for fragile patients facing many psychosocial challenges, such as BTRE patients. In absence of well-controlled randomized trials, the decision on which AED provides the best risk-benefit ratio in the individual patient is still based mostly on physician's judgment. In line with several other reports on the efficacy, safety and tolerability of AEDs, retention of the assigned treatment in BTRE patients is fairly high and is mostly affected by the experience of the caring physician and the safety and tolerability profile rather than the efficacy. A non-EI-AED is followed by a lower risk of drug discontinuation for SE. Among non-EI-AEDs, levetiracetam is the preferred drug. Our data are in line with data that indicate patients treated in centers with larger caseloads having a longer retention on a given treatment on account of its higher efficacy.

The results of our study have several implications for clinical practice: 1. Patients with BTRE should be sent to regional reference centers where a multidisciplinary staff is available; 2. Small centers should be in close contact with major centers through a national network; 3. The use of LEV or other non-EI-AED should be preferred; 4. Particular attention should be paid to women for the increased chance of adverse drug reactions.

Based on the limitations of this retrospective survey, emphasis must be given to a prospective cohort study with rigorous eligibility criteria, pre-planned definitions of the outcome variables and of any variable selected as an independent prognostic predictor, and a careful centralized monitoring of data collection.

## Supporting information

S1 Dataset Raw data set. (PDF)

## References

[1] Glantz MJ, Cole BF, Forsyth PA, Recht LD, Wen PY, Chamberlain MC, et al Practice parameter: anticonvulsant prophylaxis in patients with newly diagnosed brain tumors. Neurology. 2000;54: 1886–1893 10822423 10.1212/wnl.54.10.1886

[2] Hildebrand J, Lecaille C, Perennes J, Delattre JY. Epileptic seizures during follow-up of patients treated for primary brain tumors. Neurology. 2005;65: 212–215 doi: 10.1212/01.wnl.0000168903.09277.8f 16043788 10.1212/01.wnl.0000168903.09277.8f

[3] Wen PY, Marks PW. Medical management of patients with brain tumors. Curr Opin Oncol. 2002; 14: 299–307 11981275 10.1097/00001622-200205000-00008

[4] Telfeian AE, Philips MF, Crino PB, Judy KD. Postoperative epilepsy in patients undergoing craniotomy for glioblastoma multiforme. J Exp Clin Cancer Res. 2001; 20: 5–10 11370829

[5] Vecht CJ, Wilms EB. Seizures in low- and high-grade gliomas: current management and future outlook. Expert Rev Anticancer Ther. 2010; 10:663–669 doi: 10.1586/era.10.48 20469999 10.1586/era.10.48

[6] Glauser T, Ben-Menachem E, Bourgeois B, Cnaan A, Guerreiro C, Kälviäinen R, et al Updated ILAE evidence review of antiepileptic drug efficacy and effettiveness as initial monotherapy for epileptic seizures and syndromes. Epilepsia. 2013;54(3): 551–563 doi: 10.1111/epi.12074 23350722 10.1111/epi.12074

[7] Editors Willis Sherry L., Martin Mike. Middle Adulthood: A Lifespan Perspective. California SAGE pubblications, INc; 2005

[8] Corry J, Peters LJ, and Rischin D. Impact of Center Size and Experience on Outcomes in Head and Neck Cancer. J Clin Oncol. 2015;33(2): 138–140 doi: 10.1200/JCO.2014.58.2239 25488964 10.1200/JCO.2014.58.2239

[9] Hillner BE, Smith TJ, Desch CE. Hospital and physician volume or specialization and outcomes in cancer treatment: importance in quality of cancer care. J Clin Oncol. 2000;18(11): 2327–2340 doi: 10.1200/JCO.2000.18.11.2327 10829054 10.1200/JCO.2000.18.11.2327

[10] Kong AL, Pezzin LE, Nattinger AB. Identifying patterns of breast cancer care provided at high-volume hospitals: a classification and regression tree analysis. Breast Cancer Res Treat. 2015;153(3): 689–698 doi: 10.1007/s10549-015-3561-6 26409836 10.1007/s10549-015-3561-6

[11] Joudi FN, Konety BR. The impact of provider volume on outcomes from urological cancer therapy. J Urol 2005;174(2): 432–438 doi: 10.1097/01.ju.0000165340.53381.48 16006859 10.1097/01.ju.0000165340.53381.48

[12] Barocas DA, Mitchell R, Chang SS, Cookson MS. Impact of surgeon and hospital volume on outcomes of radical prostatectomy. Urol Oncol. 2010;28(3): 243–50 doi: 10.1016/j.urolonc.2009.03.001 19395287 10.1016/j.urolonc.2009.03.001

[13] Go RS, Bottner WA, Gertz MA. Making the Case to Study the Volume-Outcome Relationship in Hematologic Cancers. Mayo Clin Proc. 2015;90(10): 1393–1399 doi: 10.1016/j.mayocp.2015.07.003 26298310 10.1016/j.mayocp.2015.07.003

[14] Dikken JL, Dassen AE, Lemmens VE, Putter H, Krijnen P, van der Geest L, et al Effect of hospital volume on postoperative mortality and survival after oesophageal and gastric cancer surgery in the Netherlands between 1989 and 2009. Eur J Cancer. 2012;48(7): 1004–1013 doi: 10.1016/j.ejca.2012.02.064 22456179 10.1016/j.ejca.2012.02.064

[15] Markar SR, Karthikesalingam A, Thrumurthy S, Low DE. Volume-outcome relationship in surgery for esophageal malignancy: systematic review and meta-analysis 2000–2011. J Gastrointest Surg. 2012;16(5): 1055–1063 doi: 10.1007/s11605-011-1731-3 22089950 10.1007/s11605-011-1731-3

[16] Dimick JB, Cowan JA Jr, Upchurch GR Jr, Colletti LM. Hospital volume and surgical outcomes for elderly patients with colorectal cancer in the United States. J Surg Res. 2003;114(1): 50–56 13678698 10.1016/s0022-4804(03)00207-5

[17] Cheung MC, Hamilton K, Sherman R, Byrne MM, Nguyen DM, Franceschi D, et al Impact of teaching facility status and high-volume centers on outcomes for lung cancer resection: an examination of 13,469 surgical patients. Ann Surg Oncol. 2009;16(1): 3–13 doi: 10.1245/s10434-008-0025-9 18600379 10.1245/s10434-008-0025-9

[18] Karpa KD, Hom LL, Huffman P, Lehman EB, Chinchilli VM, Haidet P, et al Medication safety curriculum: enhancing skills and changing behaviors. BMC Med Educ. 2015;29(15): 234–239 10.1186/s12909-015-0521-0 PMC4693404 26711130

[19] Rudà R, Trevisan E, Soffietti R. Epilepsy and brain tumors. Curr Opin Oncol. 2010;22(6): 611–620 doi: 10.1097/CCO.0b013e32833de99d 20706121 10.1097/CCO.0b013e32833de99d

[20] Hu A, Xu Z, Kim RY, Nguyen A, Lee JW, Kesari S. Seizure control: a secondary benefit of chemotherapeutic temozolomide in brain cancer patients. Epilepsy Res. 2011;95(3): 270–272 doi: 10.1016/j.eplepsyres.2011.03.018 21549564 10.1016/j.eplepsyres.2011.03.018

[21] van Breemen MS, Wilms EB, Vecht CJ. Epilepsy in patients with brain tumours: epidemiology, mechanisms, and management. Lancet Neurol. 2007;6(5): 421–430 doi: 10.1016/S1474-4422(07)70103-5 17434097 10.1016/S1474-4422(07)70103-5

[22] Yap KY, Chui WK, Chan A. Drug interactions between chemotherapeutic regimes and antiepileptics. Clin Ther. 2008;30(8): 1385–1407 doi: 10.1016/j.clinthera.2008.08.011 18803983 10.1016/j.clinthera.2008.08.011

[23] Armstrong TS, Grant R, Gilbert MR, Lee JW, Norden AD. Epilepsy in glioma patients: mechanisms, management, and impact of anticonvulsant therapy. Neuro Oncol. 2016; 18(6): 779–789 doi: 10.1093/neuonc/nov269 26527735 10.1093/neuonc/nov269 PMC4864254

[24] Perucca E. Optimizing antiepileptic drug treatment in tumoral epilepsy. Epilepsia. 2013;54(9): 97–104 24328881 10.1111/epi.12452

[25] Bonnett LJ, Tudur Smith C, Donegan S, Marson AG. Treatment outcome after failure of a first antiepileptic drug. Neurology. 2014;83: 552–560 doi: 10.1212/WNL.0000000000000673 24994842 10.1212/WNL.0000000000000673 PMC4142004

[26] Yuan Y, Peizhi Z, Maling G, Wu L, Yunhe M, Xiang W, et al The efficacy of levetiracetam for patients with supratentorial brain tumors. J Clin Neurosci. 2015;22(8): 1227–1231 doi: 10.1016/j.jocn.2015.01.025 26051171 10.1016/j.jocn.2015.01.025

[27] Rossetti AO, Jeckelmann S, Novy J, Roth P, Weller M, Stupp R. Levetiracetam and pregabalin for antiepileptic monotherapy in patients with primary brain tumors. A phase II randomized study. Neuro Oncol. 2014;16(4): 584–588 doi: 10.1093/neuonc/not170 24311644 10.1093/neuonc/not170 PMC3956345

[28] Iuchi T, Kuwabara K, Matsumoto M, Kawasaki K, Hasegawa Y, SakaidaT. Levetiracetam versus phenytoin for seizure prophylaxis during and early after craniotomy for brain tumours: a phase II prospective, randomised study. J Neurol Neurosurg Psychiatry. 2015; 86(10):1158–1162 doi: 10.1136/jnnp-2014-308584 25511789 10.1136/jnnp-2014-308584

[29] Maschio M, Sperati F, Dinapoli L, Vidiri A, Fabi A, Pace A, et al Weight of epilepsy in brain tumor patients. J Neurooncol. 2014;118(2): 385–393 doi: 10.1007/s11060-014-1449-7 24789254 10.1007/s11060-014-1449-7

[30] Newton HB, Goldlust SA, Pearl D. Retrospective analysis of the efficacy and tolerability of levetiracetam in brain tumor patients. J Neurooncol 2006, 78:99–102 doi: 10.1007/s11060-005-9070-4 16541329 10.1007/s11060-005-9070-4

[31] Maschio M, Dinapoli L, Vidiri A, Pace A, Fabi A, Pompili A, et al The role side effects play in the choice of antiepileptic therapy in brain tumor-related epilepsy: a comparative study on traditional antiepileptic drugs versus oxcarbazepine. J Exp Clin Cancer Res. 2009;6(28): 60–65 10.1186/1756-9966-28-60 PMC2686682 19419544

[32] Glantz MJ, Cole BF, Friedberg MH, Lathi E, Choy H, Furie K, et al A randomized, blinded, placebo-controlled trial of divalproex sodium prophylaxis in adults with newly diagnosed brain tumors. Neurology. 1996;46(4): 985–991 8780077 10.1212/wnl.46.4.985

[33] Weller M, Stupp R, Wick W. Epilepsy meets cancer: when, why, and what to do about it? Lancet Oncol 2012;13: e375–e382 doi: 10.1016/S1470-2045(12)70266-8 22935237 10.1016/S1470-2045(12)70266-8

[34] Bourg V, Lebrun C, Chichmanian RM, Thomas P, Frenay M. Nitroso-urea-cisplatin-based chemotherapy associated with valproate: increase of haematologic toxicity. Ann Oncol 2001;12: 217–219 11300327 10.1023/a:1008331708395

[35] Oberndorfer S, Piribauer M, Marosi C, Lahrmann H, Hitzenberger P, Grisold W. P450 enzyme inducing and non-enzyme inducing antiepileptics in glioblastoma patients treated with standard chemotherapy. J Neurooncol 2005;72: 255–260 doi: 10.1007/s11060-004-2338-2 15937649 10.1007/s11060-004-2338-2

[36] de Groot M, Reijneveld JC, Aronica E, Heimans JJ. Epilepsy in patients with a brain tumour: focal epilepsy requires focused treatment. Brain. 2012;135(4): 1002–1016 22171351 10.1093/brain/awr310

[37] Guthrie GD, Eljamel S. Impact of particular antiepileptic drugs on the survival of patients with glioblastoma multiforme. J Neurosurg. 2013;118(4): 859–865 doi: 10.3171/2012.10.JNS12169 23176328 10.3171/2012.10.JNS12169

[38] Osuka S, Takano S, Watanabe S, Ishikawa E, Yamamoto T, Matsumura A. Valproic acid inhibits angiogenesis in vitro and glioma angiogenesis in vivo in the brain. Neurol Med Chir (Tokyo). 2012;52(4): 186–193 22522328 10.2176/nmc.52.186

[39] Michelucci R, Pasini E, Meletti S, Fallica E, Rizzi R, Florindo I, et al Epilepsy in primary cerebral tumors: the characteristics of epilepsy at the onset (results from the PERNO study—Project of Emilia Romagna Region on Neuro-Oncology). Epilepsia. 2013;54(7): 86–91 24099060 10.1111/epi.12314

