# Supplementary material for: Patterns of care of brain tumor-related epilepsy. A cohort study done in Italian Epilepsy Center
Source: PLoS One. 2017 Jul 17;12(7):e0180470. doi: 10.1371/journal.pone.0180470 (PMC5513411; doi:10.1371/journal.pone.0180470)
Supplement: S1 Dataset — (PDF) [file pone.0180470.s001.pdf]

VAR00001	CENTER	AGE	SEX	KPS	Histology	Lesion_site	Lesion_site_cod	Surgery1	Surgery1_type
1.00	BERGAMO	69.00	M	70.00	METASTASES	MULTILOBULAR	MULTICENTRIC	NO	
2.00	BERGAMO	56.00	M	50.00	GBM	MULTICENTRIC	MULTICENTRIC	NO	
3.00	BERGAMO	60.00	F	60.00	METASTASES	MULTILOBULAR	MULTICENTRIC	NO	
4.00	BERGAMO	64.00	F	70.00	METASTASES	MULTILOBULAR	MULTICENTRIC	NO	
5.00	BERGAMO	57.00	M	70.00	METASTASES	FRONTAL	FRONTAL	YES	BIOPSY
6.00	BERGAMO	56.00	F	70.00	METASTASES	MULTILOBULAR	MULTICENTRIC	NO	
7.00	BERGAMO	74.00	M	80.00	METASTASES	MULTILOBULAR	MULTICENTRIC	NO	
8.00	BERGAMO	64.00	F	90.00	GBM	FRONTAL	FRONTAL	YES	PARTIAL RESECTION (<90%)
9.00	BERGAMO	68.00	M	70.00	METASTASES	MULTILOBULAR	MULTICENTRIC	NO	
10.00	BERGAMO	41.00	F	90.00	METASTASES	MULTILOBULAR	MULTICENTRIC	NO	
11.00	BERGAMO	54.00	M	100.00	OTHER (ATIPIC MENINGIOMI)	FRONTAL	FRONTAL	YES	BIOPSY
12.00	BERGAMO	13.00	M	90.00	ASTROC III	TEMPORAL	TEMPORAL	YES	BIOPSY
13.00	BERGAMO	47.00	F	90.00	OLIGODENDROGLIOMA III	FRONTAL	FRONTAL	YES	GROSS TOTAL RESECTION (>90
14.00	BERGAMO	28.00	F	100.00	METASTASES	TEMPORAL	TEMPORAL	YES	GROSS TOTAL RESECTION (>90
15.00	BERGAMO	69.00	F	90.00	OTHER (ATIPIC MENINGIOMI)	FRONTAL	FRONTAL	YES	GROSS TOTAL RESECTION (>90
16.00	BERGAMO	52.00	F	100.00	OTHER (ATIPIC MENINGIOMI)	TEMPORAL	TEMPORAL	YES	BIOPSY
17.00	BERGAMO	64.00	F	100.00	OTHER (ATIPIC MENINGIOMI)	FRONTAL	FRONTAL	YES	GROSS TOTAL RESECTION (>90
18.00	BERGAMO	57.00	M	100.00	OTHER (ATIPIC MENINGIOMI)	FRONTAL	FRONTAL	YES	GROSS TOTAL RESECTION (>90
19.00	BERGAMO	39.00	M	90.00	ASTRO II	FRONTAL	FRONTAL	YES	PARTIAL RESECTION (<90%)
20.00	BERGAMO	51.00	M	90.00	OLIGODENDROGLIOMA II	FRONTAL	FRONTAL	YES	GROSS TOTAL RESECTION (>90
21.00	BERGAMO	55.00	M	100.00	ASTRO II	TEMPORAL	TEMPORAL	YES	BIOPSY
22.00	BERGAMO	62.00	M	100.00	OTHER (ATIPIC MENINGIOMI)	FRONTAL	FRONTAL	YES	BIOPSY
23.00	BERGAMO	77.00	F	90.00	OTHER (ATIPIC MENINGIOMI)	TEMPORAL	TEMPORAL	YES	GROSS TOTAL RESECTION (>90
24.00	BERGAMO	63.00	F	100.00	OTHER (ATIPIC MENINGIOMI)	FRONTAL	FRONTAL	YES	GROSS TOTAL RESECTION (>90
25.00	BERGAMO	53.00	M	100.00	OTHER (ATIPIC MENINGIOMI)	FRONTAL	FRONTAL	YES	GROSS TOTAL RESECTION (>90
26.00	BERGAMO	75.00	F	90.00	OTHER (ATIPIC MENINGIOMI)	FRONTAL	FRONTAL	YES	GROSS TOTAL RESECTION (>90
27.00	BERGAMO	73.00	M	80.00	METASTASES	PARIETAL	PARIETAL	YES	GROSS TOTAL RESECTION (>90
28.00	BERGAMO	40.00	F	100.00	ASTRO II	TEMPORAL	TEMPORAL	YES	GROSS TOTAL RESECTION (>90
29.00	BERGAMO	25.00	M	100.00	ASTRO II	FRONTAL	FRONTAL	YES	GROSS TOTAL RESECTION (>90
30.00	BERGAMO	52.00	F	90.00	OTHER (ATIPIC MENINGIOMI)	PARIETAL	PARIETAL	YES	BIOPSY
31.00	BERGAMO	48.00	M	90.00	OLIGOASTROCITOMA II	PARIETAL	PARIETAL	YES	PARTIAL RESECTION (<90%)
32.00	BERGAMO	42.00	M	100.00	OTHER (ATIPIC MENINGIOMI)	FRONTAL	FRONTAL	YES	GROSS TOTAL RESECTION (>90
33.00	BERGAMO	60.00	F	90.00	METASTASES	PARIETAL	PARIETAL	YES	GROSS TOTAL RESECTION (>90
34.00	BERGAMO	49.00	F	100.00	ASTROC III	FRONTAL	FRONTAL	YES	GROSS TOTAL RESECTION (>90
35.00	BERGAMO	58.00	F	90.00	OTHER (ATIPIC MENINGIOMI)	FRONTAL	FRONTAL	YES	GROSS TOTAL RESECTION (>90
36.00	BERGAMO	50.00	M	100.00	OTHER (ATIPIC MENINGIOMI)	FRONTAL	FRONTAL	YES	GROSS TOTAL RESECTION (>90
37.00	BERGAMO	72.00	F	90.00	OTHER (ATIPIC MENINGIOMI)	PARIETAL	PARIETAL	YES	GROSS TOTAL RESECTION (>90
38.00	BERGAMO	72.00	M	90.00	OTHER (ATIPIC MENINGIOMI)	FRONTAL	FRONTAL	YES	BIOPSY
39.00	BERGAMO	25.00	M	90.00	OTHER (ATIPIC MENINGIOMI)	OCCIPITAL	OCCIPITAL	YES	PARTIAL RESECTION (<90%)

40.00	BERGAMO	40.00	M	90.00	ASTROC III	FRONTAL	FRONTAL	YES	PARTIAL RESECTION (<90%)
41.00	BERGAMO	25.00	M	90.00	ASTROC III	FRONTAL	FRONTAL	YES	GROSS TOTAL RESECTION (>90
42.00	UDINE-AZ OSP UNI	12.00	M	100.00	ASTROC III	TEMPORAL	TEMPORAL	YES	GROSS TOTAL RESECTION (>90
43.00	UDINE-AZ OSP UNI	2.00	M	30.00	ASTROC III			YES	PARTIAL RESECTION (<90%)
44.00	UDINE-AZ OSP UNI	15.00	M		ASTRO II	TEMPORAL	TEMPORAL	YES	GROSS TOTAL RESECTION (>90
45.00	UDINE-AZ OSP UNI	21.00	M		ASTRO II	FRONTAL+TEMPORA	FRONTAL+TEMPO	YES	PARTIAL RESECTION (<90%)
46.00	UDINE-AZ OSP UNI	9.00	F		ASTROC III	HYPOTHALAMIC OPT	HYPOTHALAMIC	YES	PARTIAL RESECTION (<90%)
47.00	UDINE-AZ OSP UNI		F	100.00	ASTRO II	PARIETAL+OCCIPITA	PARIETAL+OCCIP	YES	GROSS TOTAL RESECTION (>90
48.00	UDINE-AZ OSP UNI	2.00	M		OLIGODENDROGLIOMA II	TEMPORAL	TEMPORAL	YES	GROSS TOTAL RESECTION (>90
49.00	UDINE-AZ OSP UNI	7.00	M		OLIGOASTROCITOMA II	PARIETAL+OCCIPITA	PARIETAL+OCCIP	YES	PARTIAL RESECTION (<90%)
50.00	UDINE-AZ OSP UNI	4.00	M		ASTRO II	TEMPORAL	TEMPORAL	YES	PARTIAL RESECTION (<90%)
51.00	UDINE-AZ OSP UNI	3.00	M	100.00	ASTRO II	HYPOTHALAMIC OPT	HYPOTHALAMIC	YES	GROSS TOTAL RESECTION (>90
52.00	GENOVA	13.00	F	90.00	OTHER (ATIPIC MENINGIOMI)	TEMPORAL	TEMPORAL	YES	GROSS TOTAL RESECTION (>90
53.00	GENOVA	7.00	F	70.00	OTHER (ATIPIC MENINGIOMI)	BILATERAL	MULTICENTRIC	NO	
54.00	GENOVA	22.00	F	90.00	OTHER (ATIPIC MENINGIOMI)	FRONTAL	FRONTAL	YES	GROSS TOTAL RESECTION (>90
55.00	GENOVA	3.00	M	90.00	OTHER (ATIPIC MENINGIOMI)	BILATERAL	MULTICENTRIC	YES	GROSS TOTAL RESECTION (>90
56.00	GENOVA	13.00	F	80.00	OTHER (ATIPIC MENINGIOMI)	TEMPORAL	TEMPORAL	YES	GROSS TOTAL RESECTION (>90
57.00	GENOVA	13.00	F	90.00	OTHER (ATIPIC MENINGIOMI)	FRONTAL	FRONTAL	YES	GROSS TOTAL RESECTION (>90
58.00	GENOVA	3.00	F	60.00	OTHER (ATIPIC MENINGIOMI)	BILATERAL	MULTICENTRIC	YES	PARTIAL RESECTION (<90%)
59.00	GENOVA	10.00	F	80.00	OTHER (ATIPIC MENINGIOMI)	TEMPORAL	TEMPORAL	YES	PARTIAL RESECTION (<90%)
60.00	L'AQUILA	48.00	M	100.00	OLIGODENDROGLIOMA II	FRONTAL	FRONTAL	YES	GROSS TOTAL RESECTION (>90
61.00	L'AQUILA	38.00	M	90.00	OLIGODENDROGLIOMA II	FRONTAL+TEMPORA	FRONTAL+TEMPO	YES	PARTIAL RESECTION (<90%)
62.00	L'AQUILA	48.00	M	80.00	OLIGOASTROCITOMA III	FRONTAL+TEMPORA	FRONTAL+TEMPO	YES	GROSS TOTAL RESECTION (>90
63.00	L'AQUILA	55.00	F	80.00	GBM	FRONTAL+INSULA	FRONTAL+INSULA	YES	PARTIAL RESECTION (<90%)
64.00	L'AQUILA	52.00	M	60.00	OLIGOASTROCITOMA III	FRONTAL+PARIETAL	FRONTAL+PARIET	YES	PARTIAL RESECTION (<90%)
65.00	L'AQUILA	74.00	M	70.00	GBM	TEMPORAL+PARIETA	TEMPORAL+PARI	YES	PARTIAL RESECTION (<90%)
66.00	L'AQUILA	55.00	F	100.00	OLIGODENDROGLIOMA III	FRONTAL+INSULA	FRONTAL+INSULA	YES	GROSS TOTAL RESECTION (>90
67.00	L'AQUILA	47.00	F	30.00	METASTASES	MULTICENTRIC	MULTICENTRIC	NO	
68.00	L'AQUILA	53.00	M	70.00	GBM	FRONTAL	FRONTAL	YES	PARTIAL RESECTION (<90%)
69.00	L'AQUILA	64.00	M	80.00	METASTASES	FRONTAL+PARIETAL	FRONTAL+PARIET	NO	
70.00	L'AQUILA	73.00	F	40.00	METASTASES	TEMPORAL+PARIETA	TEMPORAL+PARI	NO	
71.00	L'AQUILA	57.00	M	100.00	GBM	FRONTAL	FRONTAL	YES	GROSS TOTAL RESECTION (>90
72.00	L'AQUILA	64.00	F	80.00	GBM	TEMPORAL	TEMPORAL	YES	PARTIAL RESECTION (<90%)
73.00	L'AQUILA	79.00	M	50.00	METASTASES	MULTICENTRIC	MULTICENTRIC	NO	
74.00	L'AQUILA	56.00	F	70.00	METASTASES	TEMPORAL+INSULA	TEMPORAL+INSU	YES	GROSS TOTAL RESECTION (>90
75.00	L'AQUILA	48.00	M	60.00	METASTASES	MULTICENTRIC	MULTICENTRIC	NO	
76.00	L'AQUILA	37.00	M	60.00	ASTROC III+GBM	PARIETAL+OCCIPITA	PARIETAL+OCCIP	YES	GROSS TOTAL RESECTION (>90
77.00	L'AQUILA	66.00	F	90.00	METASTASES	FRONTAL+TEMPORA	FRONTAL+TEMPO	NO	
78.00	L'AQUILA	67.00	F	90.00	OLIGODENDROGLIOMA II	FRONTAL+INSULA	FRONTAL+INSULA	YES	PARTIAL RESECTION (<90%)
79.00	L'AQUILA	59.00	F	80.00	METASTASES	CEREBELLAR	CEREBELLAR	NO	
80.00	L'AQUILA	35.00	F	100.00	OTHER (ATIPIC MENINGIOMI)	CEREBELLAR	CEREBELLAR	YES	GROSS TOTAL RESECTION (>90
81.00	L'AQUILA	52.00	F	70.00	METASTASES	TEMPORAL+PARIETA	TEMPORAL+PARI	YES	PARTIAL RESECTION (<90%)

82.00	L'AQUILA	52.00	F	70.00	METASTASES	MULTICENTRIC	MULTICENTRIC	NO	
83.00	L'AQUILA	70.00	F	70.00	METASTASES	MULTICENTRIC	MULTICENTRIC	NO	
84.00	L'AQUILA	82.00	M	70.00	GBM	FRONTAL+TEMPORA	FRONTAL+TEMP(	YES	GROSS TOTAL RESECTION (>90
85.00	L'AQUILA	39.00	F	100.00	ASTRO II	FRONTAL	FRONTAL	YES	GROSS TOTAL RESECTION (>90
86.00	L'AQUILA	61.00	M	90.00	GBM	TEMPORAL+PARIETA	TEMPORAL+PARI	YES	PARTIAL RESECTION (<90%)
87.00	L'AQUILA	48.00	M	80.00	GBM	PARIETAL+OCCIPITA	PARIETAL+OCCIP	YES	GROSS TOTAL RESECTION (>90
88.00	L'AQUILA	60.00	M	100.00	OLIGODENDROGLIOMA II	FRONTAL	FRONTAL	YES	GROSS TOTAL RESECTION (>90
89.00	L'AQUILA	64.00	F	60.00	METASTASES	TEMPORAL+INSULA	TEMPORAL+INSU	NO	
90.00	L'AQUILA	71.00	M	70.00	METASTASES	TEMPORAL+OCCIPIT	TEMPORAL+OCCI	NO	
91.00	L'AQUILA	33.00	F	100.00	ASTROC III	TEMPORAL	TEMPORAL	YES	PARTIAL RESECTION (<90%)
92.00	L'AQUILA	63.00	M	90.00	GBM	TEMPORAL	TEMPORAL	YES	GROSS TOTAL RESECTION (>90
93.00	L'AQUILA	58.00	M	100.00	OTHER (ATIPIC MENINGIOMI)	PARIETAL	PARIETAL	YES	GROSS TOTAL RESECTION (>90
94.00	L'AQUILA	31.00	M	80.00	ASTROC III	MULTICENTRIC	MULTICENTRIC	YES	PARTIAL RESECTION (<90%)
95.00	L'AQUILA	79.00	F	60.00	GBM			YES	GROSS TOTAL RESECTION (>90
96.00	L'AQUILA	53.00	M	80.00	METASTASES	PARIETAL+CEREBELI	PARIETAL+CEREB	NO	
97.00	NUORO	75.00	M		GBM	TEMPORAL	TEMPORAL	NO	
98.00	NUORO	58.00	F		OLIGODENDROGLIOMA III	TEMPORAL	TEMPORAL	YES	GROSS TOTAL RESECTION (>90
99.00	NUORO	68.00	F		GBM	PARIETAL	PARIETAL	YES	GROSS TOTAL RESECTION (>90
100.00	NUORO	72.00	F	90.00	OTHER (ATIPIC MENINGIOMI)	TEMPORAL	TEMPORAL	YES	GROSS TOTAL RESECTION (>90
101.00	NUORO	51.00	F	60.00	OTHER (ATIPIC MENINGIOMI)	MULTICENTRIC	MULTICENTRIC	YES	GROSS TOTAL RESECTION (>90
102.00	NUORO	48.00	F	100.00	OTHER (ATIPIC MENINGIOMI)	TEMPORAL	TEMPORAL	NO	
103.00	NUORO	55.00	M		ASTRO II	FRONTAL	FRONTAL	YES	PARTIAL RESECTION (<90%)
104.00	NUORO	43.00	F		GBM	TEMPORAL	TEMPORAL	YES	GROSS TOTAL RESECTION (>90
105.00	NUORO	51.00	F		ASTRO II +GBM	FRONTAL	FRONTAL	YES	GROSS TOTAL RESECTION (>90
106.00	PADOVA	44.00	M		GBM	TEMPORAL	TEMPORAL	YES	GROSS TOTAL RESECTION (>90
107.00	PADOVA	44.00	M		GBM	TEMPORAL	TEMPORAL	YES	GROSS TOTAL RESECTION (>90
108.00	PADOVA	44.00	F		GBM	PARIETAL	PARIETAL	YES	GROSS TOTAL RESECTION (>90
109.00	PADOVA	22.00	M		ASTRO II	TEMPORAL	TEMPORAL	YES	GROSS TOTAL RESECTION (>90
110.00	PALERMO	51.00	M	70.00	OTHER (ATIPIC MENINGIOMI)	FRONTAL	FRONTAL	YES	GROSS TOTAL RESECTION (>90
111.00	PALERMO	40.00	M	40.00	GBM	TEMPORAL	TEMPORAL	YES	GROSS TOTAL RESECTION (>90
112.00	PALERMO	66.00	M	50.00	METASTASES	PARIETAL	PARIETAL	NO	
113.00	PALERMO	59.00	M	40.00	GBM	FRONTAL	FRONTAL	YES	GROSS TOTAL RESECTION (>90
114.00	PALERMO	35.00	F	50.00	METASTASES			NO	
115.00	PALERMO	53.00	F	80.00	OTHER (ATIPIC MENINGIOMI)			NO	
116.00	PALERMO	51.00	M	90.00	OTHER (ATIPIC MENINGIOMI)	PARIETAL	PARIETAL	YES	GROSS TOTAL RESECTION (>90
117.00	PALERMO	25.00	F	90.00	ASTRO II	FRONTAL	FRONTAL	NO	
118.00	PALERMO	29.00	F	40.00	OLIGOASTROCITOMA III	FRONTAL	FRONTAL	YES	GROSS TOTAL RESECTION (>90
119.00	PALERMO	48.00	M	40.00	ASTROC III	MULTICENTRIC	MULTICENTRIC	YES	GROSS TOTAL RESECTION (>90
120.00	PALERMO	23.00	F	80.00	OTHER (ATIPIC MENINGIOMI)	BILATERAL	MULTICENTRIC	YES	GROSS TOTAL RESECTION (>90
121.00	PERUGIA	80.00	F	60.00	GBM	MULTICENTRIC	MULTICENTRIC	YES	PARTIAL RESECTION (<90%)
122.00	PERUGIA	48.00	F	70.00	ASTRO II	MULTILOBULAR	MULTICENTRIC	YES	PARTIAL RESECTION (<90%)
123.00	PERUGIA	58.00	M	50.00	GBM	FRONTAL	FRONTAL	YES	GROSS TOTAL RESECTION (>90

124.00	PERUGIA	57.00	F	80.00	METASTASES	BILATERAL	MULTICENTRIC	NO	
125.00	PERUGIA	55.00	M	100.00	GBM	TEMPORAL	TEMPORAL	YES	GROSS TOTAL RESECTION (>90
126.00	PERUGIA	63.00	F	60.00	OLIGODENDROGLIOMA II	MULTILOBULAR	MULTICENTRIC	YES	PARTIAL RESECTION (<90%)
127.00	PERUGIA	43.00	F	100.00	OTHER (ATIPIC MENINGIOMI)	FRONTAL	FRONTAL	YES	GROSS TOTAL RESECTION (>90
128.00	PERUGIA	59.00	M	90.00	GBM	TEMPORAL	TEMPORAL	YES	GROSS TOTAL RESECTION (>90
129.00	PERUGIA	35.00	F	100.00	OTHER (ATIPIC MENINGIOMI)	FRONTAL	FRONTAL	YES	PARTIAL RESECTION (<90%)
130.00	PERUGIA	70.00	M	80.00	GBM	MULTILOBULAR	MULTICENTRIC	YES	BIOPSY
131.00	PERUGIA	33.00	M	80.00	ASTRO II	INSULA	INSULA	YES	GROSS TOTAL RESECTION (>90
132.00	PERUGIA	69.00	F	80.00	GBM	MULTILOBULAR	MULTICENTRIC	YES	GROSS TOTAL RESECTION (>90
133.00	PERUGIA	66.00	M	80.00	METASTASES	MULTILOBULAR	MULTICENTRIC	YES	GROSS TOTAL RESECTION (>90
134.00	PERUGIA	78.00	M	70.00	METASTASES	OCCIPITAL	OCCIPITAL	YES	GROSS TOTAL RESECTION (>90
135.00	PERUGIA	61.00	F	100.00	OTHER (ATIPIC MENINGIOMI)	MULTILOBULAR	MULTICENTRIC	YES	GROSS TOTAL RESECTION (>90
136.00	PERUGIA	64.00	M	100.00	GBM	MULTILOBULAR	MULTICENTRIC	YES	GROSS TOTAL RESECTION (>90
137.00	PERUGIA	52.00	M	100.00	OLIGODENDROGLIOMA II	FRONTAL	FRONTAL	YES	PARTIAL RESECTION (<90%)
138.00	PERUGIA	38.00	M	90.00	GBM	FRONTAL	FRONTAL	YES	GROSS TOTAL RESECTION (>90
139.00	PERUGIA	54.00	M	80.00	GBM	FRONTAL	FRONTAL	YES	GROSS TOTAL RESECTION (>90
140.00	PERUGIA	56.00	F	100.00	OTHER (ATIPIC MENINGIOMI)	MULTILOBULAR	MULTICENTRIC	YES	GROSS TOTAL RESECTION (>90
141.00	PERUGIA	41.00	M	90.00	OTHER (ATIPIC MENINGIOMI)	MULTILOBULAR	MULTICENTRIC	YES	GROSS TOTAL RESECTION (>90
142.00	PERUGIA	63.00	M	80.00	GBM	PARIETAL	PARIETAL	YES	GROSS TOTAL RESECTION (>90
143.00	PERUGIA	57.00	M	70.00	GBM	MULTILOBULAR	MULTICENTRIC	YES	GROSS TOTAL RESECTION (>90
144.00	PERUGIA	31.00	M	40.00	OLIGOASTROCITOMA II	MULTILOBULAR	MULTICENTRIC	YES	GROSS TOTAL RESECTION (>90
145.00	PERUGIA	59.00	M	70.00	GBM	MULTILOBULAR	MULTICENTRIC	YES	GROSS TOTAL RESECTION (>90
146.00	PERUGIA	76.00	F	100.00	OTHER (ATIPIC MENINGIOMI)	PARIETAL	PARIETAL	YES	GROSS TOTAL RESECTION (>90
147.00	PERUGIA	67.00	M	60.00	OLIGODENDROGLIOMA III	TEMPORAL	TEMPORAL	YES	PARTIAL RESECTION (<90%)
148.00	PERUGIA	49.00	M	100.00	OTHER (ATIPIC MENINGIOMI)	FRONTAL	FRONTAL	YES	GROSS TOTAL RESECTION (>90
149.00	PERUGIA	52.00	M	90.00	METASTASES	MULTILOBULAR	MULTICENTRIC	NO	
150.00	PERUGIA	41.00	F	60.00	OTHER (ATIPIC MENINGIOMI)	FRONTAL	FRONTAL	YES	GROSS TOTAL RESECTION (>90
151.00	PERUGIA	48.00	F	80.00	OLIGOASTROCITOMA III	FRONTAL	FRONTAL	YES	PARTIAL RESECTION (<90%)
152.00	PERUGIA	47.00	F	80.00	OLIGOASTROCITOMA III	FRONTAL	FRONTAL	YES	GROSS TOTAL RESECTION (>90
153.00	PERUGIA	48.00	F	70.00	GBM	MULTILOBULAR	MULTICENTRIC	YES	GROSS TOTAL RESECTION (>90
154.00	PERUGIA	70.00	M	60.00	GBM	FRONTAL	FRONTAL	YES	GROSS TOTAL RESECTION (>90
155.00	PERUGIA	45.00	M	70.00	GBM	FRONTAL	FRONTAL	YES	GROSS TOTAL RESECTION (>90
156.00	PERUGIA	45.00	F	90.00	OLIGOASTROCITOMA III	FRONTAL	FRONTAL	YES	GROSS TOTAL RESECTION (>90
157.00	PERUGIA	61.00	F	60.00	OLIGODENDROGLIOMA II	MULTILOBULAR	MULTICENTRIC	YES	GROSS TOTAL RESECTION (>90
158.00	PERUGIA	53.00	F	90.00	METASTASES	MULTICENTRIC	MULTICENTRIC	NO	
159.00	PERUGIA	72.00	M	90.00	ASTROC III	PARIETAL	PARIETAL	YES	GROSS TOTAL RESECTION (>90
160.00	PERUGIA	37.00	F	100.00	OLIGODENDROGLIOMA II	MULTILOBULAR	MULTICENTRIC	YES	GROSS TOTAL RESECTION (>90
161.00	PERUGIA	73.00	M	60.00	OLIGODENDROGLIOMA II	INSULA	INSULA	NO	
162.00	PERUGIA	72.00	M	70.00	GBM	FRONTAL	FRONTAL	YES	GROSS TOTAL RESECTION (>90
163.00	PERUGIA	50.00	M	70.00	OLIGODENDROGLIOMA III	MULTILOBULAR	MULTICENTRIC	YES	BIOPSY
164.00	PERUGIA	55.00	M	80.00	OLIGODENDROGLIOMA II	TEMPORAL	TEMPORAL	YES	GROSS TOTAL RESECTION (>90
165.00	PERUGIA	55.00	M	70.00	OLIGODENDROGLIOMA II	MULTILOBULAR	MULTICENTRIC	YES	GROSS TOTAL RESECTION (>90

166.00	PERUGIA	78.00	M	60.00	GBM	MULTICENTRIC	MULTICENTRIC	NO	
167.00	PERUGIA	50.00	M	70.00	GBM	MULTILOBULAR	MULTICENTRIC	YES	PARTIAL RESECTION (<90%)
168.00	PERUGIA	70.00	M	60.00	GBM	TEMPORAL	TEMPORAL	YES	GROSS TOTAL RESECTION (>90
169.00	PERUGIA	40.00	F	50.00	METASTASES	MULTICENTRIC	MULTICENTRIC	YES	PARTIAL RESECTION (<90%)
170.00	PERUGIA	44.00	F	50.00	GBM	BILATERAL	MULTICENTRIC	YES	BIOPSY
171.00	PERUGIA	45.00	F	100.00	OLIGODENDROGLIOMA II	BILATERAL	MULTICENTRIC	YES	PARTIAL RESECTION (<90%)
172.00	PERUGIA	59.00	F	90.00	OTHER (ATIPIC MENINGIOMI)	PARIETAL	PARIETAL	YES	GROSS TOTAL RESECTION (>90
173.00	PERUGIA	29.00	M	90.00	OLIGODENDROGLIOMA III	FRONTAL	FRONTAL	YES	GROSS TOTAL RESECTION (>90
174.00	PERUGIA	53.00	M	100.00	OTHER (ATIPIC MENINGIOMI)	FRONTAL	FRONTAL	YES	GROSS TOTAL RESECTION (>90
175.00	PERUGIA	56.00	F	80.00	GBM	MULTICENTRIC	MULTICENTRIC	YES	PARTIAL RESECTION (<90%)
176.00	PERUGIA	56.00	M	100.00	GBM	FRONTAL	FRONTAL	YES	GROSS TOTAL RESECTION (>90
177.00	PERUGIA	58.00	M	70.00	METASTASES	BILATERAL	MULTICENTRIC	NO	
178.00	REGGIO CALABRIA	39.00	F	40.00	OTHER (ATIPIC MENINGIOMI)	MULTILOBULAR	MULTICENTRIC	YES	PARTIAL RESECTION (<90%)
179.00	REGGIO CALABRIA	68.00	M	90.00	METASTASES	PARIETAL	PARIETAL	NO	
180.00	REGGIO CALABRIA	65.00	M	90.00	GBM	TEMPORAL	TEMPORAL	YES	GROSS TOTAL RESECTION (>90
181.00	REGGIO CALABRIA	61.00	M	90.00	METASTASES	MULTILOBULAR	MULTICENTRIC	NO	
182.00	REGGIO CALABRIA	80.00	F	30.00	ASTRO II	BILATERAL	MULTICENTRIC	YES	BIOPSY
183.00	REGGIO CALABRIA	68.00	F	90.00	GBM	MULTILOBULAR	MULTICENTRIC	YES	GROSS TOTAL RESECTION (>90
184.00	REGGIO CALABRIA	83.00	F	40.00	METASTASES	FRONTAL	FRONTAL	NO	
185.00	REGGIO CALABRIA	62.00	M	50.00	ASTRO II	MULTILOBULAR	MULTICENTRIC	YES	GROSS TOTAL RESECTION (>90
186.00	REGGIO CALABRIA	46.00	M	100.00		TEMPORAL	TEMPORAL	YES	PARTIAL RESECTION (<90%)
187.00	REGGIO CALABRIA	71.00	M	20.00	METASTASES	MULTICENTRIC	MULTICENTRIC	NO	
188.00	REGGIO CALABRIA	39.00	M	90.00	GBM	TEMPORAL	TEMPORAL	YES	PARTIAL RESECTION (<90%)
189.00	REGGIO CALABRIA	33.00	M	90.00	ASTROC III	MULTILOBULAR	MULTICENTRIC	YES	GROSS TOTAL RESECTION (>90
190.00	REGGIO CALABRIA	43.00	M	100.00	ASTRO II	MULTILOBULAR	MULTICENTRIC	YES	PARTIAL RESECTION (<90%)
191.00	REGGIO CALABRIA	28.00	M	100.00	OLIGODENDROGLIOMA III	TEMPORAL	TEMPORAL	YES	GROSS TOTAL RESECTION (>90
192.00	REGGIO CALABRIA	65.00	M	70.00	GBM	PARIETAL	PARIETAL	YES	
193.00	OPBG	19.00	M	90.00	GBM	FRONTAL+PARIETAL	FRONTAL+PARIET	YES	BIOPSY
194.00	OPBG	1.50	F	100.00	GBM	FRONTAL	FRONTAL	YES	GROSS TOTAL RESECTION (>90
195.00	OPBG	13.00	F	100.00	ASTRO II	TEMPORAL	TEMPORAL	YES	GROSS TOTAL RESECTION (>90
196.00	OPBG	3.50	F	100.00	OTHER (ATIPIC MENINGIOMI)	FRONTAL+INSULA	FRONTAL+INSUL/	YES	GROSS TOTAL RESECTION (>90
197.00	OPBG	0.60	F	100.00	ASTRO II	TEMPORAL	TEMPORAL	YES	PARTIAL RESECTION (<90%)
198.00	OPBG	1.00	M	60.00	OTHER (ATIPIC MENINGIOMI)	MULTILOBULAR	MULTILOBULAR	YES	BIOPSY
199.00	OPBG	11.00	M	100.00	OTHER (ATIPIC MENINGIOMI)	FRONTAL	FRONTAL	YES	GROSS TOTAL RESECTION (>90
200.00	ROMA-IRE	28.00	M	90.00	OLIGODENDROGLIOMA III	FRONTAL	FRONTAL	YES	PARTIAL RESECTION (<90%)
201.00	ROMA-IRE	50.00	M	90.00	GBM	FRONTAL	FRONTAL	NO	
202.00	ROMA-IRE	68.00	F	100.00	OTHER (ATIPIC MENINGIOMI)	FRONTAL	FRONTAL	YES	GROSS TOTAL RESECTION (>90
203.00	ROMA-IRE	66.00	F	100.00	OTHER (ATIPIC MENINGIOMI)	FRONTAL	FRONTAL	NO	
204.00	ROMA-IRE	55.00	F	100.00	OTHER (ATIPIC MENINGIOMI)	FRONTAL	FRONTAL	YES	GROSS TOTAL RESECTION (>90
205.00	ROMA-IRE	36.00	M	90.00	OTHER (ATIPIC MENINGIOMI)	FRONTAL	FRONTAL	YES	GROSS TOTAL RESECTION (>90
206.00	ROMA-IRE	67.00	F	100.00	ASTRO II	FRONTAL	FRONTAL	YES	PARTIAL RESECTION (<90%)
207.00	ROMA-IRE	47.00	F	100.00	OLIGODENDROGLIOMA II	FRONTAL	FRONTAL	YES	GROSS TOTAL RESECTION (>90

208.00	ROMA-IRE	21.00	F	100.00	ASTRO II	FRONTAL	FRONTAL	YES	GROSS TOTAL RESECTION (>90
209.00	ROMA-IRE	54.00	F	100.00	OLIGODENDROGLIOMA II	FRONTAL	FRONTAL	YES	GROSS TOTAL RESECTION (>90
210.00	ROMA-IRE	63.00	M	80.00	GBM	FRONTAL	FRONTAL	YES	BIOPSY
211.00	ROMA-IRE	41.00	M	100.00	OLIGODENDROGLIOMA II	FRONTAL	FRONTAL	YES	BIOPSY
212.00	ROMA-IRE	60.00	F	100.00	OTHER (ATIPIC MENINGIOMI)	FRONTAL	FRONTAL	YES	BIOPSY
213.00	ROMA-IRE	42.00	F	100.00	OTHER (ATIPIC MENINGIOMI)	FRONTAL	FRONTAL	YES	GROSS TOTAL RESECTION (>90
214.00	ROMA-IRE	70.00	M	100.00	OTHER (ATIPIC MENINGIOMI)	FRONTAL	FRONTAL	YES	GROSS TOTAL RESECTION (>90
215.00	ROMA-IRE	61.00	F	100.00	OTHER (ATIPIC MENINGIOMI)	FRONTAL	FRONTAL	YES	GROSS TOTAL RESECTION (>90
216.00	ROMA-IRE	70.00	F	50.00	OLIGODENDROGLIOMA III	FRONTAL	FRONTAL	YES	GROSS TOTAL RESECTION (>90
217.00	ROMA-IRE	70.00	F	100.00	OTHER (ATIPIC MENINGIOMI)	FRONTAL	FRONTAL	YES	GROSS TOTAL RESECTION (>90
218.00	ROMA-IRE	33.00	M	70.00	OLIGOASTROCITOMA II	FRONTAL	FRONTAL	YES	GROSS TOTAL RESECTION (>90
219.00	ROMA-IRE	65.00	M	100.00	GBM	FRONTAL	FRONTAL	NO	
220.00	ROMA-IRE	50.00	F	100.00	GBM	FRONTAL	FRONTAL	YES	GROSS TOTAL RESECTION (>90
221.00	ROMA-IRE	23.00	M	100.00	GBM	FRONTAL	FRONTAL	YES	PARTIAL RESECTION (<90%)
222.00	ROMA-IRE	49.00	F	100.00	OTHER (ATIPIC MENINGIOMI)	FRONTAL	FRONTAL	YES	GROSS TOTAL RESECTION (>90
223.00	ROMA-IRE	40.00	M	100.00	ASTROC III	FRONTAL	FRONTAL	NO	
224.00	ROMA-IRE	20.00	F	100.00	ASTROC III	FRONTAL+TEMPORA	FRONTAL+TEMPO	YES	BIOPSY
225.00	ROMA-IRE	44.00	M	80.00	OLIGOASTROCITOMA III	FRONTAL+TEMPORA	FRONTAL+TEMPO	YES	PARTIAL RESECTION (<90%)
226.00	ROMA-IRE	51.00	F	100.00	OLIGOASTROCITOMA II	FRONTAL+TEMPORA	FRONTAL+TEMPO	YES	BIOPSY
227.00	ROMA-IRE	65.00	F	100.00	OLIGOASTROCITOMA II	FRONTAL+PARIETAL	FRONTAL+PARIET	YES	GROSS TOTAL RESECTION (>90
228.00	ROMA-IRE	55.00	F	80.00	OLIGODENDROGLIOMA II	FRONTAL+PARIETAL	FRONTAL+PARIET	YES	PARTIAL RESECTION (<90%)
229.00	ROMA-IRE	67.00	F	70.00	OTHER (ATIPIC MENINGIOMI)	FRONTAL+PARIETAL	FRONTAL+PARIET	YES	GROSS TOTAL RESECTION (>90
230.00	ROMA-IRE	45.00	F	100.00	OLIGODENDROGLIOMA III	FRONTAL+PARIETAL	FRONTAL+PARIET	YES	GROSS TOTAL RESECTION (>90
231.00	ROMA-IRE	47.00	F	100.00	OLIGOASTROCITOMA III	FRONTAL+PARIETAL	FRONTAL+PARIET	YES	GROSS TOTAL RESECTION (>90
232.00	ROMA-IRE	56.00	M	100.00	OLIGODENDROGLIOMA II	FRONTAL+PARIETAL	FRONTAL+PARIET	YES	BIOPSY
233.00	ROMA-IRE	34.00	F	100.00	ASTRO II	FRONTAL+PARIETAL	FRONTAL+PARIET	YES	PARTIAL RESECTION (<90%)
234.00	ROMA-IRE	51.00	F	70.00	ASTRO II	FRONTAL+PARIETAL	FRONTAL+PARIET	YES	BIOPSY
235.00	ROMA-IRE	56.00	M	100.00	OTHER (ATIPIC MENINGIOMI)	FRONTAL+PARIETAL	FRONTAL+PARIET	YES	GROSS TOTAL RESECTION (>90
236.00	ROMA-IRE	25.00	F	100.00	OTHER (ATIPIC MENINGIOMI)	FRONTAL+OCCIPITA	FRONTAL+OCCIPI	YES	PARTIAL RESECTION (<90%)
237.00	ROMA-IRE	23.00	M	100.00	ASTRO II	TEMPORAL	TEMPORAL	NO	
238.00	ROMA-IRE	51.00	M	80.00	OLIGODENDROGLIOMA II	TEMPORAL	TEMPORAL	YES	PARTIAL RESECTION (<90%)
239.00	ROMA-IRE	66.00	F	100.00	OTHER (ATIPIC MENINGIOMI)	TEMPORAL	TEMPORAL	YES	GROSS TOTAL RESECTION (>90
240.00	ROMA-IRE	43.00	F	100.00	ASTROC III	TEMPORAL	TEMPORAL	YES	GROSS TOTAL RESECTION (>90
241.00	ROMA-IRE	51.00	F	90.00	OTHER (ATIPIC MENINGIOMI)	TEMPORAL	TEMPORAL	YES	GROSS TOTAL RESECTION (>90
242.00	ROMA-IRE	68.00	F	100.00	GBM	TEMPORAL	TEMPORAL	YES	PARTIAL RESECTION (<90%)
243.00	ROMA-IRE	39.00	M	100.00	OLIGOASTROCITOMA II	TEMPORAL	TEMPORAL	YES	GROSS TOTAL RESECTION (>90
244.00	ROMA-IRE	47.00	F	80.00	OLIGOASTROCITOMA III	TEMPORAL	TEMPORAL	YES	GROSS TOTAL RESECTION (>90
245.00	ROMA-IRE	71.00	F	100.00	OTHER (ATIPIC MENINGIOMI)	TEMPORAL	TEMPORAL	YES	GROSS TOTAL RESECTION (>90
246.00	ROMA-IRE	47.00	M	100.00	ASTRO II	TEMPORAL	TEMPORAL	NO	
247.00	ROMA-IRE	50.00	M	70.00	OTHER (ATIPIC MENINGIOMI)	TEMPORAL	TEMPORAL	YES	GROSS TOTAL RESECTION (>90
248.00	ROMA-IRE	35.00	M	100.00	OLIGODENDROGLIOMA III	TEMPORAL	TEMPORAL	YES	PARTIAL RESECTION (<90%)
249.00	ROMA-IRE	48.00	M	100.00	ASTROC III	TEMPORAL	TEMPORAL	YES	GROSS TOTAL RESECTION (>90

250.00	ROMA-IRE	64.00	M	100.00	OTHER (ATIPIC MENINGIOMI)	TEMPORAL+PARIET/	TEMPORAL+PARI	YES	GROSS TOTAL RESECTION (>90
251.00	ROMA-IRE	34.00	F	100.00	OLIGODENDROGLIOMA III	TEMPORAL+PARIET/	TEMPORAL+PARI	YES	GROSS TOTAL RESECTION (>90
252.00	ROMA-IRE	69.00	F	70.00	GBM	TEMPORAL+PARIET/	TEMPORAL+PARI	YES	PARTIAL RESECTION (<90%)
253.00	ROMA-IRE	22.00	M	70.00	GBM	TEMPORAL+PARIET/	TEMPORAL+PARI	NO	
254.00	ROMA-IRE	68.00	M	70.00	GBM	TEMPORAL+PARIET/	TEMPORAL+PARI	NO	
255.00	ROMA-IRE	63.00	F	70.00	OLIGOASTROCITOMA III	TEMPORAL+OCCIPIT	TEMPORAL+OCCI	YES	GROSS TOTAL RESECTION (>90
256.00	ROMA-IRE	69.00	F	100.00	GBM	PARIETAL	PARIETAL	YES	PARTIAL RESECTION (<90%)
257.00	ROMA-IRE	22.00	F	100.00	ASTROC III	PARIETAL	PARIETAL	NO	
258.00	ROMA-IRE	43.00	M	100.00	OLIGOASTROCITOMA III	PARIETAL	PARIETAL	NO	
259.00	ROMA-IRE	35.00	F	100.00	OLIGOASTROCITOMA III	PARIETAL	PARIETAL	YES	GROSS TOTAL RESECTION (>90
260.00	ROMA-IRE	68.00	M	80.00	GBM	PARIETAL	PARIETAL	YES	PARTIAL RESECTION (<90%)
261.00	ROMA-IRE	31.00	M	80.00	ASTROC III	PARIETAL	PARIETAL	YES	GROSS TOTAL RESECTION (>90
262.00	ROMA-IRE	61.00	F	100.00	OTHER (ATIPIC MENINGIOMI)	PARIETAL	PARIETAL	YES	GROSS TOTAL RESECTION (>90
263.00	ROMA-IRE	78.00	F	100.00	OTHER (ATIPIC MENINGIOMI)	OCCIPITAL	OCCIPITAL	YES	GROSS TOTAL RESECTION (>90
264.00	ROMA-IRE	65.00	F	100.00	GBM	MULTICENTRIC	MULTICENTRIC	YES	GROSS TOTAL RESECTION (>90
265.00	ROMA-IRE	42.00	M	100.00	GBM	MULTICENTRIC	MULTICENTRIC	YES	GROSS TOTAL RESECTION (>90
266.00	ROMA-IRE	48.00	M	100.00	GBM	MULTICENTRIC	MULTICENTRIC	YES	BIOPSY
267.00	ROMA-IRE	57.00	M	40.00	GBM	MULTILOBULAR	MULTICENTRIC	YES	GROSS TOTAL RESECTION (>90
268.00	ROMA-IRE	61.00	F	100.00	GBM	MULTILOBULAR	MULTICENTRIC	YES	PARTIAL RESECTION (<90%)
269.00	ROMA-IRE	31.00	F	100.00	OLIGOASTROCITOMA III	TEMPORAL+PARIET/	TEMPORAL+PARI	NO	
270.00	ROMA-IRE	45.00	M	90.00	OLIGODENDROGLIOMA III	FRONTAL+TEMPOR/	FRONTAL+TEMP(	NO	
271.00	ROMA-IRE	57.00	F	80.00	METASTASES	PARIETAL	PARIETAL	YES	GROSS TOTAL RESECTION (>90
272.00	ROMA-IRE	66.00	M	70.00	METASTASES	MULTICENTRIC	MULTICENTRIC	YES	PARTIAL RESECTION (<90%)
273.00	ROMA-IRE	52.00	M	100.00	METASTASES	MULTICENTRIC	MULTICENTRIC	NO	
274.00	ROMA-IRE	75.00	M	70.00	METASTASES	MULTICENTRIC	MULTICENTRIC	NO	
275.00	ROMA-IRE	49.00	F	90.00	METASTASES	PARIETAL	PARIETAL	YES	GROSS TOTAL RESECTION (>90
276.00	ROMA-IRE	54.00	F	80.00	METASTASES	MULTICENTRIC	MULTICENTRIC	YES	PARTIAL RESECTION (<90%)
277.00	ROMA-IRE	59.00	F	70.00	METASTASES	MULTICENTRIC	MULTICENTRIC	YES	PARTIAL RESECTION (<90%)
278.00	ROMA-IRE	28.00	M	80.00	METASTASES	FRONTAL	FRONTAL	YES	BIOPSY
279.00	ROMA-IRE	60.00	M	90.00	METASTASES	FRONTAL	FRONTAL	YES	GROSS TOTAL RESECTION (>90
280.00	ROMA-IRE	71.00	M	100.00	METASTASES	PARIETAL	PARIETAL	YES	PARTIAL RESECTION (<90%)
281.00	ROMA-IRE	45.00	M	90.00	METASTASES	MULTICENTRIC	MULTICENTRIC	NO	
282.00	ROMA-SAN PIETRO	60.00	M	70.00	METASTASES	MULTICENTRIC	MULTICENTRIC	NO	
283.00	ROMA-SAN PIETRO	60.00	F	60.00	METASTASES	PARIETAL	PARIETAL	NO	
284.00	ROMA-SAN PIETRO	47.00	M	90.00	METASTASES	MULTICENTRIC	MULTICENTRIC	NO	
285.00	ROMA-SAN PIETRO	36.00	M	80.00	ASTRO II	TEMPORAL	TEMPORAL	YES	GROSS TOTAL RESECTION (>90
286.00	ROMA-SAN PIETRO	65.00	F	70.00	GBM	FRONTAL	FRONTAL	YES	PARTIAL RESECTION (<90%)
287.00	ROMA-SAN PIETRO	71.00	F	80.00	METASTASES	MULTICENTRIC	MULTICENTRIC	NO	
288.00	ROMA-SAN PIETRO	76.00	F	70.00	METASTASES	FRONTAL	FRONTAL	NO	
289.00	ROMA-SAN PIETRO	72.00	M	60.00	METASTASES	FRONTAL	FRONTAL	YES	GROSS TOTAL RESECTION (>90
290.00	ROMA-SAN PIETRO	58.00	F	40.00	METASTASES	MULTICENTRIC	MULTICENTRIC	NO	
291.00	ROMA-SAN PIETRO	55.00	M	70.00	METASTASES	MULTICENTRIC	MULTICENTRIC	NO	

292.00	ROMA-SAN PIETRO	51.00	F	60.00	METASTASES	PARIETAL+OCCIPITA	PARIETAL+OCCIP	YES	PARTIAL RESECTION (<90%)
293.00	ROMA-SAN PIETRO	56.00	F	90.00	METASTASES	MULTICENTRIC	MULTICENTRIC	NO	
294.00	ROMA-S.EUGENIO	37.00	M		ASTRO II	FRONTAL	FRONTAL	YES	GROSS TOTAL RESECTION (>90
295.00	ROMA-S.EUGENIO	75.00	F		OTHER (ATIPIC MENINGIOMI)	MULTICENTRIC	MULTICENTRIC	NO	
296.00	ROMA-S.EUGENIO	36.00	M		ASTRO II	FRONTAL	FRONTAL	YES	GROSS TOTAL RESECTION (>90
297.00	ROMA-S.EUGENIO	70.00	M		METASTASES	MULTICENTRIC	MULTICENTRIC	NO	
298.00	ROMA-S.EUGENIO	59.00	M		METASTASES	PARIETAL	PARIETAL	NO	
299.00	SASSARI-UOC NPI	5.00	M	90.00	OTHER (ATIPIC MENINGIOMI)	FRONTAL	FRONTAL	YES	GROSS TOTAL RESECTION (>90
300.00	ROMA SANT'ANDRE/	63.00	M	90.00	OTHER (ATIPIC MENINGIOMI)	FRONTAL	FRONTAL	NO	
301.00	ROMA SANT'ANDRE/	54.00	M	40.00	ASTROC III	FRONTAL	FRONTAL	YES	GROSS TOTAL RESECTION (>90
302.00	ROMA SANT'ANDRE/	64.00	F	100.00	OTHER (ATIPIC MENINGIOMI)	FRONTAL	FRONTAL	YES	GROSS TOTAL RESECTION (>90
303.00	ROMA SANT'ANDRE/	53.00	F	100.00	METASTASES			NO	
304.00	ROMA SANT'ANDRE/	43.00	F	100.00	OTHER (ATIPIC MENINGIOMI)	TEMPORAL	TEMPORAL	YES	GROSS TOTAL RESECTION (>90
305.00	ROMA SANT'ANDRE/	63.00	F	50.00	GBM	PARIETAL	PARIETAL	YES	GROSS TOTAL RESECTION (>90
306.00	ROMA SANT'ANDRE/	80.00	F	100.00	OTHER (ATIPIC MENINGIOMI)	FRONTAL	FRONTAL	YES	GROSS TOTAL RESECTION (>90
307.00	ROMA SANT'ANDRE/	47.00	F	100.00	METASTASES			NO	
308.00	ROMA SANT'ANDRE/	77.00	M	100.00	OTHER (ATIPIC MENINGIOMI)	TEMPORAL	TEMPORAL	YES	GROSS TOTAL RESECTION (>90
309.00	ROMA SANT'ANDRE/	46.00	F	90.00	GBM	PARIETAL	PARIETAL	YES	GROSS TOTAL RESECTION (>90
310.00	ROMA SANT'ANDRE/	31.00	M	100.00	OTHER (ATIPIC MENINGIOMI)	PARIETAL	PARIETAL	NO	
311.00	ROMA SANT'ANDRE/	80.00	F	100.00	OTHER (ATIPIC MENINGIOMI)	TEMPORAL	TEMPORAL	YES	GROSS TOTAL RESECTION (>90
312.00	ROMA SANT'ANDRE/	68.00	F	100.00	OTHER (ATIPIC MENINGIOMI)	FRONTAL	FRONTAL	YES	GROSS TOTAL RESECTION (>90
313.00	ROMA SANT'ANDRE/	67.00	F	70.00	GBM	FRONTAL	FRONTAL	YES	GROSS TOTAL RESECTION (>90
314.00	ROMA SANT'ANDRE/	66.00	M	70.00	OLIGODENDROGLIOMA II	FRONTAL	FRONTAL	YES	GROSS TOTAL RESECTION (>90
315.00	ROMA SANT'ANDRE/	71.00	M	100.00	GBM	PARIETAL	PARIETAL	YES	GROSS TOTAL RESECTION (>90
316.00	ROMA SANT'ANDRE/	39.00	F	80.00	GBM	PARIETAL	PARIETAL	YES	GROSS TOTAL RESECTION (>90
317.00	ROMA SANT'ANDRE/	24.00	M	100.00	OTHER (ATIPIC MENINGIOMI)	TEMPORAL	TEMPORAL	YES	GROSS TOTAL RESECTION (>90
318.00	ROMA SANT'ANDRE/	63.00	M	100.00	OLIGOASTROCITOMA II			YES	GROSS TOTAL RESECTION (>90
319.00	ROMA SANT'ANDRE/	36.00	M	100.00	ASTRO II	FRONTAL	FRONTAL	YES	GROSS TOTAL RESECTION (>90
320.00	ROMA SANT'ANDRE/	35.00	M	100.00	ASTROC III	MULTILOBULAR	MULTICENTRIC	YES	BIOPSY
321.00	ROMA SANT'ANDRE/	86.00	M	70.00	ASTRO II	PARIETAL	PARIETAL	YES	GROSS TOTAL RESECTION (>90
322.00	ROMA SANT'ANDRE/	74.00	M	100.00	OTHER (ATIPIC MENINGIOMI)	FRONTAL	FRONTAL	NO	
323.00	ROMA SANT'ANDRE/	80.00	F	70.00	GBM	FRONTAL	FRONTAL	YES	
324.00	ROMA SANT'ANDRE/	70.00	F	70.00	GBM	FRONTAL	FRONTAL	YES	GROSS TOTAL RESECTION (>90
325.00	ROMA SANT'ANDRE/	60.00	F	90.00	ASTRO II	PARIETAL	PARIETAL	YES	GROSS TOTAL RESECTION (>90
326.00	ROMA SANT'ANDRE/	56.00	F	100.00	GBM	FRONTAL	FRONTAL	YES	GROSS TOTAL RESECTION (>90
327.00	ROMA SANT'ANDRE/	66.00	F	80.00	METASTASES			YES	GROSS TOTAL RESECTION (>90
328.00	ROMA SANT'ANDRE/	57.00	M	90.00	OTHER (ATIPIC MENINGIOMI)	FRONTAL	FRONTAL	YES	GROSS TOTAL RESECTION (>90
329.00	ROMA SANT'ANDRE/	48.00	M	100.00	OLIGODENDROGLIOMA II	FRONTAL	FRONTAL	YES	BIOPSY
330.00	ROMA SANT'ANDRE/	72.00	M	80.00	GBM	PARIETAL	PARIETAL	YES	GROSS TOTAL RESECTION (>90
331.00	TORINO-Osp.Infantil	9.00	M	90.00	ASTROC III	TEMPORAL	TEMPORAL	YES	PARTIAL RESECTION (<90%)
332.00	TORINO-Osp.Infantil	3.00	M	100.00	OTHER (ATIPIC MENINGIOMI)	PARIETAL	PARIETAL	YES	GROSS TOTAL RESECTION (>90
333.00	TORINO-Osp.Infantil	9.00	F	80.00	ASTRO II	MULTILOBULAR	MULTICENTRIC	YES	PARTIAL RESECTION (<90%)

334.00	TORINO-Osp.Infantil	12.00	F	90.00	OTHER (ATIPIC MENINGIOMI)	FRONTAL+PARIETAL	FRONTAL+PARIET	YES	GROSS TOTAL RESECTION (>90
335.00	TORINO-Osp.Infantil	14.00	M	60.00	OTHER (ATIPIC MENINGIOMI)	MULTICENTRIC	MULTICENTRIC	YES	PARTIAL RESECTION (<90%)
336.00	Napoli	34.00	M		OLIGODENDROGLIOMA III	FRONTAL	FRONTAL	YES	GROSS TOTAL RESECTION (>90
337.00	Napoli	21.00	F		ASTRO II	MULTICENTRIC	MULTICENTRIC	YES	PARTIAL RESECTION (<90%)
338.00	Napoli	47.00	F			TEMPORAL	TEMPORAL	YES	PARTIAL RESECTION (<90%)
339.00	Napoli	18.00	F				TEMPORAL+PARI	YES	PARTIAL RESECTION (<90%)
340.00	Napoli	48.00	M		GBM	FRONTAL	FRONTAL	YES	PARTIAL RESECTION (<90%)
341.00	Napoli	26.00	M		OLIGODENDROGLIOMA III	FRONTAL	FRONTAL	YES	GROSS TOTAL RESECTION (>90
342.00	Napoli	10.00	M		ASTRO II	FRONTAL	FRONTAL	YES	PARTIAL RESECTION (<90%)
343.00	Napoli	16.00	M		ASTROC III		FRONTAL+TEMP(	YES	GROSS TOTAL RESECTION (>90
344.00	Napoli	8.00	M		ASTRO II	TEMPORAL	TEMPORAL	YES	GROSS TOTAL RESECTION (>90
345.00	Napoli	8.00	F		ASTROC III	FRONTAL	FRONTAL	YES	GROSS TOTAL RESECTION (>90
346.00	Napoli	3.00	F		ASTRO II		FRONTAL+PARIET	YES	PARTIAL RESECTION (<90%)
347.00	Napoli	13.00	F		OLIGODENDROGLIOMA II	TEMPORAL	TEMPORAL	YES	GROSS TOTAL RESECTION (>90
348.00	Napoli	16.00	M		ASTRO II	TEMPORAL	TEMPORAL	YES	GROSS TOTAL RESECTION (>90
349.00	Napoli	26.00	F		OLIGODENDROGLIOMA II	PARIETAL	PARIETAL	YES	PARTIAL RESECTION (<90%)
350.00	Napoli		F		ASTRO II			YES	PARTIAL RESECTION (<90%)
351.00	Napoli	51.00	M		ASTRO II	PARIETAL	PARIETAL	NO	
352.00	Napoli	13.00	M		OLIGODENDROGLIOMA II	OCCIPITAL	OCCIPITAL	YES	GROSS TOTAL RESECTION (>90
353.00	Napoli	68.00	M		GBM		TEMPORAL+PARI	YES	GROSS TOTAL RESECTION (>90
354.00	UDINE PAULETTO	33.00	M	90.00	OLIGOASTROCITOMA II	FRONTAL	FRONTAL	YES	GROSS TOTAL RESECTION (>90
355.00	UDINE PAULETTO	36.00	M	100.00	OLIGOASTROCITOMA II	FRONTAL	FRONTAL	YES	GROSS TOTAL RESECTION (>90
356.00	UDINE PAULETTO	28.00	M	90.00	OLIGOASTROCITOMA III	MULTICENTRIC	MULTICENTRIC	YES	PARTIAL RESECTION (<90%)
357.00	UDINE PAULETTO	38.00	M	100.00	ASTRO II	TEMPORAL	TEMPORAL	YES	GROSS TOTAL RESECTION (>90
358.00	UDINE PAULETTO	37.00	M	100.00	OLIGODENDROGLIOMA II	TEMPORAL	TEMPORAL	YES	GROSS TOTAL RESECTION (>90
359.00	UDINE PAULETTO	35.00	M	100.00	ASTRO II	INSULA	INSULA	YES	PARTIAL RESECTION (<90%)
360.00	UDINE PAULETTO	41.00	M	90.00	ASTRO II	PARIETAL	PARIETAL	YES	GROSS TOTAL RESECTION (>90
361.00	UDINE PAULETTO	58.00	F	80.00	GBM	TEMPORAL	TEMPORAL	YES	GROSS TOTAL RESECTION (>90
362.00	UDINE PAULETTO	52.00	F	80.00	ASTROC III	FRONTAL	FRONTAL	YES	GROSS TOTAL RESECTION (>90
363.00	UDINE PAULETTO	37.00	F	90.00	ASTROC III	INSULA	INSULA	YES	GROSS TOTAL RESECTION (>90
364.00	UDINE PAULETTO	40.00	F	80.00	OLIGOASTROCITOMA III	PARIETAL	PARIETAL	YES	GROSS TOTAL RESECTION (>90
365.00	UDINE PAULETTO	47.00	M	100.00	OLIGOASTROCITOMA II	INSULA	INSULA	YES	GROSS TOTAL RESECTION (>90
366.00	UDINE PAULETTO	44.00	M	90.00	OLIGODENDROGLIOMA II	FRONTAL	FRONTAL	YES	GROSS TOTAL RESECTION (>90
367.00	UDINE PAULETTO	38.00	F	90.00	OLIGODENDROGLIOMA II	INSULA	INSULA	YES	GROSS TOTAL RESECTION (>90
368.00	UDINE PAULETTO	45.00	M	80.00	OLIGODENDROGLIOMA III	INSULA	INSULA	YES	GROSS TOTAL RESECTION (>90
369.00	UDINE PAULETTO	63.00	F	60.00	OLIGODENDROGLIOMA II	FRONTAL	FRONTAL	YES	GROSS TOTAL RESECTION (>90
370.00	UDINE PAULETTO	44.00	M	80.00	GBM	TEMPORAL	TEMPORAL	YES	PARTIAL RESECTION (<90%)
371.00	UDINE PAULETTO	47.00	F	100.00	OLIGODENDROGLIOMA II	FRONTAL	FRONTAL	YES	GROSS TOTAL RESECTION (>90
372.00	UDINE PAULETTO	41.00	F	90.00	OLIGODENDROGLIOMA II	FRONTAL	FRONTAL	YES	GROSS TOTAL RESECTION (>90
373.00	UDINE PAULETTO	52.00	M	90.00	GBM	TEMPORAL	TEMPORAL	YES	GROSS TOTAL RESECTION (>90
374.00	UDINE PAULETTO	41.00	F	90.00	ASTROC III	FRONTAL	FRONTAL	YES	GROSS TOTAL RESECTION (>90
375.00	UDINE PAULETTO	58.00	M	100.00	ASTRO II	PARIETAL	PARIETAL	YES	BIOPSY

376.00	UDINE PAULETTO	31.00	F	90.00	ASTRO II	FRONTAL	FRONTAL	YES	BIOPSY
377.00	UDINE PAULETTO	38.00	M	80.00	ASTRO II	INSULA	INSULA	YES	BIOPSY
378.00	ROMA-LA SAPIENZA-	55.00	M	60.00	GBM	FRONTAL	FRONTAL	YES	GROSS TOTAL RESECTION (>90
379.00	ROMA-LA SAPIENZA-	59.00	F	60.00	GBM	FRONTAL	FRONTAL	YES	GROSS TOTAL RESECTION (>90
380.00	ROMA-LA SAPIENZA-	61.00	F	70.00	GBM	FRONTAL	FRONTAL	YES	GROSS TOTAL RESECTION (>90
381.00	ROMA-LA SAPIENZA-	62.00	F	90.00	GBM	TEMPORAL	TEMPORAL	YES	GROSS TOTAL RESECTION (>90
382.00	ROMA-LA SAPIENZA-	67.00	M	90.00	GBM	TEMPORAL	TEMPORAL	YES	GROSS TOTAL RESECTION (>90
383.00	ROMA-LA SAPIENZA-	34.00	F	80.00	GBM	PARIETAL+OCCIPITA	PARIETAL+OCCIP	YES	GROSS TOTAL RESECTION (>90
384.00	ROMA-LA SAPIENZA-	62.00	M	60.00	GBM	FRONTAL	FRONTAL	YES	PARTIAL RESECTION (<90%)
385.00	ROMA-LA SAPIENZA-	47.00	M	90.00	GBM	TEMPORAL	TEMPORAL	YES	BIOPSY
386.00	ROMA-LA SAPIENZA-	32.00	F	80.00	GBM	FRONTAL+TEMPOR/	FRONTAL+TEMP(	YES	PARTIAL RESECTION (<90%)
387.00	ROMA-LA SAPIENZA-	73.00	M	70.00	GBM	TEMPORAL	TEMPORAL	YES	GROSS TOTAL RESECTION (>90
388.00	ROMA-LA SAPIENZA-	71.00	M	90.00	GBM	TEMPORAL	TEMPORAL	YES	GROSS TOTAL RESECTION (>90
389.00	ROMA-LA SAPIENZA-	64.00	M	70.00	ASTROC III	FRONTAL	FRONTAL	YES	PARTIAL RESECTION (<90%)
390.00	ROMA-LA SAPIENZA-	48.00	M	60.00	GBM	FRONTAL+PARIETAL	FRONTAL+PARIET	YES	GROSS TOTAL RESECTION (>90
391.00	ROMA-LA SAPIENZA-	62.00	M	80.00	GBM	TEMPORAL	TEMPORAL	YES	PARTIAL RESECTION (<90%)
392.00	ROMA-LA SAPIENZA-	53.00	M	100.00	GBM	FRONTAL	FRONTAL	YES	GROSS TOTAL RESECTION (>90
393.00	ROMA-LA SAPIENZA-	36.00	M	100.00	ASTROC III	FRONTAL	FRONTAL	YES	GROSS TOTAL RESECTION (>90
394.00	ROMA-LA SAPIENZA-	71.00	F	90.00	GBM	TEMPORAL	TEMPORAL	YES	GROSS TOTAL RESECTION (>90
395.00	ROMA-LA SAPIENZA-	63.00	F	100.00	ASTROC III	FRONTAL	FRONTAL	YES	GROSS TOTAL RESECTION (>90
396.00	ROMA-LA SAPIENZA-	57.00	M	100.00	GBM	TEMPORAL	TEMPORAL	YES	GROSS TOTAL RESECTION (>90
397.00	ROMA-LA SAPIENZA-	56.00	M	70.00	GBM	PARIETAL	PARIETAL	YES	GROSS TOTAL RESECTION (>90
398.00	ROMA-LA SAPIENZA-	58.00	M	100.00	ASTROC III	FRONTAL+TEMPOR/	FRONTAL+TEMP(	YES	PARTIAL RESECTION (<90%)
399.00	ROMA-LA SAPIENZA-	29.00	M	90.00	OLIGODENDROGLIOMA II	FRONTAL+PARIETAL	FRONTAL+PARIET	YES	PARTIAL RESECTION (<90%)
400.00	ROMA-LA SAPIENZA-	72.00	F	60.00	GBM	FRONTAL	FRONTAL	YES	GROSS TOTAL RESECTION (>90
401.00	ROMA-LA SAPIENZA-	70.00	M	80.00	GBM	PARIETAL	PARIETAL	YES	GROSS TOTAL RESECTION (>90
402.00	ROMA-LA SAPIENZA-	61.00	F	60.00	GBM	FRONTAL	FRONTAL	YES	GROSS TOTAL RESECTION (>90
403.00	ROMA-LA SAPIENZA-	52.00	M	70.00	GBM	PARIETAL	PARIETAL	YES	GROSS TOTAL RESECTION (>90
404.00	ROMA-LA SAPIENZA-	55.00	F	90.00	GBM	TEMPORAL	TEMPORAL	YES	GROSS TOTAL RESECTION (>90
405.00	ROMA-LA SAPIENZA-	22.00	F	100.00	ASTROC III	FRONTAL	FRONTAL	YES	PARTIAL RESECTION (<90%)
406.00	ROMA-LA SAPIENZA-	19.00	M	100.00	GBM	PARIETAL	PARIETAL	YES	GROSS TOTAL RESECTION (>90
407.00	ROMA-LA SAPIENZA-	47.00	M	100.00	GBM	FRONTAL	FRONTAL	YES	GROSS TOTAL RESECTION (>90
408.00	ROMA-LA SAPIENZA-	48.00	M	90.00	GBM	FRONTAL	FRONTAL	YES	GROSS TOTAL RESECTION (>90
409.00	ROMA-LA SAPIENZA-	58.00	F	60.00	GBM	TEMPORAL	TEMPORAL	YES	GROSS TOTAL RESECTION (>90
410.00	ROMA-LA SAPIENZA-	57.00	F	90.00	GBM	PARIETAL	PARIETAL	YES	GROSS TOTAL RESECTION (>90
411.00	ROMA-LA SAPIENZA-	20.00	M	100.00	GBM	FRONTAL	FRONTAL	YES	GROSS TOTAL RESECTION (>90
412.00	VARESE	71.00	F	60.00	GBM	TEMPORAL	TEMPORAL	YES	GROSS TOTAL RESECTION (>90
413.00	VARESE	74.00	F	100.00	OTHER (ATIPIC MENINGIOMI)	FRONTAL	FRONTAL	YES	GROSS TOTAL RESECTION (>90
414.00	VARESE	16.00	F	100.00	OTHER (ATIPIC MENINGIOMI)	FRONTAL	FRONTAL	YES	GROSS TOTAL RESECTION (>90
415.00	VARESE	58.00	M	100.00	OTHER (ATIPIC MENINGIOMI)	FRONTAL	FRONTAL	NO	
416.00	VARESE	61.00	F	100.00	OTHER (ATIPIC MENINGIOMI)	PARIETAL	PARIETAL	YES	GROSS TOTAL RESECTION (>90
417.00	VARESE	23.00	M	90.00	OTHER (ATIPIC MENINGIOMI)	OCCIPITAL	OCCIPITAL	YES	GROSS TOTAL RESECTION (>90

418.00	VARESE	51.00	F	100.00	OTHER (ATIPIC MENINGIOMI)	PARIETAL	PARIETAL	YES	GROSS TOTAL RESECTION (>90
419.00	VARESE	9.00	M	100.00	OTHER (ATIPIC MENINGIOMI)	OCCIPITAL	OCCIPITAL	YES	GROSS TOTAL RESECTION (>90
420.00	VARESE	58.00	F	100.00	OTHER (ATIPIC MENINGIOMI)	PARIETAL	PARIETAL	YES	GROSS TOTAL RESECTION (>90
421.00	VARESE	19.00	M	100.00	ASTRO II	HYPOTHALAMIC OP	HYPOTHALAMIC	YES	GROSS TOTAL RESECTION (>90
422.00	VARESE	37.00	M	100.00	OLIGODENDROGLIOMA II	PARIETAL	PARIETAL	YES	GROSS TOTAL RESECTION (>90
423.00	VARESE	33.00	M	90.00	OLIGODENDROGLIOMA II	FRONTAL	FRONTAL	YES	GROSS TOTAL RESECTION (>90
424.00	VARESE	39.00	F	100.00	OTHER (ATIPIC MENINGIOMI)	PARIETAL	PARIETAL	YES	GROSS TOTAL RESECTION (>90
425.00	VARESE	79.00	F	100.00	OTHER (ATIPIC MENINGIOMI)	FRONTAL	FRONTAL	NO	
426.00	VARESE	67.00	M	100.00	OTHER (ATIPIC MENINGIOMI)	FRONTAL	FRONTAL	YES	GROSS TOTAL RESECTION (>90
427.00	VARESE	51.00	F	100.00	OTHER (ATIPIC MENINGIOMI)	PARIETAL	PARIETAL	YES	GROSS TOTAL RESECTION (>90
428.00	VARESE	56.00	M	100.00	OTHER (ATIPIC MENINGIOMI)	FRONTAL	FRONTAL	YES	GROSS TOTAL RESECTION (>90
429.00	VARESE	43.00	M	100.00	OTHER (ATIPIC MENINGIOMI)	CEREBELLAR	CEREBELLAR	YES	GROSS TOTAL RESECTION (>90
430.00	VARESE	29.00	F	100.00	GBM	FRONTAL	FRONTAL	YES	GROSS TOTAL RESECTION (>90
431.00	VARESE		M	100.00	OTHER (ATIPIC MENINGIOMI)	FRONTAL	FRONTAL	YES	GROSS TOTAL RESECTION (>90
432.00	VARESE	62.00	M	70.00	GBM	TEMPORAL	TEMPORAL	YES	GROSS TOTAL RESECTION (>90
433.00	VARESE	46.00	M	100.00	OTHER (ATIPIC MENINGIOMI)	TEMPORAL	TEMPORAL	YES	GROSS TOTAL RESECTION (>90
434.00	VARESE	45.00	F	100.00	OTHER (ATIPIC MENINGIOMI)	TEMPORAL	TEMPORAL	YES	GROSS TOTAL RESECTION (>90
435.00	VARESE	68.00	F	100.00	OTHER (ATIPIC MENINGIOMI)	TEMPORAL	TEMPORAL	YES	GROSS TOTAL RESECTION (>90
436.00	VARESE	63.00	F	70.00	GBM	PARIETAL	PARIETAL	YES	GROSS TOTAL RESECTION (>90
437.00	VARESE	64.00	F	100.00	OTHER (ATIPIC MENINGIOMI)	FRONTAL	FRONTAL	YES	GROSS TOTAL RESECTION (>90
438.00	VARESE	61.00	F	100.00	OTHER (ATIPIC MENINGIOMI)	TEMPORAL	TEMPORAL	YES	GROSS TOTAL RESECTION (>90
439.00	VARESE	60.00	F	100.00	OTHER (ATIPIC MENINGIOMI)	PARIETAL	PARIETAL	YES	GROSS TOTAL RESECTION (>90
440.00	VARESE	28.00	F	100.00	OLIGODENDROGLIOMA III	FRONTAL	FRONTAL	YES	GROSS TOTAL RESECTION (>90
441.00	VARESE	31.00	F	100.00	OTHER (ATIPIC MENINGIOMI)	FRONTAL	FRONTAL	YES	GROSS TOTAL RESECTION (>90
442.00	VARESE	27.00	F	80.00	GBM	FRONTAL	FRONTAL	YES	GROSS TOTAL RESECTION (>90
443.00	VARESE	56.00	F	100.00	OTHER (ATIPIC MENINGIOMI)	FRONTAL	FRONTAL	YES	GROSS TOTAL RESECTION (>90
444.00	VARESE	41.00	M	100.00	OLIGODENDROGLIOMA II	FRONTAL	FRONTAL	YES	GROSS TOTAL RESECTION (>90
445.00	VARESE	36.00	M	100.00	OLIGODENDROGLIOMA II	INSULA	INSULA	YES	GROSS TOTAL RESECTION (>90
446.00	VARESE	52.00	M	100.00	OTHER (ATIPIC MENINGIOMI)	OCCIPITAL	OCCIPITAL	YES	GROSS TOTAL RESECTION (>90
447.00	VARESE	49.00	F	100.00	OTHER (ATIPIC MENINGIOMI)	FRONTAL	FRONTAL	YES	GROSS TOTAL RESECTION (>90
448.00	VARESE	12.00	M	100.00	ASTRO II	OCCIPITAL	OCCIPITAL	YES	GROSS TOTAL RESECTION (>90
449.00	VARESE	30.00	F	100.00	OTHER (ATIPIC MENINGIOMI)	PARIETAL	PARIETAL	YES	GROSS TOTAL RESECTION (>90
450.00	VARESE	34.00	M	100.00	OTHER (ATIPIC MENINGIOMI)	CEREBELLAR	CEREBELLAR	YES	GROSS TOTAL RESECTION (>90
451.00	VARESE	53.00	M	100.00	OTHER (ATIPIC MENINGIOMI)	OCCIPITAL	OCCIPITAL	YES	GROSS TOTAL RESECTION (>90
452.00	VARESE	55.00	F	100.00	OTHER (ATIPIC MENINGIOMI)	PARIETAL	PARIETAL	YES	GROSS TOTAL RESECTION (>90
453.00	VARESE	42.00	F	100.00	OTHER (ATIPIC MENINGIOMI)	PARIETAL	PARIETAL	YES	GROSS TOTAL RESECTION (>90
454.00	VARESE	30.00	F	100.00	OTHER (ATIPIC MENINGIOMI)	FRONTAL	FRONTAL	YES	GROSS TOTAL RESECTION (>90
455.00	VARESE	71.00	F	100.00	OTHER (ATIPIC MENINGIOMI)	FRONTAL	FRONTAL	YES	GROSS TOTAL RESECTION (>90
456.00	VARESE	36.00	M	100.00	OTHER (ATIPIC MENINGIOMI)	OCCIPITAL	OCCIPITAL	YES	GROSS TOTAL RESECTION (>90
457.00	VARESE	30.00	M	100.00	ASTRO II	TEMPORAL	TEMPORAL	YES	GROSS TOTAL RESECTION (>90
458.00	VARESE	51.00	F	90.00	GBM	OCCIPITAL	OCCIPITAL	YES	GROSS TOTAL RESECTION (>90
459.00	VARESE	35.00	F	100.00	OLIGODENDROGLIOMA II	FRONTAL	FRONTAL	YES	GROSS TOTAL RESECTION (>90

460.00	VARESE	44.00	F	100.00	METASTASES	OCCIPITAL	OCCIPITAL	YES	GROSS TOTAL RESECTION (>90
461.00	VARESE	70.00	F	100.00	OTHER (ATIPIC MENINGIOMI)	FRONTAL	FRONTAL	YES	GROSS TOTAL RESECTION (>90
462.00	VARESE	68.00	F	100.00	OTHER (ATIPIC MENINGIOMI)	PARIETAL	PARIETAL	YES	GROSS TOTAL RESECTION (>90
463.00	VENEZIA	36.00	M	100.00	GBM	FRONTAL	FRONTAL	YES	GROSS TOTAL RESECTION (>90
464.00	VENEZIA	67.00	M	100.00	GBM	PARIETAL	PARIETAL	YES	GROSS TOTAL RESECTION (>90
465.00	VENEZIA	55.00	M	80.00	ASTROC III	TEMPORAL	TEMPORAL	YES	PARTIAL RESECTION (<90%)
466.00	VENEZIA	64.00	M	100.00	ASTROC III	MULTILOBULAR	MULTICENTRIC	YES	PARTIAL RESECTION (<90%)
467.00	VENEZIA	46.00	F	80.00	GBM	FRONTAL	FRONTAL	YES	PARTIAL RESECTION (<90%)
468.00	VENEZIA	74.00	M	100.00	OLIGODENDROGLIOMA II	FRONTAL	FRONTAL	YES	GROSS TOTAL RESECTION (>90
469.00	VENEZIA	42.00	M	100.00	ASTRO II	FRONTAL	FRONTAL	YES	BIOPSY
470.00	VENEZIA	58.00	F	80.00	METASTASES	MULTILOBULAR	MULTICENTRIC	YES	BIOPSY
471.00	VENEZIA	73.00	M	60.00	METASTASES	MULTILOBULAR	MULTICENTRIC	NO	
472.00	VENEZIA	66.00	F	90.00	GBM	MULTILOBULAR	MULTICENTRIC	YES	BIOPSY
473.00	VERONA	56.00	M	100.00	GBM	PARIETAL	PARIETAL	YES	PARTIAL RESECTION (<90%)
474.00	UCSC Roma	33.00	F	80.00	ASTROC III	FRONTAL	FRONTAL	YES	PARTIAL RESECTION (<90%)
475.00	UCSC Roma	58.00	M	60.00	GBM	PARIETAL	PARIETAL	YES	GROSS TOTAL RESECTION (>90
476.00	UCSC Roma	49.00	M	90.00	OLIGOASTROCITOMA II		PARIETAL+OCCIP	YES	PARTIAL RESECTION (<90%)
477.00	UCSC Roma	46.00	M	100.00	OTHER (ATIPIC MENINGIOMI)	PARIETAL	PARIETAL	YES	GROSS TOTAL RESECTION (>90
478.00	UCSC Roma	50.00	M	60.00	OLIGODENDROGLIOMA III	FRONTAL	FRONTAL	YES	GROSS TOTAL RESECTION (>90
479.00	UCSC Roma	41.00	F	90.00	OLIGOASTROCITOMA III	FRONTAL	FRONTAL	YES	GROSS TOTAL RESECTION (>90
480.00	UCSC Roma	47.00	F	90.00	GBM	TEMPORAL	TEMPORAL	YES	GROSS TOTAL RESECTION (>90
481.00	UCSC Roma	45.00	F	80.00	ASTROC III	FRONTAL	FRONTAL	YES	GROSS TOTAL RESECTION (>90
482.00	UCSC Roma	56.00	F	50.00	OLIGODENDROGLIOMA III	PARIETAL	PARIETAL	YES	PARTIAL RESECTION (<90%)
483.00	UCSC Roma	31.00	M	100.00	GBM		FRONTAL+PARIET	YES	GROSS TOTAL RESECTION (>90
484.00	UCSC Roma	42.00	F	100.00	OLIGODENDROGLIOMA III	BILATERAL	MULTICENTRIC	YES	BIOPSY
485.00	UCSC Roma	34.00	F	60.00	OLIGODENDROGLIOMA II	FRONTAL	FRONTAL	YES	
486.00	UCSC Roma	21.00	M	90.00	ASTROC III	FRONTAL	FRONTAL	YES	GROSS TOTAL RESECTION (>90
487.00	UCSC Roma	62.00	F	80.00	GBM	PARIETAL	PARIETAL	YES	GROSS TOTAL RESECTION (>90
488.00	UCSC Roma	74.00	M	70.00	GBM		TEMPORAL+PARI	YES	GROSS TOTAL RESECTION (>90
489.00	UCSC Roma	63.00	M	30.00	GBM	FRONTAL	FRONTAL	YES	GROSS TOTAL RESECTION (>90
490.00	UCSC Roma	64.00	F	70.00	OLIGODENDROGLIOMA III	TEMPORAL	TEMPORAL	YES	PARTIAL RESECTION (<90%)
491.00	UCSC Roma	68.00	M	50.00	OLIGODENDROGLIOMA III	FRONTAL	FRONTAL	YES	PARTIAL RESECTION (<90%)
492.00	UCSC Roma	55.00	F	40.00	GBM	FRONTAL	FRONTAL	YES	PARTIAL RESECTION (<90%)
493.00	UCSC Roma	42.00	M	100.00	GBM	PARIETAL	PARIETAL	YES	GROSS TOTAL RESECTION (>90
494.00	UCSC Roma	69.00	F	60.00	GBM		FRONTAL+PARIET	YES	PARTIAL RESECTION (<90%)
495.00	UCSC Roma	48.00	F	60.00	GBM		TEMPORAL+PARI	YES	GROSS TOTAL RESECTION (>90
496.00	UCSC Roma	25.00	M	100.00	OLIGOASTROCITOMA III	FRONTAL	FRONTAL	YES	GROSS TOTAL RESECTION (>90
497.00	UCSC Roma	56.00	F	100.00	OLIGOASTROCITOMA III	TEMPORAL	TEMPORAL	YES	GROSS TOTAL RESECTION (>90
498.00	UCSC Roma	40.00	M	100.00	ASTROC III	FRONTAL	FRONTAL	YES	GROSS TOTAL RESECTION (>90
499.00	UCSC Roma	48.00	M	70.00	OLIGOASTROCITOMA III	TEMPORAL	TEMPORAL	YES	GROSS TOTAL RESECTION (>90
500.00	UCSC Roma	58.00	M	80.00	GBM	OCCIPITAL	OCCIPITAL	YES	PARTIAL RESECTION (<90%)
501.00	UCSC Roma	31.00	F	100.00	GBM		FRONTAL+PARIET	YES	GROSS TOTAL RESECTION (>90

502.00	UCSC Roma	61.00	M	40.00	GBM	MULTILOBULAR	MULTICENTRIC	YES	BIOPSY
503.00	UCSC Roma	33.00	M	50.00	GBM	MULTILOBULAR	MULTICENTRIC	YES	PARTIAL RESECTION (<90%)
504.00	UCSC Roma	70.00	M	50.00	GBM	FRONTAL	FRONTAL	YES	GROSS TOTAL RESECTION (>90
505.00	UCSC Roma	57.00	F		GBM	MULTILOBULAR	MULTICENTRIC	YES	BIOPSY
506.00	UCSC Roma	74.00	F	50.00	GBM	MULTILOBULAR	MULTICENTRIC	YES	PARTIAL RESECTION (<90%)
507.00	UCSC Roma	67.00	M	40.00	OTHER (ATIPIC MENINGIOMI)	MULTICENTRIC	MULTICENTRIC	YES	PARTIAL RESECTION (<90%)
508.00	UCSC Roma	42.00	F	60.00	OLIGODENDROGLIOMA III		FRONTAL+PARIET	YES	PARTIAL RESECTION (<90%)
509.00	UCSC Roma	46.00	M	100.00	OLIGOASTROCITOMA III	PARIETAL	PARIETAL	YES	GROSS TOTAL RESECTION (>90
510.00	UCSC Roma	36.00	F	90.00	OLIGOASTROCITOMA III	FRONTAL	FRONTAL	YES	PARTIAL RESECTION (<90%)
511.00	UCSC Roma	51.00	F	80.00		FRONTAL	FRONTAL	YES	PARTIAL RESECTION (<90%)
512.00	UCSC Roma	42.00	F	70.00	ASTRO II		FRONTAL+PARIET	YES	BIOPSY
513.00	UCSC Roma	61.00	F	70.00	OLIGOASTROCITOMA II	FRONTAL	FRONTAL	YES	PARTIAL RESECTION (<90%)
514.00	UCSC Roma	62.00	M	90.00	GBM	FRONTAL	FRONTAL	YES	PARTIAL RESECTION (<90%)
515.00	UCSC Roma	67.00	F	70.00	GBM		TEMPORAL+PARI	YES	GROSS TOTAL RESECTION (>90
516.00	UCSC Roma	54.00	F	100.00	OLIGODENDROGLIOMA III	PARIETAL	PARIETAL	YES	GROSS TOTAL RESECTION (>90
517.00	UCSC Roma	45.00	F	60.00	GBM		FRONTAL+TEMP(	YES	PARTIAL RESECTION (<90%)
518.00	UCSC Roma	35.00	M	80.00	OLIGOASTROCITOMA II	FRONTAL	FRONTAL	YES	GROSS TOTAL RESECTION (>90
519.00	UCSC Roma	42.00	M	90.00	ASTROC III	FRONTAL	FRONTAL	YES	PARTIAL RESECTION (<90%)
520.00	UCSC Roma	50.00	F	50.00		BILATERAL	MULTICENTRIC	YES	PARTIAL RESECTION (<90%)
521.00	UCSC Roma	42.00	F	100.00		FRONTAL	FRONTAL	YES	PARTIAL RESECTION (<90%)
522.00	UCSC Roma	44.00	M	40.00	ASTROC III	FRONTAL	FRONTAL	YES	PARTIAL RESECTION (<90%)
523.00	UCSC Roma	57.00	F	90.00	OTHER (ATIPIC MENINGIOMI)		FRONTAL+OCCIPI	YES	GROSS TOTAL RESECTION (>90
524.00	UCSC Roma	55.00	M	40.00		TEMPORAL	TEMPORAL	YES	PARTIAL RESECTION (<90%)
525.00	UCSC Roma	53.00	M	70.00	GBM	TEMPORAL	TEMPORAL	YES	PARTIAL RESECTION (<90%)
526.00	UCSC Roma	74.00	M			FRONTAL	FRONTAL	NO	
527.00	UCSC Roma	41.00	M	70.00	OLIGODENDROGLIOMA III	FRONTAL	FRONTAL	YES	GROSS TOTAL RESECTION (>90
528.00	UCSC Roma	56.00	F	70.00	METASTASES		TEMPORAL+INSU	YES	PARTIAL RESECTION (<90%)
529.00	UCSC Roma	76.00	F	70.00	OLIGOASTROCITOMA III	TEMPORAL	TEMPORAL	YES	GROSS TOTAL RESECTION (>90
530.00	UCSC Roma	75.00	M	70.00	OTHER (ATIPIC MENINGIOMI)	BILATERAL	MULTICENTRIC	YES	PARTIAL RESECTION (<90%)
531.00	UCSC Roma	37.00	M	80.00	OLIGOASTROCITOMA III	FRONTAL	FRONTAL	YES	GROSS TOTAL RESECTION (>90
532.00	UCSC Roma	18.00	M	80.00	ASTROC III	FRONTAL	FRONTAL	YES	GROSS TOTAL RESECTION (>90
533.00	UCSC Roma	65.00	M	40.00	OLIGODENDROGLIOMA III		FRONTAL+TEMP(	YES	PARTIAL RESECTION (<90%)
534.00	UCSC Roma	42.00	M	90.00	ASTROC III	FRONTAL	FRONTAL	YES	PARTIAL RESECTION (<90%)
535.00	UCSC Roma	54.00	F	40.00	GBM	TEMPORAL	TEMPORAL	YES	PARTIAL RESECTION (<90%)
536.00	UCSC Roma	67.00	M	100.00	OTHER (ATIPIC MENINGIOMI)	PARIETAL	PARIETAL	YES	GROSS TOTAL RESECTION (>90
537.00	UCSC Roma	59.00	M	50.00	GBM	PARIETAL	PARIETAL	YES	PARTIAL RESECTION (<90%)
538.00	UCSC Roma	61.00	M	40.00	ASTROC III	TEMPORAL	TEMPORAL	YES	PARTIAL RESECTION (<90%)
539.00	UCSC Roma	41.00	M	80.00	OLIGOASTROCITOMA II	FRONTAL	FRONTAL	YES	GROSS TOTAL RESECTION (>90
540.00	UCSC Roma	46.00	F	30.00	GBM	FRONTAL	FRONTAL	YES	PARTIAL RESECTION (<90%)
541.00	UCSC Roma	47.00	F	90.00	METASTASES	FRONTAL	FRONTAL	YES	GROSS TOTAL RESECTION (>90
542.00	UCSC Roma	55.00	F	100.00	ASTROC III		TEMPORAL+OCCI	YES	PARTIAL RESECTION (<90%)
543.00	UCSC Roma	46.00	M	90.00	OLIGOASTROCITOMA III	FRONTAL	FRONTAL	YES	GROSS TOTAL RESECTION (>90

544.00	UCSC Roma	70.00	F	20.00	GBM	FRONTAL	FRONTAL	YES	PARTIAL RESECTION (<90%)
545.00	UCSC Roma	77.00	M	70.00	GBM	FRONTAL	FRONTAL	YES	PARTIAL RESECTION (<90%)
546.00	UCSC Roma	44.00	M	50.00	ASTROC III	PARIETAL	PARIETAL	YES	PARTIAL RESECTION (<90%)
547.00	UCSC Roma	65.00	F	80.00	METASTASES		CEREBELLAR	NO	
548.00	UCSC Roma	75.00	M	50.00	ASTROC III	TEMPORAL	TEMPORAL	YES	PARTIAL RESECTION (<90%)
549.00	UCSC Roma	61.00	F	40.00	GBM	FRONTAL	FRONTAL	YES	PARTIAL RESECTION (<90%)
550.00	UCSC Roma	60.00	M	70.00	OLIGOASTROCITOMA II	FRONTAL	FRONTAL	YES	GROSS TOTAL RESECTION (>9C
551.00	UCSC Roma	28.00	F	80.00	ASTROC III		TEMPORAL+INSU	YES	PARTIAL RESECTION (<90%)
552.00	UCSC Roma	79.00	F	40.00	GBM	FRONTAL	FRONTAL	YES	PARTIAL RESECTION (<90%)
553.00	UCSC Roma	42.00	M	90.00	OLIGODENDROGLIOMA III	FRONTAL	FRONTAL	YES	PARTIAL RESECTION (<90%)
554.00	UCSC Roma	63.00	M	30.00	ASTROC III	FRONTAL	FRONTAL	YES	PARTIAL RESECTION (<90%)
555.00	UCSC Roma	59.00	F	80.00	ASTROC III	FRONTAL	FRONTAL	YES	BIOPSY
556.00	UCSC Roma	33.00	M	90.00	OLIGOASTROCITOMA II	FRONTAL	FRONTAL	YES	PARTIAL RESECTION (<90%)
557.00	UCSC Roma	72.00	M	20.00		BILATERAL	MULTICENTRIC	NO	
558.00	UCSC Roma	23.00	F	80.00	OLIGODENDROGLIOMA III	PARIETAL	PARIETAL	YES	GROSS TOTAL RESECTION (>9C
559.00	UCSC Roma	22.00	M	90.00	OTHER (ATIPIC MENINGIOMI)	MULTILOBULAR	MULTICENTRIC	YES	GROSS TOTAL RESECTION (>9C
560.00	UCSC Roma	54.00	M	60.00	OLIGOASTROCITOMA III	MULTICENTRIC	MULTICENTRIC	YES	BIOPSY
561.00	UCSC Roma	72.00	F	60.00	GBM	TEMPORAL	TEMPORAL	YES	BIOPSY
562.00	UCSC Roma	68.00	F	60.00	GBM	FRONTAL	FRONTAL	YES	GROSS TOTAL RESECTION (>9C
563.00	UCSC Roma	51.00	F	70.00	GBM	TEMPORAL	TEMPORAL	YES	GROSS TOTAL RESECTION (>9C
564.00	UCSC Roma	52.00	F	70.00	GBM	TEMPORAL	TEMPORAL	YES	GROSS TOTAL RESECTION (>9C
565.00	UCSC Roma	59.00	M	70.00		FRONTAL	FRONTAL	NO	
566.00	UCSC Roma	65.00	F	70.00	GBM	FRONTAL	FRONTAL	YES	GROSS TOTAL RESECTION (>9C
567.00	UCSC Roma	39.00	F	70.00	GBM	FRONTAL	FRONTAL	YES	PARTIAL RESECTION (<90%)
568.00	MILANO-BESTA	42.00	M	60.00	GBM	FRONTAL	FRONTAL	YES	PARTIAL RESECTION (<90%)
569.00	MILANO-BESTA	43.00	M	100.00	OLIGOASTROCITOMA II	MULTILOBULAR	MULTICENTRIC	YES	GROSS TOTAL RESECTION (>9C
570.00	MILANO-BESTA	53.00	F	50.00	OLIGOASTROCITOMA III	FRONTAL	FRONTAL	YES	GROSS TOTAL RESECTION (>9C
571.00	MILANO-BESTA	55.00	F	90.00	GBM	FRONTAL	FRONTAL	YES	BIOPSY
572.00	MILANO-BESTA	51.00	M	80.00	GBM	FRONTAL	FRONTAL	YES	PARTIAL RESECTION (<90%)
573.00	MILANO-BESTA	56.00	F	100.00	OLIGOASTROCITOMA III	FRONTAL	FRONTAL	YES	GROSS TOTAL RESECTION (>9C
574.00	MILANO-BESTA	37.00	M	60.00	OLIGOASTROCITOMA III	TEMPORAL	TEMPORAL	YES	PARTIAL RESECTION (<90%)
575.00	MILANO-BESTA	25.00	F	80.00	OLIGOASTROCITOMA III	FRONTAL	FRONTAL	YES	GROSS TOTAL RESECTION (>9C
576.00	MILANO-BESTA	32.00	M	80.00		TEMPORAL	TEMPORAL	YES	GROSS TOTAL RESECTION (>9C
577.00	MILANO-BESTA	39.00	M	100.00	OLIGOASTROCITOMA III	FRONTAL	FRONTAL	YES	GROSS TOTAL RESECTION (>9C
578.00	MILANO-BESTA	50.00	F	60.00	GBM	MULTICENTRIC	MULTICENTRIC	YES	GROSS TOTAL RESECTION (>9C
579.00	MILANO-BESTA	90.00	F	49.00	OLIGOASTROCITOMA II	FRONTAL	FRONTAL	YES	GROSS TOTAL RESECTION (>9C
580.00	MILANO-BESTA	33.00	F	90.00	GBM	TEMPORAL	TEMPORAL	YES	PARTIAL RESECTION (<90%)
581.00	MILANO-BESTA	67.00	M	70.00	GBM	FRONTAL	FRONTAL	YES	GROSS TOTAL RESECTION (>9C
582.00	MILANO-BESTA	34.00	M	80.00	GBM	BILATERAL	MULTICENTRIC	YES	PARTIAL RESECTION (<90%)
583.00	MILANO-BESTA	38.00	M	90.00	OLIGOASTROCITOMA III	FRONTAL	FRONTAL	YES	GROSS TOTAL RESECTION (>9C
584.00	MILANO-BESTA	69.00	M	60.00	ASTROC III	PARIETAL	PARIETAL	YES	
585.00	MILANO-BESTA	55.00	M	60.00	OTHER (ATIPIC MENINGIOMI)	MULTILOBULAR	MULTICENTRIC	YES	PARTIAL RESECTION (<90%)

586.00	MILANO-BESTA	64.00	M	50.00	ASTROC III	TEMPORAL	TEMPORAL	YES	GROSS TOTAL RESECTION (>90
587.00	MILANO-BESTA	49.00	M	60.00	ASTROC III	MULTILOBULAR	MULTICENTRIC	YES	PARTIAL RESECTION (<90%)
588.00	MILANO-BESTA	49.00	M	60.00	GBM	MULTICENTRIC	MULTICENTRIC	YES	GROSS TOTAL RESECTION (>90
589.00	MILANO-BESTA	36.00	M	100.00	GBM	FRONTAL	FRONTAL	YES	GROSS TOTAL RESECTION (>90
590.00	MILANO-BESTA	39.00	M	50.00	GBM	FRONTAL	FRONTAL	YES	GROSS TOTAL RESECTION (>90
591.00	MILANO-BESTA	45.00	M	80.00	GBM	TEMPORAL	TEMPORAL	YES	GROSS TOTAL RESECTION (>90
592.00	MILANO-BESTA	46.00	M	100.00	GBM	TEMPORAL	TEMPORAL	YES	PARTIAL RESECTION (<90%)
593.00	MILANO-BESTA	30.00	F	70.00	GBM	FRONTAL	FRONTAL	YES	GROSS TOTAL RESECTION (>90
594.00	MILANO-BESTA	64.00	M	60.00	GBM	MULTILOBULAR	MULTICENTRIC	YES	GROSS TOTAL RESECTION (>90
595.00	MILANO-BESTA	52.00	M	40.00	ASTRO II	FRONTAL	FRONTAL	YES	GROSS TOTAL RESECTION (>90
596.00	MILANO-BESTA	37.00	M	80.00	OLIGODENDROGLIOMA III	FRONTAL	FRONTAL	YES	GROSS TOTAL RESECTION (>90
597.00	MILANO-BESTA	62.00	F	80.00	GBM	TEMPORAL	TEMPORAL	YES	GROSS TOTAL RESECTION (>90
598.00	MILANO-BESTA	48.00	F	50.00	GBM	MULTILOBULAR	MULTICENTRIC	YES	PARTIAL RESECTION (<90%)
599.00	MILANO-BESTA	63.00	M	50.00	GBM	FRONTAL	FRONTAL	YES	PARTIAL RESECTION (<90%)
600.00	MILANO-BESTA	56.00	F	40.00	METASTASES	MULTICENTRIC	MULTICENTRIC	YES	PARTIAL RESECTION (<90%)
601.00	MILANO-BESTA	71.00	M	90.00	GBM	TEMPORAL	TEMPORAL	YES	GROSS TOTAL RESECTION (>90
602.00	MILANO-BESTA	37.00	M	80.00	GBM	MULTILOBULAR	MULTICENTRIC	YES	GROSS TOTAL RESECTION (>90
603.00	MILANO-BESTA	43.00	M		GBM	MULTILOBULAR	MULTICENTRIC	YES	GROSS TOTAL RESECTION (>90
604.00	MILANO-BESTA	61.00	M	80.00	GBM	FRONTAL	FRONTAL	YES	GROSS TOTAL RESECTION (>90
605.00	MILANO-BESTA	30.00	F	90.00	OLIGOASTROCITOMA II	TEMPORAL	TEMPORAL	YES	GROSS TOTAL RESECTION (>90
606.00	MILANO-BESTA	59.00	M	50.00	GBM	FRONTAL	FRONTAL	YES	GROSS TOTAL RESECTION (>90
607.00	MILANO-BESTA	29.00	M	100.00	OLIGOASTROCITOMA II	FRONTAL	FRONTAL	YES	PARTIAL RESECTION (<90%)
608.00	MILANO-BESTA	71.00	M	60.00	GBM	TEMPORAL	TEMPORAL	YES	GROSS TOTAL RESECTION (>90
609.00	MILANO-BESTA	60.00	F	90.00	GBM	MULTILOBULAR	MULTICENTRIC	YES	GROSS TOTAL RESECTION (>90
610.00	MILANO-BESTA	68.00	M	40.00	OLIGODENDROGLIOMA III	TEMPORAL	TEMPORAL	YES	GROSS TOTAL RESECTION (>90
611.00	MILANO-BESTA	68.00	M	60.00	GBM	MULTILOBULAR	MULTICENTRIC	YES	GROSS TOTAL RESECTION (>90
612.00	MILANO-BESTA	45.00	M	60.00	GBM	TEMPORAL	TEMPORAL	YES	GROSS TOTAL RESECTION (>90
613.00	MILANO-BESTA	61.00	M	50.00	GBM	TEMPORAL	TEMPORAL	YES	GROSS TOTAL RESECTION (>90
614.00	MILANO-BESTA	69.00	M	70.00	GBM	TEMPORAL	TEMPORAL	YES	GROSS TOTAL RESECTION (>90
615.00	MILANO-BESTA	52.00	F	60.00	GBM	MULTILOBULAR	MULTICENTRIC	YES	GROSS TOTAL RESECTION (>90
616.00	MILANO-BESTA	68.00	M	40.00	GBM	MULTICENTRIC	MULTICENTRIC	YES	PARTIAL RESECTION (<90%)
617.00	MILANO-BESTA	37.00	F	90.00	OLIGODENDROGLIOMA II	TEMPORAL	TEMPORAL	YES	GROSS TOTAL RESECTION (>90
618.00	MILANO-BESTA	64.00	M	50.00	METASTASES	TEMPORAL	TEMPORAL	YES	GROSS TOTAL RESECTION (>90
619.00	MILANO-BESTA	30.00	F	100.00	OLIGODENDROGLIOMA II	FRONTAL	FRONTAL	YES	GROSS TOTAL RESECTION (>90
620.00	MILANO-BESTA	52.00	F	100.00	ASTRO II	TEMPORAL	TEMPORAL	YES	PARTIAL RESECTION (<90%)
621.00	MILANO-BESTA	27.00	F	100.00	OLIGODENDROGLIOMA II	FRONTAL	FRONTAL	YES	GROSS TOTAL RESECTION (>90
622.00	MILANO-BESTA	55.00	M	70.00	OLIGOASTROCITOMA III	FRONTAL	FRONTAL	YES	GROSS TOTAL RESECTION (>90
623.00	MILANO-BESTA	71.00	F	60.00	GBM	MULTICENTRIC	MULTICENTRIC	YES	PARTIAL RESECTION (<90%)
624.00	MILANO-BESTA	57.00	M	100.00	GBM	TEMPORAL	TEMPORAL	YES	GROSS TOTAL RESECTION (>90
625.00	MILANO-BESTA	37.00	M	100.00	OLIGODENDROGLIOMA II	FRONTAL	FRONTAL	YES	GROSS TOTAL RESECTION (>90
626.00	MILANO-BESTA	21.00	F	30.00	OTHER (ATIPIC MENINGIOMI)	MULTILOBULAR	MULTICENTRIC	YES	GROSS TOTAL RESECTION (>90
627.00	MILANO-BESTA	23.00	M	100.00	OLIGODENDROGLIOMA II	INSULA	INSULA	YES	GROSS TOTAL RESECTION (>90

628.00	MILANO-BESTA	19.00	F	90.00	OLIGOASTROCITOMA III	MULTILOBULAR	MULTICENTRIC	YES	PARTIAL RESECTION (<90%)
629.00	MILANO-BESTA	58.00	M	30.00	GBM	MULTILOBULAR	MULTICENTRIC	YES	PARTIAL RESECTION (<90%)
630.00	MILANO-BESTA	47.00	F	90.00	OTHER (ATIPIC MENINGIOMI)	FRONTAL	FRONTAL	YES	PARTIAL RESECTION (<90%)
631.00	MILANO-BESTA	59.00	F	60.00	OLIGOASTROCITOMA III	MULTICENTRIC	MULTICENTRIC	YES	PARTIAL RESECTION (<90%)
632.00	MILANO-BESTA	37.00	M	80.00	OLIGOASTROCITOMA III	TEMPORAL	TEMPORAL	YES	PARTIAL RESECTION (<90%)
633.00	MILANO-BESTA	41.00	M	90.00	OLIGOASTROCITOMA III	FRONTAL	FRONTAL	YES	GROSS TOTAL RESECTION (>90
634.00	MILANO-BESTA	47.00	M	100.00	OLIGOASTROCITOMA II	FRONTAL	FRONTAL	YES	GROSS TOTAL RESECTION (>90
635.00	MILANO-BESTA	57.00	F	100.00	OLIGOASTROCITOMA III	FRONTAL	FRONTAL	YES	GROSS TOTAL RESECTION (>90
636.00	MILANO-BESTA	53.00	M	70.00	OTHER (ATIPIC MENINGIOMI)	FRONTAL	FRONTAL	YES	PARTIAL RESECTION (<90%)
637.00	MILANO-BESTA	55.00	F	90.00	OTHER (ATIPIC MENINGIOMI)	MULTILOBULAR	MULTICENTRIC	YES	GROSS TOTAL RESECTION (>90
638.00	MILANO-BESTA	34.00	F	40.00	OLIGOASTROCITOMA III	TEMPORAL	TEMPORAL	YES	GROSS TOTAL RESECTION (>90
639.00	MILANO-BESTA	38.00	M	80.00	OLIGOASTROCITOMA II	FRONTAL	FRONTAL	YES	PARTIAL RESECTION (<90%)
640.00	MILANO-BESTA	63.00	M	100.00	GBM	MULTILOBULAR	MULTICENTRIC	YES	PARTIAL RESECTION (<90%)
641.00	MILANO-BESTA	42.00	M	100.00	OLIGOASTROCITOMA III	MULTILOBULAR	MULTICENTRIC	YES	GROSS TOTAL RESECTION (>90
642.00	MILANO-BESTA	42.00	M	100.00	OLIGOASTROCITOMA II	FRONTAL	FRONTAL	YES	GROSS TOTAL RESECTION (>90
643.00	MILANO-BESTA	65.00	M	90.00	ASTROC III	FRONTAL	FRONTAL	YES	GROSS TOTAL RESECTION (>90
644.00	MILANO-BESTA	67.00	M	90.00	OLIGOASTROCITOMA II	FRONTAL	FRONTAL	YES	PARTIAL RESECTION (<90%)
645.00	MILANO-BESTA	73.00	F	60.00	OTHER (ATIPIC MENINGIOMI)	FRONTAL	FRONTAL	YES	GROSS TOTAL RESECTION (>90
646.00	MILANO-BESTA	56.00	F	40.00	METASTASES	MULTILOBULAR	MULTICENTRIC	YES	PARTIAL RESECTION (<90%)
647.00	MILANO-BESTA	61.00	F	70.00	GBM	MULTILOBULAR	MULTICENTRIC	YES	PARTIAL RESECTION (<90%)
648.00	MILANO-BESTA	37.00	M	90.00	OLIGOASTROCITOMA II	MULTILOBULAR	MULTICENTRIC	YES	PARTIAL RESECTION (<90%)
649.00	MILANO-BESTA	36.00	M	100.00	OLIGOASTROCITOMA II	FRONTAL	FRONTAL	YES	GROSS TOTAL RESECTION (>90
650.00	MILANO-BESTA	36.00	M	100.00	OLIGOASTROCITOMA II	FRONTAL	FRONTAL	YES	GROSS TOTAL RESECTION (>90
651.00	MILANO-BESTA	52.00	M	60.00	GBM	MULTICENTRIC	MULTICENTRIC	YES	PARTIAL RESECTION (<90%)
652.00	MILANO-BESTA	33.00	M	100.00	ASTROC III	FRONTAL	FRONTAL	YES	GROSS TOTAL RESECTION (>90
653.00	MILANO-BESTA	38.00	M	100.00	ASTROC III	FRONTAL	FRONTAL	YES	GROSS TOTAL RESECTION (>90
654.00	MILANO-BESTA	36.00	M	100.00	OLIGOASTROCITOMA II	FRONTAL	FRONTAL	YES	PARTIAL RESECTION (<90%)
655.00	MILANO-BESTA	73.00	F	60.00	GBM	TEMPORAL	TEMPORAL	YES	PARTIAL RESECTION (<90%)
656.00	MILANO-BESTA	22.00	M	60.00	ASTROC III	FRONTAL	FRONTAL	YES	GROSS TOTAL RESECTION (>90
657.00	MILANO-BESTA	74.00	F	70.00	OLIGOASTROCITOMA III	FRONTAL	FRONTAL	YES	GROSS TOTAL RESECTION (>90
658.00	MILANO-BESTA	65.00	F	100.00	METASTASES	TEMPORAL	TEMPORAL	YES	GROSS TOTAL RESECTION (>90
659.00	MILANO-BESTA	41.00	M	100.00	ASTRO II	MULTILOBULAR	MULTICENTRIC	YES	PARTIAL RESECTION (<90%)
660.00	MILANO-BESTA	62.00	F	100.00	OLIGODENDROGLIOMA III	FRONTAL	FRONTAL	YES	GROSS TOTAL RESECTION (>90
661.00	MILANO-BESTA	62.00	F	100.00	ASTRO II	MULTILOBULAR	MULTICENTRIC	YES	PARTIAL RESECTION (<90%)
662.00	MILANO-BESTA	62.00	M	80.00	METASTASES	PARIETAL	PARIETAL	YES	PARTIAL RESECTION (<90%)
663.00	MILANO-BESTA	49.00	M	100.00	OLIGOASTROCITOMA II	MULTILOBULAR	MULTICENTRIC	YES	PARTIAL RESECTION (<90%)
664.00	MILANO-BESTA	67.00	M	60.00	GBM	TEMPORAL	TEMPORAL	YES	PARTIAL RESECTION (<90%)
665.00	MILANO-BESTA	68.00	M	100.00	ASTROC III	MULTILOBULAR	MULTICENTRIC	YES	PARTIAL RESECTION (<90%)
666.00	MILANO-BESTA	41.00	F	100.00	OLIGODENDROGLIOMA II	MULTILOBULAR	MULTICENTRIC	YES	PARTIAL RESECTION (<90%)
667.00	MILANO-BESTA	61.00	M	100.00	ASTRO II	MULTILOBULAR	MULTICENTRIC	YES	PARTIAL RESECTION (<90%)
668.00	MILANO-BESTA	67.00	M	70.00	METASTASES	FRONTAL	FRONTAL	YES	PARTIAL RESECTION (<90%)
669.00	MILANO-BESTA	55.00	F	70.00	ASTROC III	BILATERAL	MULTICENTRIC	YES	BIOPSY

670.00	MILANO-BESTA	56.00	F	80.00	GBM	PARIETAL	PARIETAL	YES	PARTIAL RESECTION (<90%)
671.00	MILANO-BESTA	57.00	M	60.00	OTHER (ATIPIC MENINGIOMI)	TEMPORAL	TEMPORAL	YES	PARTIAL RESECTION (<90%)
672.00	MILANO-BESTA	57.00	M	100.00	ASTROC III	MULTICENTRIC	MULTICENTRIC	YES	PARTIAL RESECTION (<90%)
673.00	MILANO-BESTA	41.00	F	100.00	GBM	FRONTAL	FRONTAL	YES	GROSS TOTAL RESECTION (>90
674.00	MILANO-BESTA	69.00	F	100.00	METASTASES	FRONTAL	FRONTAL	YES	GROSS TOTAL RESECTION (>90
675.00	MILANO-BESTA	27.00	M	90.00	ASTRO II	FRONTAL	FRONTAL	YES	GROSS TOTAL RESECTION (>90
676.00	MILANO-BESTA	69.00	M	40.00	GBM	MULTILOBULAR	MULTICENTRIC	YES	PARTIAL RESECTION (<90%)
677.00	MILANO-BESTA	34.00	F	90.00	OLIGODENDROGLIOMA II	FRONTAL	FRONTAL	YES	BIOPSY
678.00	MILANO-BESTA	65.00	M	80.00	GBM	MULTILOBULAR	MULTICENTRIC	YES	PARTIAL RESECTION (<90%)
679.00	MILANO-BESTA	57.00	F	40.00	METASTASES	MULTICENTRIC	MULTICENTRIC	YES	GROSS TOTAL RESECTION (>90
680.00	MILANO-BESTA	51.00	F	50.00	OLIGOASTROCITOMA III	TEMPORAL	TEMPORAL	YES	GROSS TOTAL RESECTION (>90
681.00	MILANO-BESTA	63.00	M	40.00	OTHER (ATIPIC MENINGIOMI)	MULTICENTRIC	MULTICENTRIC	YES	PARTIAL RESECTION (<90%)
682.00	MILANO-BESTA	76.00	M	100.00	OLIGOASTROCITOMA II	TEMPORAL	TEMPORAL	YES	GROSS TOTAL RESECTION (>90
683.00	MILANO-BESTA	36.00	F	100.00	OLIGOASTROCITOMA II	BILATERAL	MULTICENTRIC	YES	PARTIAL RESECTION (<90%)
684.00	MILANO-BESTA	55.00	M	100.00	GBM	TEMPORAL	TEMPORAL	YES	PARTIAL RESECTION (<90%)
685.00	MILANO-BESTA	66.00	F	40.00	GBM	TEMPORAL	TEMPORAL	YES	PARTIAL RESECTION (<90%)
686.00	MILANO-BESTA	39.00	M	80.00	GBM	FRONTAL	FRONTAL	YES	PARTIAL RESECTION (<90%)
687.00	MILANO-BESTA	49.00	M	70.00	OLIGOASTROCITOMA III	FRONTAL	FRONTAL	YES	PARTIAL RESECTION (<90%)
688.00	MILANO-BESTA	76.00	M	60.00	GBM	FRONTAL	FRONTAL	YES	PARTIAL RESECTION (<90%)
689.00	MILANO-BESTA	76.00	M	90.00	OLIGODENDROGLIOMA III	TEMPORAL	TEMPORAL	YES	GROSS TOTAL RESECTION (>90
690.00	MILANO-BESTA	42.00	M	100.00	OLIGOASTROCITOMA III	FRONTAL	FRONTAL	YES	PARTIAL RESECTION (<90%)
691.00	MILANO-BESTA	57.00	F	80.00	GBM	MULTILOBULAR	MULTICENTRIC	YES	PARTIAL RESECTION (<90%)
692.00	MILANO-BESTA	42.00	M	100.00		FRONTAL	FRONTAL	YES	GROSS TOTAL RESECTION (>90
693.00	MILANO-BESTA	63.00	F	60.00	OTHER (ATIPIC MENINGIOMI)	TEMPORAL	TEMPORAL	YES	GROSS TOTAL RESECTION (>90
694.00	MILANO-BESTA	71.00	F	70.00	GBM	FRONTAL	FRONTAL	YES	GROSS TOTAL RESECTION (>90
695.00	MILANO-BESTA	47.00	M	90.00	GBM	BILATERAL	MULTICENTRIC	YES	PARTIAL RESECTION (<90%)
696.00	MILANO-BESTA	53.00	M	100.00	GBM	FRONTAL	FRONTAL	YES	GROSS TOTAL RESECTION (>90
697.00	MILANO-BESTA	49.00	M	60.00	GBM	MULTILOBULAR	MULTICENTRIC	YES	GROSS TOTAL RESECTION (>90
698.00	MILANO-BESTA	70.00	F	100.00	GBM	TEMPORAL	TEMPORAL	YES	GROSS TOTAL RESECTION (>90
699.00	MILANO-BESTA	40.00	F	90.00	ASTROC III	MULTILOBULAR	MULTICENTRIC	YES	PARTIAL RESECTION (<90%)
700.00	MILANO-BESTA	41.00	F	70.00	OLIGOASTROCITOMA III	MULTILOBULAR	MULTICENTRIC	YES	GROSS TOTAL RESECTION (>90
701.00	MILANO-BESTA	73.00	F	100.00	GBM	TEMPORAL	TEMPORAL	YES	GROSS TOTAL RESECTION (>90
702.00	MILANO-BESTA	58.00	M	70.00	GBM	FRONTAL	FRONTAL	YES	PARTIAL RESECTION (<90%)
703.00	MILANO-BESTA	23.00	M	100.00	OLIGODENDROGLIOMA II	TEMPORAL	TEMPORAL	YES	GROSS TOTAL RESECTION (>90
704.00	MILANO-BESTA	60.00	F	70.00	GBM	TEMPORAL	TEMPORAL	YES	PARTIAL RESECTION (<90%)
705.00	MILANO-BESTA	48.00	M	60.00	GBM	FRONTAL	FRONTAL	YES	PARTIAL RESECTION (<90%)
706.00	MILANO-BESTA	55.00	M	50.00	GBM	FRONTAL	FRONTAL	YES	PARTIAL RESECTION (<90%)
707.00	MILANO-BESTA	69.00	M	100.00	OLIGODENDROGLIOMA II	TEMPORAL	TEMPORAL	YES	GROSS TOTAL RESECTION (>90
708.00	MILANO-BESTA	49.00	M	60.00	GBM	BILATERAL	MULTICENTRIC	YES	GROSS TOTAL RESECTION (>90
709.00	MILANO-BESTA	66.00	F	70.00	GBM	TEMPORAL	TEMPORAL	YES	GROSS TOTAL RESECTION (>90
710.00	MILANO-BESTA	67.00	M	90.00	GBM	FRONTAL	FRONTAL	YES	PARTIAL RESECTION (<90%)
711.00	MILANO-BESTA	45.00	M	70.00	GBM	MULTICENTRIC	MULTICENTRIC	YES	PARTIAL RESECTION (<90%)

712.00	MILANO-BESTA	53.00	F	100.00	OLIGODENDROGLIOMA II	FRONTAL	FRONTAL	YES	GROSS TOTAL RESECTION (>90
713.00	MILANO-BESTA	59.00	F	50.00	GBM	MULTILOBULAR	MULTICENTRIC	YES	PARTIAL RESECTION (<90%)
714.00	MILANO-BESTA	38.00	M	100.00	OLIGOASTROCITOMA II	FRONTAL	FRONTAL	YES	PARTIAL RESECTION (<90%)
715.00	MILANO-BESTA	51.00	M	100.00	OLIGOASTROCITOMA II	MULTILOBULAR	MULTICENTRIC	YES	GROSS TOTAL RESECTION (>90
716.00	MILANO-BESTA	76.00	M	40.00	OTHER (ATIPIC MENINGIOMI)	MULTICENTRIC	MULTICENTRIC	YES	GROSS TOTAL RESECTION (>90
717.00	MILANO-BESTA	57.00	F	80.00	GBM	TEMPORAL	TEMPORAL	YES	GROSS TOTAL RESECTION (>90
718.00	MILANO-BESTA	44.00	M	70.00	OTHER (ATIPIC MENINGIOMI)	MULTILOBULAR	MULTICENTRIC	YES	PARTIAL RESECTION (<90%)
719.00	MILANO-BESTA	41.00	F	100.00	OLIGOASTROCITOMA II	MULTILOBULAR	MULTICENTRIC	YES	PARTIAL RESECTION (<90%)
720.00	MILANO-BESTA	39.00	F	100.00	METASTASES	MULTILOBULAR	MULTICENTRIC	YES	GROSS TOTAL RESECTION (>90
721.00	MILANO-BESTA	68.00	M	90.00	OLIGOASTROCITOMA II	MULTILOBULAR	MULTICENTRIC	YES	PARTIAL RESECTION (<90%)
722.00	MILANO-BESTA	22.00	M	100.00	OLIGOASTROCITOMA III	MULTILOBULAR	MULTICENTRIC	YES	PARTIAL RESECTION (<90%)
723.00	MILANO-BESTA	38.00	F	90.00	GBM	MULTILOBULAR	MULTICENTRIC	YES	PARTIAL RESECTION (<90%)
724.00	MILANO-BESTA	55.00	M	60.00	GBM	MULTILOBULAR	MULTICENTRIC	YES	PARTIAL RESECTION (<90%)
725.00	MILANO-BESTA	44.00	F	80.00	GBM	PARIETAL	PARIETAL	YES	GROSS TOTAL RESECTION (>90
726.00	MILANO-BESTA	49.00	M	90.00	ASTROC III	BILATERAL	MULTICENTRIC	YES	PARTIAL RESECTION (<90%)
727.00	MILANO-BESTA	34.00	F	100.00	OLIGOASTROCITOMA II	MULTILOBULAR	MULTICENTRIC	YES	GROSS TOTAL RESECTION (>90
728.00	MILANO-BESTA	35.00	M	100.00	OLIGOASTROCITOMA II	PARIETAL	PARIETAL	YES	GROSS TOTAL RESECTION (>90
729.00	MILANO-BESTA	72.00	M	100.00	GBM	MULTICENTRIC	MULTICENTRIC	YES	GROSS TOTAL RESECTION (>90
730.00	MILANO-BESTA	56.00	F	80.00	OLIGOASTROCITOMA II	MULTILOBULAR	MULTICENTRIC	YES	GROSS TOTAL RESECTION (>90
731.00	MILANO-BESTA	59.00	M	100.00	GBM	TEMPORAL	TEMPORAL	YES	GROSS TOTAL RESECTION (>90
732.00	MILANO-BESTA	52.00	M	70.00	GBM	FRONTAL	FRONTAL	YES	GROSS TOTAL RESECTION (>90
733.00	MILANO-BESTA	76.00	M	70.00	GBM	MULTILOBULAR	MULTICENTRIC	YES	GROSS TOTAL RESECTION (>90
734.00	MILANO-BESTA	39.00	F	50.00	ASTROC III	MULTILOBULAR	MULTICENTRIC	YES	PARTIAL RESECTION (<90%)
735.00	MILANO-BESTA	21.00	M	80.00	GBM	FRONTAL	FRONTAL	YES	GROSS TOTAL RESECTION (>90
736.00	MILANO-BESTA	39.00	F	100.00	ASTROC III	INSULA	INSULA	YES	GROSS TOTAL RESECTION (>90
737.00	MILANO-BESTA	69.00	F	70.00	OLIGODENDROGLIOMA II	FRONTAL	FRONTAL	YES	PARTIAL RESECTION (<90%)
738.00	MILANO-BESTA	40.00	F		OLIGOASTROCITOMA III	FRONTAL	FRONTAL	YES	GROSS TOTAL RESECTION (>90
739.00	MILANO-BESTA	60.00	M	80.00	OLIGODENDROGLIOMA II	MULTILOBULAR	MULTICENTRIC	YES	GROSS TOTAL RESECTION (>90
740.00	MILANO-BESTA	73.00	M	90.00	GBM	MULTILOBULAR	MULTICENTRIC	YES	GROSS TOTAL RESECTION (>90
741.00	MILANO-BESTA	64.00	M	80.00	METASTASES	FRONTAL	FRONTAL	YES	GROSS TOTAL RESECTION (>90
742.00	MILANO-BESTA	59.00	M	100.00	METASTASES	FRONTAL	FRONTAL	YES	GROSS TOTAL RESECTION (>90
743.00	MILANO-BESTA	63.00	F	60.00	GBM	FRONTAL	FRONTAL	YES	PARTIAL RESECTION (<90%)
744.00	MILANO-BESTA	65.00	M	100.00	OLIGOASTROCITOMA III	PARIETAL	PARIETAL	YES	GROSS TOTAL RESECTION (>90
745.00	MILANO-BESTA	45.00	F	50.00	OLIGOASTROCITOMA III	MULTILOBULAR	MULTICENTRIC	YES	PARTIAL RESECTION (<90%)
746.00	MILANO-BESTA	51.00	M	100.00	GBM	FRONTAL	FRONTAL	YES	PARTIAL RESECTION (<90%)
747.00	MILANO-BESTA	54.00	M	70.00	GBM	PARIETAL	PARIETAL	YES	PARTIAL RESECTION (<90%)
748.00	MILANO-BESTA	42.00	M	80.00	GBM	FRONTAL	FRONTAL	YES	GROSS TOTAL RESECTION (>90
749.00	MILANO-BESTA	69.00	M	60.00	GBM	FRONTAL	FRONTAL	YES	PARTIAL RESECTION (<90%)
750.00	MILANO-BESTA	50.00	M	30.00	GBM	FRONTAL	FRONTAL	YES	PARTIAL RESECTION (<90%)
751.00	MILANO-BESTA	62.00	F	90.00	GBM	PARIETAL	PARIETAL	YES	GROSS TOTAL RESECTION (>90
752.00	MILANO-BESTA	32.00	M	100.00	METASTASES	MULTICENTRIC	MULTICENTRIC	YES	GROSS TOTAL RESECTION (>90
753.00	MILANO-BESTA	58.00	M	80.00	GBM	FRONTAL	FRONTAL	YES	GROSS TOTAL RESECTION (>90

754.00	MILANO-BESTA	70.00	M	100.00	GBM	OCCIPITAL	OCCIPITAL	YES	GROSS TOTAL RESECTION (>90
755.00	MILANO-BESTA	46.00	M	70.00	ASTROC III	BILATERAL	MULTICENTRIC	YES	BIOPSY
756.00	MILANO-BESTA	50.00	M	90.00	GBM	TEMPORAL	TEMPORAL	YES	GROSS TOTAL RESECTION (>90
757.00	MILANO-BESTA	63.00	M	90.00	METASTASES	TEMPORAL	TEMPORAL	YES	GROSS TOTAL RESECTION (>90
758.00	MILANO-BESTA	42.00	F	100.00	OLIGOASTROCITOMA III	FRONTAL	FRONTAL	YES	GROSS TOTAL RESECTION (>90
759.00	MILANO-BESTA	59.00	M	50.00	GBM	MULTILOBULAR	MULTICENTRIC	YES	GROSS TOTAL RESECTION (>90
760.00	MILANO-BESTA	70.00	M	80.00	GBM	TEMPORAL	TEMPORAL	YES	GROSS TOTAL RESECTION (>90
761.00	MILANO-BESTA	69.00	F	70.00	GBM	MULTILOBULAR	MULTICENTRIC	YES	GROSS TOTAL RESECTION (>90
762.00	MILANO-BESTA	52.00	M	60.00	GBM	MULTILOBULAR	MULTICENTRIC	YES	GROSS TOTAL RESECTION (>90
763.00	MILANO-BESTA	75.00	F	40.00	GBM	FRONTAL	FRONTAL	YES	GROSS TOTAL RESECTION (>90
764.00	MILANO-BESTA	61.00	F	90.00	GBM	MULTILOBULAR	MULTICENTRIC	YES	GROSS TOTAL RESECTION (>90
765.00	MILANO-BESTA	72.00	M	80.00	GBM	FRONTAL	FRONTAL	YES	GROSS TOTAL RESECTION (>90
766.00	MILANO-BESTA	55.00	M	100.00	GBM	TEMPORAL	TEMPORAL	YES	GROSS TOTAL RESECTION (>90
767.00	MILANO-BESTA	73.00	F	90.00	GBM	TEMPORAL	TEMPORAL	YES	GROSS TOTAL RESECTION (>90
768.00	MILANO-BESTA	45.00	M	80.00	GBM	MULTILOBULAR	MULTICENTRIC	YES	GROSS TOTAL RESECTION (>90
769.00	MILANO-BESTA	61.00	F	80.00	METASTASES	FRONTAL	FRONTAL	YES	GROSS TOTAL RESECTION (>90
770.00	MILANO-BESTA	35.00	F	100.00	OTHER (ATIPIC MENINGIOMI)	FRONTAL	FRONTAL	YES	GROSS TOTAL RESECTION (>90
771.00	MILANO-BESTA	56.00	M	70.00	GBM	MULTILOBULAR	MULTICENTRIC	YES	GROSS TOTAL RESECTION (>90
772.00	MILANO-BESTA	61.00	F	80.00	GBM	FRONTAL	FRONTAL	YES	GROSS TOTAL RESECTION (>90
773.00	MILANO-BESTA	36.00	M	90.00	OLIGOASTROCITOMA II	FRONTAL	FRONTAL	YES	GROSS TOTAL RESECTION (>90
774.00	MILANO-BESTA	56.00	F	70.00	METASTASES	MULTILOBULAR	MULTICENTRIC	YES	GROSS TOTAL RESECTION (>90
775.00	MILANO-BESTA	63.00	F	60.00	GBM	TEMPORAL	TEMPORAL	YES	GROSS TOTAL RESECTION (>90
776.00	MILANO-BESTA	73.00	M	60.00	GBM	MULTILOBULAR	MULTICENTRIC	YES	GROSS TOTAL RESECTION (>90
777.00	MILANO-BESTA	57.00	F	30.00	GBM	FRONTAL	FRONTAL	YES	GROSS TOTAL RESECTION (>90
778.00	MILANO-BESTA	58.00	M	80.00	METASTASES	PARIETAL	PARIETAL	YES	GROSS TOTAL RESECTION (>90
779.00	MILANO-BESTA	52.00	M	60.00	GBM	FRONTAL	FRONTAL	YES	GROSS TOTAL RESECTION (>90
780.00	MILANO-BESTA	60.00	M	70.00	GBM	MULTILOBULAR	MULTICENTRIC	YES	GROSS TOTAL RESECTION (>90
781.00	MILANO-BESTA	73.00	M	60.00	GBM	FRONTAL	FRONTAL	YES	GROSS TOTAL RESECTION (>90
782.00	MILANO-BESTA	31.00	F	100.00	OLIGOASTROCITOMA III	FRONTAL	FRONTAL	YES	GROSS TOTAL RESECTION (>90
783.00	MILANO-BESTA	34.00	F	70.00	ASTROC III	TEMPORAL	TEMPORAL	YES	GROSS TOTAL RESECTION (>90
784.00	MILANO-BESTA	37.00	M	100.00	OLIGODENDROGLIOMA III	FRONTAL	FRONTAL	YES	GROSS TOTAL RESECTION (>90
785.00	MILANO-BESTA	57.00	F	90.00	ASTRO II	MULTICENTRIC	MULTICENTRIC	YES	BIOPSY
786.00	MILANO-BESTA	64.00	M	90.00	GBM	TEMPORAL	TEMPORAL	YES	GROSS TOTAL RESECTION (>90
787.00	MILANO-BESTA	53.00	M	70.00	OLIGOASTROCITOMA III	MULTILOBULAR	MULTICENTRIC	YES	GROSS TOTAL RESECTION (>90
788.00	MILANO-BESTA	65.00	M	80.00	GBM	MULTICENTRIC	MULTICENTRIC	YES	PARTIAL RESECTION (<90%)
789.00	MILANO-BESTA	66.00	M	70.00	OLIGOASTROCITOMA III	MULTILOBULAR	MULTICENTRIC	YES	GROSS TOTAL RESECTION (>90
790.00	MILANO-BESTA	71.00	M	80.00	GBM	FRONTAL	FRONTAL	YES	GROSS TOTAL RESECTION (>90
791.00	MILANO-BESTA	62.00	M	100.00	GBM	TEMPORAL	TEMPORAL	YES	GROSS TOTAL RESECTION (>90
792.00	TRIESTE	67.00	F	100.00	GBM	MULTILOBULAR	MULTICENTRIC	YES	PARTIAL RESECTION (<90%)
793.00	TRIESTE	71.00	M	90.00	GBM	MULTILOBULAR	MULTICENTRIC	YES	PARTIAL RESECTION (<90%)
794.00	TRIESTE	28.00	M	100.00	ASTRO II	FRONTAL	FRONTAL	YES	GROSS TOTAL RESECTION (>90
795.00	LECCO	39.00	M	80.00	GBM	TEMPORAL	TEMPORAL	YES	GROSS TOTAL RESECTION (>90

796.00	LECCO	52.00	F	70.00	GBM	PARIETAL	PARIETAL	YES	PARTIAL RESECTION (<90%)
797.00	LECCO	69.00	F	60.00	METASTASES	FRONTAL	FRONTAL	YES	GROSS TOTAL RESECTION (>90
798.00	LECCO	61.00	F	70.00	GBM	FRONTAL	FRONTAL	YES	PARTIAL RESECTION (<90%)
799.00	LECCO	71.00	M	70.00	GBM	PARIETAL	PARIETAL	YES	GROSS TOTAL RESECTION (>90
800.00	LECCO	67.00	M	80.00	OTHER (ATIPIC MENINGIOMI)	PARIETAL	PARIETAL	YES	GROSS TOTAL RESECTION (>90
801.00	LECCO	61.00	M	70.00	ASTRO II	TEMPORAL	TEMPORAL	YES	GROSS TOTAL RESECTION (>90
802.00	LECCO	51.00	F	80.00	ASTRO II	FRONTAL	FRONTAL	YES	GROSS TOTAL RESECTION (>90
803.00	LECCO	58.00	F	90.00	METASTASES	FRONTAL	FRONTAL	YES	GROSS TOTAL RESECTION (>90
804.00	LECCO	53.00	M	70.00	GBM	PARIETAL	PARIETAL	YES	PARTIAL RESECTION (<90%)
805.00	LECCO	63.00	F	80.00	GBM	FRONTAL	FRONTAL	YES	PARTIAL RESECTION (<90%)
806.00	LECCO	59.00	M	70.00	GBM	TEMPORAL	TEMPORAL	YES	PARTIAL RESECTION (<90%)
807.00	LECCO	71.00	M	60.00	GBM	TEMPORAL	TEMPORAL	YES	GROSS TOTAL RESECTION (>90
808.00	LECCO	69.00	M	80.00	GBM	TEMPORAL	TEMPORAL	YES	GROSS TOTAL RESECTION (>90

Chemotherapy1	Chemotherapy2	Chemotherapy3	Chemotherapy1_type	Chemotherapy2_type	Chemotherapy3_type	RDT1
NO						YES
YES			TEMOZOLOMIDE			YES
NO						YES
NO						YES
YES			TEMOZOLOMIDE			YES
NO						YES
NO						YES
YES			TEMOZOLOMIDE			YES
NO						YES
NO						YES
NO						NO
NO						YES
NO						NO
NO						NO
NO						NO
NO						NO
NO						NO
NO						NO
NO						YES
YES			TEMOZOLOMIDE			YES
NO						NO
NO						NO
NO						NO
NO						NO
NO						NO
NO						YES
NO						NO
NO						NO
NO						NO
NO						NO
YES			TEMOZOLOMIDE			YES
NO						NO
NO						NO
NO						NO
NO						NO
NO						NO
NO						NO
NO						NO
YES			PVC			YES

YES			TEMOZOLOMIDE			YES
YES			TEMOZOLOMIDE			YES
NO						NO
YES			OTHER			NO
NO						NO
YES						YES
NO						YES
NO						NO
NO						NO
YES			TEMOZOLOMIDE			YES
NO						NO
YES			OTHER			YES
YES			OTHER			NO
YES			OTHER			YES
NO						NO
NO						NO
NO						NO
YES			OTHER			YES
YES			TEMOZOLOMIDE			NO
YES			TEMOZOLOMIDE			YES
YES			TEMOZOLOMIDE			NO
YES			TEMOZOLOMIDE			YES
YES			TEMOZOLOMIDE			YES
YES			OTHER			YES
YES			TEMOZOLOMIDE			YES
NO						YES
YES			OTHER			YES
YES	YES		TEMOZOLOMIDE	OTHER		YES
YES			OTHER			YES
NO						NO
YES			TEMOZOLOMIDE			YES
YES			TEMOZOLOMIDE			YES
NO						NO
NO						NO
NO						NO
YES	YES	YES	TEMOZOLOMIDE	OTHER	PVC	YES
YES			OTHER			YES
YES	YES	YES	TEMOZOLOMIDE	FOTEMUSTINE	OTHER	YES
YES			OTHER			NO
NO						NO
YES			OTHER			YES

YES		OTHER		YES
YES		OTHER		YES
YES		TEMOZOLOMIDE		YES
NO				NO
YES	YES	TEMOZOLOMIDE	OTHER	YES
YES		TEMOZOLOMIDE		YES
YES		TEMOZOLOMIDE		YES
NO				YES
YES		OTHER		NO
YES		TEMOZOLOMIDE		YES
YES		TEMOZOLOMIDE		YES
NO				YES
YES		TEMOZOLOMIDE		NO
YES		TEMOZOLOMIDE		YES
YES		OTHER		NO
NO				NO
NO				NO
YES		TEMOZOLOMIDE		YES
NO				NO
NO				YES
NO				NO
YES		TEMOZOLOMIDE		NO
YES		TEMOZOLOMIDE		YES
NO				NO
YES		TEMOZOLOMIDE		YES
YES		OTHER		YES
YES		TEMOZOLOMIDE		YES
NO				NO
YES				YES
YES				NO
YES				NO
YES		TEMOZOLOMIDE		NO
YES				YES
NO				NO
NO				NO
NO				NO
YES				NO
YES		TEMOZOLOMIDE		NO
YES		TEMOZOLOMIDE		NO
NO				NO
YES		TEMOZOLOMIDE		YES
YES	YES	TEMOZOLOMIDE	GLIADEL	YES

YES		OTHER		YES
YES		TEMOZOLOMIDE		YES
NO				YES
NO				NO
YES		TEMOZOLOMIDE		YES
NO				NO
YES		TEMOZOLOMIDE		YES
NO				NO
YES		TEMOZOLOMIDE		YES
YES		OTHER		YES
NO				YES
NO				YES
YES		TEMOZOLOMIDE		YES
NO				NO
YES		TEMOZOLOMIDE		YES
YES		TEMOZOLOMIDE		YES
NO				NO
NO				NO
YES		TEMOZOLOMIDE		YES
YES		TEMOZOLOMIDE		YES
YES		TEMOZOLOMIDE		NO
YES	YES	TEMOZOLOMIDE	FOTEMUSTINE	YES
NO				NO
YES		TEMOZOLOMIDE		YES
NO				NO
NO				YES
YES	YES	TEMOZOLOMIDE	BEVACIZUMAB	YES
YES		TEMOZOLOMIDE		YES
YES		TEMOZOLOMIDE		YES
YES		TEMOZOLOMIDE		YES
YES		TEMOZOLOMIDE		YES
YES		TEMOZOLOMIDE		YES
YES		TEMOZOLOMIDE		YES
YES		TEMOZOLOMIDE		YES
YES		OTHER		YES
YES		TEMOZOLOMIDE		YES
YES		TEMOZOLOMIDE		YES
YES		TEMOZOLOMIDE		YES
YES		TEMOZOLOMIDE		YES
YES		TEMOZOLOMIDE		YES
NO				NO
NO				YES

YES		TEMOZOLOMIDE		YES
YES		TEMOZOLOMIDE		YES
NO				YES
YES		OTHER		YES
YES		TEMOZOLOMIDE		YES
NO				NO
NO				YES
YES		TEMOZOLOMIDE		YES
NO				NO
YES		TEMOZOLOMIDE		YES
YES		TEMOZOLOMIDE		YES
YES		OTHER		NO
NO				NO
NO				YES
YES				YES
YES				YES
YES		TEMOZOLOMIDE		YES
NO				YES
YES				YES
NO				NO
YES				YES
NO				NO
YES		TEMOZOLOMIDE		NO
YES		BEVACIZUMAB		YES
YES		OTHER		YES
NO				NO
NO				NO
NO				NO
YES		OTHER		NO
NO				NO
YES	YES	TEMOZOLOMIDE	TEMOZOLOMIDE	YES
YES		TEMOZOLOMIDE		NO
NO				NO
NO				NO
NO				NO
NO				NO
YES		TEMOZOLOMIDE		YES
YES		TEMOZOLOMIDE		YES

NO		NO
YES	TEMOZOLOMIDE	YES
YES	TEMOZOLOMIDE	YES
YES	TEMOZOLOMIDE	YES
YES	TEMOZOLOMIDE	NO
NO		NO
NO		NO
NO		NO
YES	TEMOZOLOMIDE	YES
NO		NO
YES	TEMOZOLOMIDE	YES
NO		NO
YES	TEMOZOLOMIDE	YES
YES	TEMOZOLOMIDE	YES
NO		NO
YES	TEMOZOLOMIDE	YES
NO		YES
YES	TEMOZOLOMIDE	YES
NO		NO
NO		NO
NO		NO
NO		NO
YES	TEMOZOLOMIDE	NO
YES	TEMOZOLOMIDE	YES
YES	PVC	YES
YES	TEMOZOLOMIDE	YES
NO		YES
NO		NO
NO		NO
NO		NO
NO		NO
NO		NO
YES	TEMOZOLOMIDE	YES
NO		NO
YES	TEMOZOLOMIDE	YES
NO		NO
YES	TEMOZOLOMIDE	NO
NO		NO
NO		NO
NO		NO
NO		YES
NO		NO

NO				NO
YES		TEMOZOLOMIDE		YES
YES	YES	TEMOZOLOMIDE	TEMOZOLOMIDE	YES
YES	YES	FOTEMUSTINE	TEMOZOLOMIDE	YES
YES		TEMOZOLOMIDE		YES
YES	YES	TEMOZOLOMIDE	PVC	YES
YES		TEMOZOLOMIDE		NO
YES	YES	TEMOZOLOMIDE	TEMOZOLOMIDE	NO
YES		TEMOZOLOMIDE		YES
YES		TEMOZOLOMIDE		YES
YES	YES	TEMOZOLOMIDE	TEMOZOLOMIDE	YES
NO				NO
NO				NO
NO				NO
NO				NO
YES	YES	TEMOZOLOMIDE	TEMOZOLOMIDE	YES
NO				NO
YES		TEMOZOLOMIDE		YES
NO				YES
YES		TEMOZOLOMIDE		NO
YES		TEMOZOLOMIDE		YES
NO				YES
YES		OTHER		YES
YES		TEMOZOLOMIDE		YES
NO				YES
YES		TEMOZOLOMIDE		YES
NO				YES
NO				YES
YES		TEMOZOLOMIDE		NO
NO				YES
NO				NO
YES		TEMOZOLOMIDE		YES
YES		OTHER		NO
YES		OTHER		YES
YES		OTHER		YES
YES		TEMOZOLOMIDE		YES
YES		TEMOZOLOMIDE		YES
YES		OTHER		YES
NO				YES
NO				NO
YES		OTHER		YES
YES		OTHER		YES

YES	OTHER	NO
YES	OTHER	YES
NO		NO
NO		NO
NO		YES
YES	OTHER	NO
YES	OTHER	NO
YES	TEMOZOLOMIDE	YES
NO		YES
YES	TEMOZOLOMIDE	NO
NO		NO
NO		YES
NO		NO
NO		YES
NO		NO
YES	TEMOZOLOMIDE	YES
YES	TEMOZOLOMIDE	YES
YES	TEMOZOLOMIDE	NO
NO		NO
NO		NO
NO		NO
YES	TEMOZOLOMIDE	YES
YES	TEMOZOLOMIDE	YES
YES	TEMOZOLOMIDE	YES
YES	TEMOZOLOMIDE	YES
NO		NO
NO	TEMOZOLOMIDE	NO
YES	TEMOZOLOMIDE	YES
YES	TEMOZOLOMIDE	NO
YES	TEMOZOLOMIDE	YES
NO		NO
NO		YES
YES	TEMOZOLOMIDE	NO
YES	TEMOZOLOMIDE	YES
YES	TEMOZOLOMIDE	YES
NO		NO
NO		NO
YES	TEMOZOLOMIDE	YES
YES	TEMOZOLOMIDE	YES
YES	OTHER	NO
YES	OTHER	NO

YES		OTHER		NO
YES	YES	OTHER	TEMOZOLOMIDE	YES
YES				YES
NO				NO
NO				NO
NO				YES
				YES
NO				NO
NO				NO
NO				YES
NO				NO
YES				YES
NO				YES
NO				NO
YES		OTHER		YES
NO				YES
NO				YES
NO				NO
NO				NO
YES		TEMOZOLOMIDE		YES
YES		TEMOZOLOMIDE		YES
NO				YES
YES		TEMOZOLOMIDE		NO
NO				NO
NO				NO
NO				NO
NO				NO
YES		TEMOZOLOMIDE		YES
YES		TEMOZOLOMIDE		YES
YES		TEMOZOLOMIDE		YES
YES		TEMOZOLOMIDE		YES
NO				NO
YES		TEMOZOLOMIDE		YES
YES		TEMOZOLOMIDE		YES
YES		TEMOZOLOMIDE		YES
YES		TEMOZOLOMIDE		YES
YES		TEMOZOLOMIDE		YES
YES		TEMOZOLOMIDE		NO
YES		TEMOZOLOMIDE		YES
YES		TEMOZOLOMIDE		YES
YES		TEMOZOLOMIDE		NO
YES		TEMOZOLOMIDE		YES

NO				NO
NO				NO
YES		TEMOZOLOMIDE		YES
YES		TEMOZOLOMIDE		YES
YES	YES	TEMOZOLOMIDE	GLIADEL	YES
YES		TEMOZOLOMIDE		YES
YES		TEMOZOLOMIDE		YES
YES	YES	TEMOZOLOMIDE	GLIADEL	YES
YES		TEMOZOLOMIDE		YES
YES		TEMOZOLOMIDE		YES
YES		TEMOZOLOMIDE		YES
YES		TEMOZOLOMIDE		YES
YES		TEMOZOLOMIDE		YES
YES		TEMOZOLOMIDE		YES
YES	YES	TEMOZOLOMIDE	GLIADEL	YES
YES		TEMOZOLOMIDE		YES
YES		TEMOZOLOMIDE		YES
YES	YES	TEMOZOLOMIDE	GLIADEL	YES
YES		TEMOZOLOMIDE		YES
YES		TEMOZOLOMIDE		YES
YES		TEMOZOLOMIDE		YES
YES		TEMOZOLOMIDE		YES
YES		TEMOZOLOMIDE		YES
YES		TEMOZOLOMIDE		YES
YES		TEMOZOLOMIDE		YES
YES		TEMOZOLOMIDE		YES
YES		TEMOZOLOMIDE		YES
YES		TEMOZOLOMIDE		YES
YES		TEMOZOLOMIDE		YES
YES	YES	TEMOZOLOMIDE	PVC	YES
YES		TEMOZOLOMIDE		YES
YES	YES	TEMOZOLOMIDE	FOTEMUSTINE	YES
YES	YES	TEMOZOLOMIDE	GLIADEL	YES
YES	YES	TEMOZOLOMIDE	GLIADEL	YES
YES	YES	TEMOZOLOMIDE	GLIADEL	YES
YES	YES	TEMOZOLOMIDE	GLIADEL	YES
YES		TEMOZOLOMIDE		YES
NO				NO
NO				NO
NO				NO
NO				NO
NO				NO

NO		NO
NO		NO
NO		NO
YES		YES
YES		YES
NO		NO
NO		NO
NO		NO
NO		NO
NO		YES
NO		NO
YES		YES
NO		YES
NO		NO
YES	TEMOZOLOMIDE	YES
NO		NO
NO		NO
NO		NO
YES	TEMOZOLOMIDE	YES
NO		NO
NO		NO
NO		NO
%)		
NO		NO
YES	TEMOZOLOMIDE	YES
NO		NO
NO		NO
NO		NO
NO		NO
NO		NO
NO		NO
NO		NO
YES		YES
NO		NO
NO		NO
NO		YES
NO		NO
NO		YES
NO		YES
NO		NO
YES	TEMOZOLOMIDE	YES
NO		YES

YES						YES
NO						NO
NO						NO
YES			FOTEMUSTINE			YES
YES			TEMOZOLOMIDE			YES
YES			TEMOZOLOMIDE			YES
YES			TEMOZOLOMIDE			YES
YES			TEMOZOLOMIDE			YES
YES			FOTEMUSTINE			YES
YES			FOTEMUSTINE			YES
YES			OTHER			YES
NO						NO
YES						YES
YES			TEMOZOLOMIDE			YES
YES			TEMOZOLOMIDE			YES
YES	YES		TEMOZOLOMIDE	FOTEMUSTINE		YES
YES			TEMOZOLOMIDE			YES
NO						YES
YES			TEMOZOLOMIDE			YES
YES			TEMOZOLOMIDE			YES
YES			TEMOZOLOMIDE			YES
YES			TEMOZOLOMIDE			YES
YES			TEMOZOLOMIDE			YES
YES			TEMOZOLOMIDE			YES
YES			TEMOZOLOMIDE			YES
YES			TEMOZOLOMIDE			YES
YES			TEMOZOLOMIDE			YES
YES	YES	YES	GLIADEL	TEMOZOLOMIDE	GLIADEL	YES
YES	YES	YES	TEMOZOLOMIDE	GLIADEL	FOTEMUSTINE	YES
YES			TEMOZOLOMIDE			YES
YES			TEMOZOLOMIDE			YES
YES	YES		TEMOZOLOMIDE	PVC		YES
YES			TEMOZOLOMIDE			YES
YES			TEMOZOLOMIDE			YES
YES			TEMOZOLOMIDE			YES
YES			TEMOZOLOMIDE			YES
YES			TEMOZOLOMIDE			YES
%)						NO
YES			TEMOZOLOMIDE			YES
YES			TEMOZOLOMIDE			YES
YES			TEMOZOLOMIDE			YES
YES			TEMOZOLOMIDE			YES

YES		TEMOZOLOMIDE		YES
YES		TEMOZOLOMIDE		YES
YES		TEMOZOLOMIDE		YES
YES		TEMOZOLOMIDE		YES
YES		TEMOZOLOMIDE		YES
NO				NO
YES		TEMOZOLOMIDE		YES
YES		TEMOZOLOMIDE		YES
YES		TEMOZOLOMIDE		YES
NO				YES
YES		TEMOZOLOMIDE		YES
NO				YES
YES		TEMOZOLOMIDE		YES
YES		TEMOZOLOMIDE		YES
YES	YES	TEMOZOLOMIDE	PVC	YES
YES		TEMOZOLOMIDE		YES
NO				NO
YES		TEMOZOLOMIDE		YES
NO				YES
NO				YES
YES	YES	TEMOZOLOMIDE	OTHER	YES
YES	YES	TEMOZOLOMIDE		YES
YES		TEMOZOLOMIDE		YES
YES	YES	TEMOZOLOMIDE	GLIADEL	YES
YES		TEMOZOLOMIDE		YES
YES	YES	TEMOZOLOMIDE	PVC	YES
NO				YES
YES		TEMOZOLOMIDE		YES
NO				YES
YES		TEMOZOLOMIDE		YES
YES		TEMOZOLOMIDE		YES
YES		TEMOZOLOMIDE		YES
YES		TEMOZOLOMIDE		YES
YES		TEMOZOLOMIDE		YES
NO				YES
YES		TEMOZOLOMIDE		YES
YES		TEMOZOLOMIDE		YES
NO				NO
YES		TEMOZOLOMIDE		YES
YES		TEMOZOLOMIDE		YES
YES		TEMOZOLOMIDE		YES
YES		TEMOZOLOMIDE		YES

YES			TEMOZOLOMIDE			YES
YES			TEMOZOLOMIDE			YES
YES			TEMOZOLOMIDE			YES
NO						YES
NO						NO
YES			TEMOZOLOMIDE			YES
NO						YES
YES			TEMOZOLOMIDE			YES
YES			TEMOZOLOMIDE			YES
YES			TEMOZOLOMIDE			YES
YES			TEMOZOLOMIDE			YES
YES			TEMOZOLOMIDE			YES
YES			TEMOZOLOMIDE			YES
YES			TEMOZOLOMIDE			YES
YES			TEMOZOLOMIDE			YES
YES			TEMOZOLOMIDE			YES
YES			TEMOZOLOMIDE			YES
YES	YES		TEMOZOLOMIDE	FOTEMUSTINE		YES
YES			TEMOZOLOMIDE			YES
NO						NO
YES			TEMOZOLOMIDE			YES
YES			TEMOZOLOMIDE			YES
YES			TEMOZOLOMIDE			YES
YES			TEMOZOLOMIDE			YES
YES	YES	YES	PVC	TEMOZOLOMIDE	GLIADEL	YES
NO						NO
YES	YES		PVC	TEMOZOLOMIDE		YES
YES			TEMOZOLOMIDE			YES
YES			TEMOZOLOMIDE			YES
YES	YES		PVC	TEMOZOLOMIDE		YES
YES	YES		PVC	TEMOZOLOMIDE		YES
NO						NO
YES	YES		TEMOZOLOMIDE	PVC		YES
YES	YES		TEMOZOLOMIDE	BEVACIZUMAB		YES
NO						NO
YES	YES	YES	TEMOZOLOMIDE	FOTEMUSTINE	BEVACIZUMAB	YES
YES	YES	YES	TEMOZOLOMIDE	FOTEMUSTINE	BEVACIZUMAB	YES
YES			TEMOZOLOMIDE			YES
YES			TEMOZOLOMIDE			YES
YES	YES	YES	TEMOZOLOMIDE	GLIADEL	FOTEMUSTINE	YES
YES			OTHER			YES

YES	YES		GLIADEL	TEMOZOLOMIDE		YES
YES			TEMOZOLOMIDE			YES
YES	YES	YES	FOTEMUSTINE	BEVACIZUMAB	OTHER	YES
YES	YES	YES	TEMOZOLOMIDE	GLIADEL	FOTEMUSTINE	YES
YES	YES		TEMOZOLOMIDE	GLIADEL		YES
YES			TEMOZOLOMIDE			YES
YES	YES		TEMOZOLOMIDE	OTHER		YES
YES			TEMOZOLOMIDE			YES
YES	YES	YES	GLIADEL	TEMOZOLOMIDE	FOTEMUSTINE	YES
YES	YES	YES	TEMOZOLOMIDE	GLIADEL	FOTEMUSTINE	YES
NO						NO
YES	YES	YES	GLIADEL	TEMOZOLOMIDE	FOTEMUSTINE	YES
YES	YES	YES	TEMOZOLOMIDE	OTHER	BEVACIZUMAB	YES
YES			TEMOZOLOMIDE			YES
YES			OTHER			YES
YES	YES	YES	GLIADEL	TEMOZOLOMIDE	FOTEMUSTINE	YES
YES	YES		TEMOZOLOMIDE	FOTEMUSTINE		YES
YES	YES	YES	TEMOZOLOMIDE	BEVACIZUMAB	OTHER	YES
YES	YES		FOTEMUSTINE	TEMOZOLOMIDE		YES
NO						NO
YES	YES		TEMOZOLOMIDE	OTHER		YES
NO						NO
YES			TEMOZOLOMIDE			YES
YES			TEMOZOLOMIDE			YES
YES	YES		TEMOZOLOMIDE	FOTEMUSTINE		YES
YES			TEMOZOLOMIDE			YES
YES	YES	YES	TEMOZOLOMIDE	FOTEMUSTINE	BEVACIZUMAB	YES
YES	YES	YES	TEMOZOLOMIDE	BEVACIZUMAB	FOTEMUSTINE	YES
YES			TEMOZOLOMIDE			YES
YES	YES		TEMOZOLOMIDE	BEVACIZUMAB		YES
NO						NO
YES			TEMOZOLOMIDE			YES
YES			OTHER			YES
NO						NO
NO						YES
NO						NO
YES			TEMOZOLOMIDE			YES
NO						NO
%)						
NO						NO
YES			TEMOZOLOMIDE			YES
NO						NO

YES			PVC			
YES			PVC			
NO						NO
YES			OTHER			YES
NO						YES
YES			TEMOZOLOMIDE			YES
YES			PVC			YES
YES			OTHER			YES
YES			OTHER			YES
YES	YES	YES	FOTEMUSTINE	PVC	TEMOZOLOMIDE	NO
YES						YES
YES			PVC			YES
YES			TEMOZOLOMIDE			NO
YES			TEMOZOLOMIDE			YES
NO						YES
YES	YES		TEMOZOLOMIDE	PVC		YES
YES			TEMOZOLOMIDE			
YES	YES		TEMOZOLOMIDE	FOTEMUSTINE		YES
YES			TEMOZOLOMIDE			YES
NO						NO
NO						NO
NO						NO
NO						NO
NO						NO
YES	YES		TEMOZOLOMIDE	PVC		YES
NO						YES
%)						YES
NO						NO
%)						
NO						NO
						NO
NO						NO
YES			TEMOZOLOMIDE			YES
YES			TEMOZOLOMIDE			YES
YES			TEMOZOLOMIDE			NO
NO						NO
YES			OTHER			YES

YES	YES	TEMOZOLOMIDE	FOTEMUSTINE	NO
YES	YES	TEMOZOLOMIDE	FOTEMUSTINE	YES
YES		TEMOZOLOMIDE		YES
YES		TEMOZOLOMIDE		YES
YES		OTHER		YES
NO				NO
YES	YES	TEMOZOLOMIDE	BEVACIZUMAB	YES
NO				NO
YES		TEMOZOLOMIDE		YES
%)				
YES		TEMOZOLOMIDE		YES
NO				YES
YES		TEMOZOLOMIDE		NO
YES		TEMOZOLOMIDE		YES
YES	YES	TEMOZOLOMIDE	FOTEMUSTINE	YES
YES		TEMOZOLOMIDE		YES
YES		TEMOZOLOMIDE		YES
NO				YES
NO				YES
YES		TEMOZOLOMIDE		YES
NO				NO
NO				NO
YES	YES	TEMOZOLOMIDE	OTHER	YES
YES		TEMOZOLOMIDE		YES
YES		TEMOZOLOMIDE		NO
%)				
%)				
YES		FOTEMUSTINE		YES
YES		TEMOZOLOMIDE		NO
YES		TEMOZOLOMIDE		YES
YES		TEMOZOLOMIDE		YES
NO				NO
YES	YES	TEMOZOLOMIDE	BEVACIZUMAB	YES
YES		TEMOZOLOMIDE		YES
YES		TEMOZOLOMIDE		YES
NO				NO
YES	YES	TEMOZOLOMIDE	FOTEMUSTINE	YES
YES	YES	TEMOZOLOMIDE	OTHER	YES
YES		TEMOZOLOMIDE		YES
YES		TEMOZOLOMIDE		YES

NO				NO
YES	YES	TEMOZOLOMIDE	FOTEMUSTINE	YES
NO				NO
NO				NO
NO				NO
YES		FOTEMUSTINE		YES
YES		TEMOZOLOMIDE		YES
NO				NO
%)				
NO				NO
YES		TEMOZOLOMIDE		NO
YES	YES	TEMOZOLOMIDE	GLIADEL	YES
%)				
YES		TEMOZOLOMIDE		YES
YES		TEMOZOLOMIDE		NO
NO				NO
YES		TEMOZOLOMIDE		YES
%)				
YES		TEMOZOLOMIDE		YES
YES		TEMOZOLOMIDE		YES
YES		TEMOZOLOMIDE		YES
YES		TEMOZOLOMIDE		YES
YES		TEMOZOLOMIDE		YES
YES		TEMOZOLOMIDE		YES
%)				
YES		TEMOZOLOMIDE		YES
YES		TEMOZOLOMIDE		YES
%)				
YES		OTHER		YES
YES		TEMOZOLOMIDE		YES
YES		TEMOZOLOMIDE		YES
YES		TEMOZOLOMIDE		YES
YES		TEMOZOLOMIDE		YES
YES	YES	TEMOZOLOMIDE	GLIADEL	YES
YES		GLIADEL		
YES		TEMOZOLOMIDE		YES
YES		TEMOZOLOMIDE		YES
YES		TEMOZOLOMIDE		YES
%)				
YES		TEMOZOLOMIDE		YES

%)						YES
YES			TEMOZOLOMIDE			YES
YES	YES		TEMOZOLOMIDE	FOTEMUSTINE		YES
NO						YES
%)						
YES	YES	YES	TEMOZOLOMIDE	FOTEMUSTINE	BEVACIZUMAB	YES
%)						
YES	YES		GLIADEL	TEMOZOLOMIDE		YES
YES			TEMOZOLOMIDE			YES
NO						NO
YES			TEMOZOLOMIDE			YES
YES			TEMOZOLOMIDE			YES
%)						
YES			TEMOZOLOMIDE			YES
YES	YES		TEMOZOLOMIDE	BEVACIZUMAB		YES
NO						YES
NO						YES
YES	YES		TEMOZOLOMIDE	GLIADEL		YES
YES			TEMOZOLOMIDE			YES
NO						YES
YES			OTHER			YES
YES	YES		TEMOZOLOMIDE	BEVACIZUMAB		YES
YES			TEMOZOLOMIDE			YES
YES			TEMOZOLOMIDE			YES
%)						
YES	YES	YES	TEMOZOLOMIDE	OTHER	BEVACIZUMAB	YES
YES	YES	YES	TEMOZOLOMIDE	FOTEMUSTINE	BEVACIZUMAB	YES
YES			TEMOZOLOMIDE			YES
NO						YES
YES	YES		TEMOZOLOMIDE	PVC		YES
NO						YES
%)						
YES			TEMOZOLOMIDE			NO
YES			TEMOZOLOMIDE			YES
YES	YES		TEMOZOLOMIDE	FOTEMUSTINE		YES
%)						
%)						
NO						NO
YES			TEMOZOLOMIDE			YES
NO						NO
YES			TEMOZOLOMIDE			YES

YES	TEMOZOLOMIDE	YES
NO		YES
YES	TEMOZOLOMIDE	YES
YES	TEMOZOLOMIDE	YES
NO		YES
NO		NO
NO		NO
NO		YES
YES	TEMOZOLOMIDE	YES
YES	TEMOZOLOMIDE	YES
YES	TEMOZOLOMIDE	YES
YES	TEMOZOLOMIDE	YES
YES	TEMOZOLOMIDE	YES

Radiotherapy1_type	STATUS	CRISIS	CRISIS INTERVENTION	CRISIS AT LAST CONTROL	MODIFY _AED	WHY	Antiepileptic1	Antiepileptic2	Antiepileptic3	months_fup
WHOLE BRAIN RT		PC	0.00	NO	NO		OXC			13.00
WHOLE BRAIN RT		PS+PC	before	YES	YES	SIDE EFFECTS+UNCONTROLLEI	PHT	OXC	LEV	13.00
WHOLE BRAIN RT		PC+SGTC	after	YES	NO		LEV			14.00
IMRT		PC	0.00	NO	YES	SIDE EFFECTS	CBZ	LEV		14.00
WHOLE BRAIN RT		PC	before	YES	YES	UNCONTROLLED SEIZURE	OXC	LEV		15.00
WHOLE BRAIN RT		PS	0.00	NO	NO		LEV			18.00
WHOLE BRAIN RT		GTC	0.00	NO	YES		LEV			19.00
WHOLE BRAIN RT		PC	after	NO	NO		OXC			19.00
WHOLE BRAIN RT		PS	0.00	NO	YES	SIDE EFFECTS	PG	TPM		21.00
WHOLE BRAIN RT		PC	0.00	NO	NO		LEV			24.00
		PS	before	NO	NO		LEV			26.00
WHOLE BRAIN RT		PC	0.00	YES	NO	UNCONTROLLED SEIZURE	OXC	LEV		26.00
		PC	after	YES	NO		LEV			27.00
		PC+SGTC	before	NO	YES	SIDE EFFECTS	OXC	LEV		28.00
		PC+SGTC	after	NO	NO		LEV			29.00
		PC+SGTC	before	NO	NO		OXC			29.00
		PC+SGTC	before	NO	YES	SIDE EFFECTS	PHT	LEV		29.00
		PS+SGTC	after	NO	NO		LEV			30.00
WHOLE BRAIN RT		PC+SGTC	before	NO	YES	UNCONTROLLED SEIZURE	OXC	LEV		30.00
STEREOTASSICA		PS	after	NO	YES	UNCONTROLLED SEIZURE	PG	LEV		30.00
		PS	before	NO	YES	SIDE EFFECTS	CBZ	OXC		30.00
		PC	before	NO	NO		OXC			31.00
		PC	before	NO	YES	UNCONTROLLED SEIZURE	CBZ	LEV		31.00
		PC+SGTC	before	NO	NO		LEV			32.00
		PS+SGTC	before	NO	NO		OXC			32.00
STEREOTASSICA		PC+SGTC	before	NO	NO		CBZ			33.00
		PC	before	NO	NO		LEV			33.00
		PC+SGTC	before	NO	NO		OXC			33.00
		PC	before	YES	YES	UNCONTROLLED SEIZURE	PHT	LEV	OXC	33.00
		PC	before	NO	NO		LEV			34.00
WHOLE BRAIN RT		PC+SGTC	before	NO	YES	SIDE EFFECTS+UNCONTROLLEI	CBZ	OXC	LEV	35.00
		PC	before	NO	NO		OXC			36.00
		PC	after	NO	NO		OXC			36.00
		PC+SGTC	before	NO	NO		OXC			37.00
		PC	before	NO	YES	SIDE EFFECTS	PHT	OXC		37.00
		PC	after	NO	NO		CBZ			38.00
		PS+SGTC	before	NO	NO		PG			40.00
		PC	before	NO	YES	SIDE EFFECTS	PG	CBZ		41.00
WHOLE BRAIN RT		PC+SGTC	after	YES	YES	UNCONTROLLED SEIZURE	PG	OXC	LEV	45.00

WHOLE BRAIN RT		PC	before		YES	YES	UNCONTROLLED SEIZURE	VPA	OXC	LEV	50.00
WHOLE BRAIN RT		PC	before		NO	YES	UNCONTROLLED SEIZURE	PG	TPM	OXC	50.00
		PS+PC	before					CBZ			
		PS	after		YES	YES	UNCONTROLLED SEIZURE	OTHER	LEV	LEV+CBZ	15.00
		PS	before								
		PS	before					OXC			
		PC	before					PG			48.00
			before		NO			OXC			96.00
		PS+SGTC	before								
		PS+SGTC	before					CBZ			
IMRT		PS	before								
			before		NO	YES	SCARCE COMPLIANCE	CBZ	TPM	LEV	120.00
CONFORMATION AL RT		PC	before		YES	YES	UNCONTROLLED SEIZURE	LEV	LEV+OTHER		24.00
		PC	before		YES	YES	UNCONTROLLED SEIZURE	PG	VPA	LEV+VPA	48.00
CONFORMATION AL RT		PC+SGTC	before		NO	NO		VPA			72.00
		PC	before		NO	YES	SIDE EFFECTS	VPA	LEV		24.00
		PC	before		YES	YES	SIDE EFFECTS	VPA	LEV		29.00
		PS+SGTC	before		YES	YES	SIDE EFFECTS	VPA	LEV		24.00
WHOLE BRAIN RT		PC	before		YES	YES	UNCONTROLLED SEIZURE	VPA	LEV+VPA		33.00
		PC+SGTC	before		NO	NO		VPA			24.00
CONFORMATION AL RT	NOT CONVULSI	PS	before		NO	YES	SE+OTHER	PHT	TPM		228.00
	NOT CONVULSI	PS	before		NO	YES	OTHER	PG	LEV+PGB		171.00
CONFORMATION AL RT	NOT CONVULSI	PS	before		NO	NO		OXC			101.00
CONFORMATION AL RT	NOT CONVULSI	PS	before		NO	YES		VPA	TPM		48.00
CONFORMATION AL RT	NOT CONVULSI	PS	before		NO	YES	OTHER	LEV+OXC	LEV+TPM		27.00
CONFORMATION AL RT	NOT CONVULSI	PS	before		NO	NO		LEV			27.00
CONFORMATION AL RT	NOT CONVULSI	PS	before		NO	NO		LEV			25.00
WHOLE BRAIN RT		PS		0.00	NO			LEV			16.00
CONFORMATION AL RT	NOT CONVULSI	PS	before		YES	YES	UNCONTROLLED SEIZURE	LEV	LEV+TPM		12.00
CONFORMATION AL RT	CONVULSIVE	PC+SGTC		0.00	NO	NO		LEV			18.00
	CONVULSIVE	PC+SGTC		0.00	NO	YES	UNCONTROLLED SEIZURE	OXC			18.00
CONFORMATION AL RT	NOT CONVULSI	PS	before		NO	YES	OTHER	PG	LEV		101.00
CONFORMATION AL RT	NOT CONVULSI	PS	after		NO	YES	UNCONTROLLED SEIZURE	LEV	OXC	ZNS	7.00
		PS		0.00	NO			LEV			2.00
	NOT CONVULSI	PS		0.00	NO			LEV			2.00
	NOT CONVULSI	PS		0.00	NO			LEV			1.00
CONFORMATION AL RT				0.00	NO	YES	SIDE EFFECTS	PHT	OXC		79.00
WHOLE BRAIN RT				0.00	NO						27.00
CONFORMATION AL RT				0.00	NO	YES	SIDE EFFECTS	CBZ	OXC		57.00
				0.00	NO						28.00
				0.00	NO						24.00
WHOLE BRAIN RT	NOT CONVULSIVE			0.00	NO	NO		LEV			14.00

WHOLE BRAIN RT				0.00	NO						11.00
STEREOTASSICA				0.00	NO						41.00
CONFORMATION AL RT	NOT CONVULSI	PS		0.00	NO	NO		LEV			19.00
				0.00	NO	NO		LEV			12.00
CONFORMATION AL RT	NOT CONVULSIVE			0.00	NO	NO		LEV			11.00
CONFORMATION AL RT				0.00	NO			LEV			10.00
IMRT				0.00	NO	YES	SIDE EFFECTS	LEV	PHT		9.00
WHOLE BRAIN RT	NOT CONVULSI	PS		0.00	NO			VPA			1.00
				0.00	NO						7.00
CONFORMATION AL RT	NOT CONVULSI	PS	before		NO	YES	OTHER	PG	LEV	LCM+LEV	36.00
CONFORMATION AL RT				0.00	NO	NO		LEV			5.00
CONFORMATION AL RT				0.00	NO			LEV			228.00
	NOT CONVULSI	PS	before		YES			VPA			2.00
CONFORMATION AL RT				0.00		NO		LEV			
	NOT CONVULSIVE			0.00	NO			CBZ			5.00
		GTC		0.00	NO	NO		LEV			12.00
		PC	before		NO						12.00
		PC+SGTC	before		YES	YES		OXC			12.00
		PC		0.00	NO	YES	UNCONTROLLED SEIZURE	PG	OXC		3.00
RADIOSURGERY		PS	before		YES	NO		CBZ			0.00
		PC		0.00	YES	NO		CBZ			14.00
		PC		0.00	YES	YES	SIDE EFFECTS+UNCONTROLLEI	PG	CBZ	TGB	88.00
		PC+SGTC	before		NO	NO		OXC			12.00
		PS	before		YES	YES	UNCONTROLLED SEIZURE	PHT	LEV	CBZ	8.00
IMRT		GTC	before		NO			LEV			7.00
IMRT		PC+SGTC	before		NO			LEV			72.00
WHOLE BRAIN RT		PS	before		YES	YES	UNCONTROLLED SEIZURE	PHT	CBZ	ZNS	14.00
		PC	before		YES	YES	UNCONTROLLED SEIZURE	CBZ	VPA	LEV	30.00
WHOLE BRAIN RT		PS+SGTC	before		YES	NO		LEV	CBZ		6.00
		PS	before		YES	YES	SIDE EFFECTS	CBZ	OXC	LCM	6.00
		PS	before		YES	NO		PG			6.00
		PS	before		YES	NO		LEV			6.00
WHOLE BRAIN RT		PS+SGTC	after		YES	NO		LEV			6.00
		PS+SGTC	before		NO	YES	UNCONTROLLED SEIZURE	TPM	CBZ		6.00
		PC+SGTC	after		NO	YES	UNCONTROLLED SEIZURE	PG	CBZ	VPA	6.00
		PS+SGTC		0.00	NO	NO		CBZ	LEV		6.00
		PS	before		YES	NO		PG	LCM		6.00
		PS+SGTC	before		NO	YES	SIDE EFFECTS	PG	CBZ	LEV	6.00
		PS+SGTC	before		NO	YES	UNCONTROLLED SEIZURE	CBZ	LEV	TPM	6.00
		PS	before		NO	YES	SIDE EFFECTS	OXC	LEV		4.00
CONFORMATION AL RT	CONVULSIVE		before		YES	YES	UNCONTROLLED SEIZURE	PG	CBZ	LEV	2.00
CONFORMATION AL RT		PS+SGTC	after		YES	YES	SIDE EFFECTS+UNCONTROLLEI	PG	LEV	VPA	8.00

RADIOSURGERY		PS+SGTC		0.00	NO	YES	SIDE EFFECTS	PG	LEV		4.00
STEREOTASSICA		PS	before		NO	YES	SIDE EFFECTS	PG	OXC		12.00
CONFORMATION AL RT		PC+SGTC	after		NO	NO		OXC			5.00
		PC+SGTC	before		NO	YES	SCARCE COMPLIANCE	LEV	TPM		30.00
CONFORMATION AL RT		PS	after		NO	NO		OXC			3.00
		PS+SGTC	after		NO	NO		LEV			29.00
CONFORMATION AL RT		PS+PC	before		NO	YES	UNCONTROLLED SEIZURE	PG	VPA		1.00
		GTC	after		NO	NO		LEV			1.00
CONFORMATION AL RT		PS	before		NO	YES	SIDE EFFECTS	OXC	VPA		13.00
RADIOSURGERY		PS	after		YES	YES	SIDE EFFECTS	PG			11.00
CONFORMATION AL RT		PS	after		YES	NO		OTHER			2.00
IMRT		PS+PC	before		YES	YES	SIDE EFFECTS	OXC	VPA		13.00
CONFORMATION AL RT		PS	before		YES	NO		OXC			6.00
		PC+SGTC	after		NO	NO		OXC			11.00
CONFORMATION AL RT		PS	after		YES	YES	UNCONTROLLED SEIZURE	OXC	LEV		9.00
CONFORMATION AL RT		PC	before		NO	YES	SIDE EFFECTS	PG	ZNS		5.00
		PS	after		YES	YES	SCARCE COMPLIANCE	CBZ	PG	LEV	32.00
		PS	before		YES	YES	SCARCE COMPLIANCE	OXC	LEV		18.00
CONFORMATION AL RT		PS	after		YES	YES	UNCONTROLLED SEIZURE	OXC	LEV		11.00
CONFORMATION AL RT		PS	after		NO	NO		OXC			9.00
	NOT CONVULSI	PS	before		YES	YES	SIDE EFFECTS+UNCONTROLLEI	OXC	LEV	ZNS	31.00
CONFORMATION AL RT		PC	before		YES	YES	UNCONTROLLED SEIZURE	OXC	LEV		13.00
		PS	after		NO	NO		OXC			5.00
CONFORMATION AL RT		PS+SGTC	before		NO	NO		VPA			7.00
	NOT CONVULSI	PS+SGTC	before		NO	YES	UNCONTROLLED SEIZURE	PG	OXC		34.00
WHOLE BRAIN RT		PS+SGTC		0.00	NO	NO		OXC			17.00
IMRT		PS+PC	after		YES	YES	UNCONTROLLED SEIZURE	LEV	OXC		14.00
CONFORMATION AL RT		GTC	after		NO	NO		LEV			19.00
CONFORMATION AL RT		PC+SGTC	before		NO	YES	SIDE EFFECTS	LEV	OXC		4.00
CONFORMATION AL RT		PS+PC	after		YES	YES	UNCONTROLLED SEIZURE	PG	OXC	VPA	18.00
CONFORMATION AL RT		PS+PC	after		YES	NO		LEV			3.00
CONFORMATION AL RT		GTC	after		NO	YES	SIDE EFFECTS	PG	LEV		11.00
CONFORMATION AL RT		PS+SGTC	before		NO	YES	SIDE EFFECTS	CBZ	LEV		47.00
IMRT	CONVULSIVE	PS	before		NO	YES	UNCONTROLLED SEIZURE	PG	LEV	VPA	10.00
WHOLE BRAIN RT		PS		0.00	YES	NO		LEV			1.00
CONFORMATION AL RT		PS	before		YES	YES	UNCONTROLLED SEIZURE	OXC	LEV	VPA	15.00
CONFORMATION AL RT		PS+PC	after		NO	YES	UNCONTROLLED SEIZURE	PG	LEV		39.00
IMRT	NOT CONVULSI	PS+SGTC		0.00	NO	YES	SIDE EFFECTS+UNCONTROLLEI	OXC	CBZ	VPA	5.00
CONFORMATION AL RT		PS	before		YES	YES	SIDE EFFECTS+UNCONTROLLEI	PG	TPM	LEV	16.00
CONFORMATION AL RT		PS	before		YES	YES	SCARCE COMPLIANCE	LEV	CBZ	LCM	11.00
		PC	before		NO	YES	SCARCE COMPLIANCE	CBZ	OXC	LEV	3.00
CONFORMATION AL RT		PS	before		NO	NO		CBZ			1.00

CONFORMATION AL RT		PS+SGTC		0.00	NO	YES	SIDE EFFECTS	CBZ	LEV		12.00
CONFORMATION AL RT		PS+SGTC	before		YES	YES	UNCONTROLLED SEIZURE	OXC	LEV		14.00
CONFORMATION AL RT	NOT CONVULSI	PC+SGTC	before		YES	NO		OXC			8.00
WHOLE BRAIN RT		PS+SGTC	after		NO	YES	SCARCE COMPLIANCE	PG	OXC		11.00
CONFORMATION AL RT		PS	after		NO	NO		LEV			5.00
		PS	after		YES	YES	SIDE EFFECTS+UNCONTROLLEI	OXC	LEV	OTHER	22.00
RADIOSURGERY		PS	before		YES	YES	UNCONTROLLED SEIZURE	TPM	LEV		28.00
IMRT	NOT CONVULSI	PC+SGTC	after		YES	YES	SIDE EFFECTS+UNCONTROLLEI	PG	OXC	CBZ	17.00
		PS+SGTC	after		NO	YES	SIDE EFFECTS	PG	LEV		32.00
CONFORMATION AL RT		PS	before		NO	NO		LEV			7.00
CONFORMATION AL RT		PC+SGTC	after		NO	YES	SIDE EFFECTS	OXC	LEV	VPA	36.00
		PC		0.00	NO	NO		LEV			5.00
	CONVULSIVE	PC+SGTC	before		YES	YES	UNCONTROLLED SEIZURE	PG	PHT		48.00
WHOLE BRAIN RT		PS	before			NO		LEV			1.00
WHOLE BRAIN RT		PS	before		NO	NO		PHT			4.00
WHOLE BRAIN RT		PC		0.00	YES	YES	UNCONTROLLED SEIZURE	PG	LEV		1.00
		PC	before			YES	UNCONTROLLED SEIZURE	PG	PHT	LEV	2.00
WHOLE BRAIN RT		PS+PC	before		NO	YES	UNCONTROLLED SEIZURE	LEV	TPM		10.00
WHOLE BRAIN RT		PS		0.00		NO		TPM			1.00
WHOLE BRAIN RT		PC	after		YES	YES	UNCONTROLLED SEIZURE	PHT	PG		1.00
		PC	before					LEV			1.00
		PS+PC		0.00	NO	NO		CBZ			5.00
WHOLE BRAIN RT		PS	before			YES	UNCONTROLLED SEIZURE	TPM	CBZ		18.00
		PC	before		YES	YES	UNCONTROLLED SEIZURE	VPA	LEV	CBZ	40.00
		PC+SGTC	before		NO	YES	UNCONTROLLED SEIZURE	PG	LEV	CBZ	14.00
		PS+SGTC	before		YES	YES	SIDE EFFECTS	OXC	PG		23.00
	CONVULSIVE	PS	before		YES	NO		PHT	OTHER		4.00
IMRT		GTC	before+after		YES	NO		LEV			0.00
IMRT		PC	before		NO	NO		VPA			41.00
		GTC	before		NO	YES	UNCONTROLLED SEIZURE	OTHER	CBZ		34.00
		PC	before		NO	NO		CBZ			34.00
		PC	before		NO	NO		OTHER			43.00
		PS+SGTC	before+after		YES	YES	UNCONTROLLED SEIZURE	OTHER	VPA		29.00
		PC	before		NO	NO		VPA			43.00
IMRT		PS+SGTC	after		NO	YES	SIDE EFFECTS+UNCONTROLLEI	PG	OXC	LEV	42.00
		PC+SGTC	after		YES	YES	UNCONTROLLED SEIZURE	LEV	LCM		12.00
		GTC	after		NO			LEV			30.00
		PS	after		NO	YES	UNCONTROLLED SEIZURE	OXC	LEV	TPM	100.00
		PC	before		YES	YES	SIDE EFFECTS	PG	LEV		24.00
		PS	before		NO	YES	SIDE EFFECTS+UNCONTROLLEI	PG	LEV		43.00
IMRT		GTC	before		NO	YES	UNCONTROLLED SEIZURE	PG	OXC	VPA	72.00
		PC+SGTC	before		NO	YES	UNCONTROLLED SEIZURE	PG	LEV	LTG	55.00

	PS+SGTC	before	YES	YES	UNCONTROLLED SEIZURE	VPA	LEV	PGB	48.00
IMRT	PC+SGTC	after	YES	YES	UNCONTROLLED SEIZURE	CBZ	LEV		24.00
WHOLE BRAIN RT	PS	before	NO	NO		LEV	ZNS		14.00
IMRT	GTC	before	YES	YES	UNCONTROLLED SEIZURE	CBZ	LEV	LCM	61.00
	PS	before	YES	YES	UNCONTROLLED SEIZURE	LEV	VPA	PG	132.00
	GTC	before	NO	YES	UNCONTROLLED SEIZURE	LEV	TPM	PGB	45.00
	PC+SGTC	before	NO	YES	UNCONTROLLED SEIZURE	LEV	LEV		93.00
	PC+SGTC	before	NO	NO		PG			88.00
STEREOTASSICA	GTC	before	NO	NO		PG			14.00
	PS	before	NO	NO		TPM			84.00
WHOLE BRAIN RT	GTC	before	NO	YES	UNCONTROLLED SEIZURE	OXC	LTG	PG	63.00
	GTC	after	NO			PG			14.00
WHOLE BRAIN RT	PC+SGTC	before	NO	YES	SCARCE COMPLIANCE	PG	OXC		27.00
WHOLE BRAIN RT	GTC	before	NO	YES	UNCONTROLLED SEIZURE	OXC			12.00
	PS	before	NO	YES	SCARCE COMPLIANCE	PHT	LTG		52.00
IMRT	PS+SGTC	before	NO	YES	SCARCE COMPLIANCE	PG	OXC		63.00
IMRT	PC+SGTC	before	NO			LEV			36.00
WHOLE BRAIN RT	PS	after	NO			LEV			29.00
	GTC	before	NO	YES	UNCONTROLLED SEIZURE	CBZ	TPM		70.00
	PS	after	NO	NO		LEV			16.00
	PS	before	NO	YES	UNCONTROLLED SEIZURE	PG	LEV		16.00
	PS	before	NO	NO		LEV			29.00
	PS	before	NO	YES	UNCONTROLLED SEIZURE	LEV	PG		14.00
IMRT	PC+SGTC	before	YES	YES	UNCONTROLLED SEIZURE	CBZ	LCM		108.00
IMRT	PS+SGTC	before	NO	YES	SCARCE COMPLIANCE	PG	VPA		117.00
	PC+SGTC	before	NO	YES	UNCONTROLLED SEIZURE	OXC	CBZ	TPM	84.00
IMRT	PC+SGTC	before	NO	YES	SCARCE COMPLIANCE	CBZ			72.00
	PS	before	NO			TPM			83.00
	PC	before	NO	YES	UNCONTROLLED SEIZURE	CBZ	ZNS		96.00
	PC	after	YES	YES	UNCONTROLLED SEIZURE	CBZ	LEV		144.00
	PS+SGTC	before	YES	YES	UNCONTROLLED SEIZURE	LEV	OXC		15.00
	PC+SGTC	after	NO	NO		LEV			12.00
IMRT	PC	before	YES	NO		LEV			12.00
	PC	after	NO	YES	SCARCE COMPLIANCE	LEV	OXC		12.00
WHOLE BRAIN RT	GTC	before	NO	NO		LEV			108.00
	GTC	before	YES	YES	UNCONTROLLED SEIZURE	CBZ	OXC	LEV	12.00
	PC	before	NO	YES	UNCONTROLLED SEIZURE	LEV	LTG	TPM	12.00
	PC	before	YES	YES	UNCONTROLLED SEIZURE	PG	LEV	CBZ	17.00
	GTC	after	NO	NO		PG			192.00
	PS	after	YES	YES	UNCONTROLLED SEIZURE	PG	OXC	LCM	16.00
	PC+SGTC	before	NO	YES	SIDE EFFECTS+UNCONTROLLEI	OXC	PG	PHT	27.00
	GTC	before	NO	YES	UNCONTROLLED SEIZURE	PHT	CBZ		33.00

	PS	before	NO	YES	SE+OTHER	LEV	CBZ	TPM	14.00
IMRT	PC	before	YES	YES	UNCONTROLLED SEIZURE	PG	LEV		24.00
WHOLE BRAIN RT	PC	before	YES			LEV			19.00
WHOLE BRAIN RT	PC+SGTC	after	NO	NO		OXC			5.00
WHOLE BRAIN RT	PC	after	NO	NO		PG			12.00
WHOLE BRAIN RT	PC+SGTC	before	NO	NO		TPM			132.00
	PS+SGTC	before	NO	YES	UNCONTROLLED SEIZURE	PG	LEV	PHT	19.00
	PS+SGTC	after	NO	YES	UNCONTROLLED SEIZURE	LEV	CBZ	TPM	36.00
STEREOTASSICA	PC+SGTC	before	NO	YES	UNCONTROLLED SEIZURE	LTG	LEV	LCM	34.00
IMRT	PS+SGTC	before	YES	YES	UNCONTROLLED SEIZURE	LEV	PGB	LCM	12.00
IMRT	PS	before	YES	YES	UNCONTROLLED SEIZURE	PG	LEV		12.00
	PC+SGTC	before	NO	NO		OXC			46.00
	PS	before	YES	YES	UNCONTROLLED SEIZURE	TPM	PGB		65.00
	GTC	before	YES			LEV			24.00
	GTC	before	NO	NO		LEV			24.00
WHOLE BRAIN RT	PC	after	YES	YES	UNCONTROLLED SEIZURE	LEV	LCM		11.00
	GTC	before	YES	YES	UNCONTROLLED SEIZURE	LEV	LCM		12.00
WHOLE BRAIN RT	PS	after	NO	NO		LEV			12.00
WHOLE BRAIN RT	PC+SGTC	before	YES	YES	UNCONTROLLED SEIZURE	LEV	PHT		36.00
	GTC	after	NO	YES	SCARCE COMPLIANCE	PG	TPM		6.00
WHOLE BRAIN RT	PS	before	NO	YES	UNCONTROLLED SEIZURE	OXC	LEV		12.00
STEREOTASSICA	PS+SGTC	before	YES	YES	SIDE EFFECTS+UNCONTROLLEI	PHT	LEV	PG	6.00
WHOLE BRAIN RT	PS	after	NO			PG	LEV		6.00
WHOLE BRAIN RT	PS	after	YES	YES	UNCONTROLLED SEIZURE	OXC	LEV		6.00
WHOLE BRAIN RT	PS	after	NO	NO		LEV			6.00
WHOLE BRAIN RT	PC	after	YES	YES	UNCONTROLLED SEIZURE	LEV	PGB		12.00
WHOLE BRAIN RT	PS	after	NO	NO		OXC			8.00
STEREOTASSICA	PC	after	NO			LEV			72.00
	PS	before	YES	YES	UNCONTROLLED SEIZURE	OXC	LCM		96.00
WHOLE BRAIN RT	PS	before	NO	YES	SIDE EFFECTS	LEV	OXC		24.00
	PS	before	NO	YES	SIDE EFFECTS	LEV	OXC		5.00
WHOLE BRAIN RT	PC+SGTC	after	YES	YES	SCARCE COMPLIANCE	PG	LEV		12.00
		after	NO			PG			6.00
RADIOSURGERY	PS+SGTC	after	NO			PG			10.00
WHOLE BRAIN RT	PC	after	NO	YES	UNCONTROLLED SEIZURE	LEV	TPM		4.00
		before	NO			CBZ			4.00
	PS+SGTC	after	NO			TPM			23.00
	PC+SGTC	after	NO			OXC			6.00
STEREOTASSICA	PS+SGTC	after	NO			PG			4.00
		after	NO			LEV			3.00
WHOLE BRAIN RT	PS+SGTC	after	NO			PG			20.00
	PS+SGTC	after	NO			OXC			6.00

	PS	after		NO			LEV			8.00
WHOLE BRAIN RT	PS+SGTC	after		NO			LEV			23.00
	GTC	before		NO	YES	SIDE EFFECTS	PG	OXC		
	PS+SGTC		0.00	YES	NO		OXC			12.00
	GTC	before		NO	YES	SIDE EFFECTS	PHT	PG	LEV	8.00
	PS		0.00	NO	NO		OXC			
	PS+SGTC		0.00	NO	NO		PG	LEV		
CONFORMATION AL RT	PC	before		NO	NO		OTHER			43.00
IMRT			0.00	NO						
WHOLE BRAIN RT	PC	after		NO	YES	SIDE EFFECTS	PGB	PG	LEV	6.00
	PC	before		NO			PG			26.00
WHOLE BRAIN RT	PC		0.00				LEV			10.00
	PC	before		NO	YES		PG	OXC		1.00
WHOLE BRAIN RT	PC	after		NO	YES	SIDE EFFECTS	PG	LEV		18.00
	PS	before		NO			PG			48.00
IMRT	PC		0.00	NO			LEV			36.00
IMRT	PC	before		NO	YES	SIDE EFFECTS	PG	PHT	LEV	2.00
	PS	before		NO	YES	SIDE EFFECTS	PG	CBZ	TPM	12.00
	PC	before		NO			CBZ			48.00
	PC	before		NO			LEV			36.00
	PC	after		NO			PG			60.00
WHOLE BRAIN RT			0.00	NO	YES	SIDE EFFECTS	PG	LEV		3.00
WHOLE BRAIN RT	PS	before		NO	YES	SIDE EFFECTS	CBZ	PG	PHT	45.00
WHOLE BRAIN RT	PC	before		NO	YES	SIDE EFFECTS	PG	LEV		48.00
IMRT	PC	before		NO	YES	SIDE EFFECTS	LEV	PGB		8.00
	PC	before		NO			LEV			41.00
WHOLE BRAIN RT	PC		0.00	NO	YES	SIDE EFFECTS	PHT	LEV		40.00
WHOLE BRAIN RT	PC	before		NO	NO		LEV	PG		
	PC	before		NO			PG			
IMRT	PC	before		NO			PG	LEV		1.00
	PC		0.00	NO	YES	SIDE EFFECTS	PG			10.00
			0.00	NO			CBZ	PG		
WHOLE BRAIN RT	PC	after		NO			LEV			12.00
	PC	before		YES			PG	OXC		31.00
WHOLE BRAIN RT	PC	before		NO	YES	SIDE EFFECTS	PG	LEV		36.00
IMRT	PC	after		NO			PG			15.00
	PC	before		NO			LEV			60.00
	PC	after		NO			LEV			60.00
WHOLE BRAIN RT	PC	after		NO			PG			6.00
CONFORMATION AL RT	PC	before		YES	YES	UNCONTROLLED SEIZURE	CBZ	PG		42.00
	PS+PC	before		NO	NO		OTHER			30.00
	PS+PC	after		NO	YES	UNCONTROLLED SEIZURE	PG	VPA	CBZ	93.00

	PC	after	NO	YES	UNCONTROLLED SEIZURE	LEV	LTG		123.00
CONFORMATION AL RT	PS	after	NO	YES	UNCONTROLLED SEIZURE	PG	VPA	TPM	53.00
	PS	0.00	NO			PHT	PG		138.00
	PC+SGTC	before	NO			CBZ	TGB		122.00
	PC	before	NO			LEV			36.00
	PC+SGTC	before	NO			LEV	CBZ		14.00
	PC+SGTC	before	YES			PG	PHT		2.00
	PC	after	NO			CBZ	LTG	TGB	36.00
	PS	before	YES			TPM	CBZ		0.00
	PS	before	YES		UNCONTROLLED SEIZURE	CBZ	PG	OTHER	74.00
	PC+SGTC	before	YES	YES	UNCONTROLLED SEIZURE	CBZ	PG	TPM	27.00
	PC	before	YES	YES	UNCONTROLLED SEIZURE	PG	TGB	CBZ	21.00
	PC	before	YES	YES	UNCONTROLLED SEIZURE	PG	CBZ	TGB	21.00
	PC	before	YES	YES	UNCONTROLLED SEIZURE	PHT	PG	CBZ	116.00
		before	YES	YES	UNCONTROLLED SEIZURE	CBZ	TGB	TPM	204.00
	PS+SGTC	before	YES	YES	UNCONTROLLED SEIZURE	CBZ	TGB	OXC	180.00
	PS	0.00							
	PC	0.00	YES	YES	UNCONTROLLED SEIZURE	PG	PHT	TGB	116.00
	PC	before	YES	YES	UNCONTROLLED SEIZURE	CBZ	OTHER	PG	383.00
	PC	before	NO	NO		CBZ	OXC	LEV	6.00
CONFORMATION AL RT	PS	before	NO	YES	SIDE EFFECTS	VPA	LEV		96.00
CONFORMATION AL RT	PS	before	NO	YES	SIDE EFFECTS	VPA	LEV		24.00
	PS+SGTC	before	NO	NO		LEV			12.00
	PC+SGTC	before	NO	NO		LEV			11.00
	PC	before	NO	NO		LEV			25.00
	PC	before	NO	NO		LEV			23.00
	PS	before	YES	NO		LEV			32.00
CONFORMATION AL RT	PC+SGTC	before	YES	YES	UNCONTROLLED SEIZURE	CBZ	LEV		38.00
CONFORMATION AL RT	PS	before	NO	NO		LEV	LCM		9.00
CONFORMATION AL RT	PS	before	YES	YES	SIDE EFFECTS+UNCONTROLLEI	CBZ	LEV		76.00
CONFORMATION AL RT	PS	before	NO	NO		LEV			40.00
	PC	before	NO	NO		OXC			46.00
CONFORMATION AL RT	PS+SGTC	before	NO	NO		VPA	LEV	LCM	84.00
CONFORMATION AL RT	PS+SGTC	before	NO	YES	SIDE EFFECTS+UNCONTROLLEI	VPA	LEV	LTG	108.00
CONFORMATION AL RT	PS	before	NO	NO		LEV			25.00
CONFORMATION AL RT	PS	before	YES	YES	SIDE EFFECTS+UNCONTROLLEI	CBZ	VPA	LEV	41.00
CONFORMATION AL RT	PC	before	NO	NO		LEV			76.00
	PS	before	NO	NO		LEV			96.00
CONFORMATION AL RT	PS	before	YES	NO		LEV	LCM		10.00
CONFORMATION AL RT		before	NO	NO		LEV			15.00
	PS+SGTC	before	NO	YES	UNCONTROLLED SEIZURE	VPA	LEV		96.00
CONFORMATION AL RT	PS+SGTC	before	NO	YES	SIDE EFFECTS+UNCONTROLLEI	CBZ	PHT	OXC	72.00

	PS+SGTC	before	NO	NO		LEV			30.00
	PC	before	NO	NO		OXC			36.00
CONFORMATION AL RT	PS+SGTC	after	YES	NO		CBZ			18.00
CONFORMATION AL RT	PS	before	YES	NO		LEV			25.00
CONFORMATION AL RT	PS	after	NO	NO		LEV			15.00
CONFORMATION AL RT	PS+SGTC	before	YES	YES	UNCONTROLLED SEIZURE	OXC	ZNS		23.00
CONFORMATION AL RT	PS+SGTC	before	NO	NO		PG			28.00
CONFORMATION AL RT	PS+SGTC	after	NO	NO		LEV			18.00
CONFORMATION AL RT	PS	after	NO	YES	UNCONTROLLED SEIZURE	CBZ	LEV		28.00
CONFORMATION AL RT	PS+SGTC	after	NO	NO		PG			8.00
CONFORMATION AL RT	PS+SGTC	before	NO	NO		VPA			31.00
CONFORMATION AL RT	PS+SGTC	after	NO	NO		LEV			24.00
CONFORMATION AL RT	PC	after	NO	YES	UNCONTROLLED SEIZURE	OXC	LEV		15.00
CONFORMATION AL RT	PS	after	NO	YES	UNCONTROLLED SEIZURE	CBZ	LEV		78.00
CONFORMATION AL RT	PS	before	YES	NO		LEV			13.00
CONFORMATION AL RT	PS+SGTC	before	YES	NO		PG	VPA		11.00
CONFORMATION AL RT	PS	before	NO	NO		PG			10.00
CONFORMATION AL RT	PS+SGTC	before	NO	YES	UNCONTROLLED SEIZURE	CBZ	LEV		66.00
CONFORMATION AL RT	PS+SGTC	after	NO	NO		VPA			16.00
CONFORMATION AL RT	PS	before	NO	YES	UNCONTROLLED SEIZURE	LEV	OXC		13.00
CONFORMATION AL RT	PS	after	NO	NO		LEV			13.00
CONFORMATION AL RT	PC	after	NO	NO		PG			77.00
CONFORMATION AL RT	PS+SGTC	before	NO	NO		PG			57.00
CONFORMATION AL RT	PS+SGTC	before	YES	YES	UNCONTROLLED SEIZURE	CBZ	VPA	PG	83.00
CONFORMATION AL RT	PS	before	YES	YES	UNCONTROLLED SEIZURE	CBZ	LEV		22.00
CONFORMATION AL RT	PS+SGTC	after	NO	NO		LEV			20.00
CONFORMATION AL RT	PS	after	NO	NO		LEV			36.00
CONFORMATION AL RT	PS+SGTC	after	NO	NO		LEV			38.00
CONFORMATION AL RT	PC	after	NO	NO		CBZ			48.00
CONFORMATION AL RT	PS+SGTC	after	NO	NO		VPA			24.00
CONFORMATION AL RT	PS	before	YES	NO		VPA			13.00
CONFORMATION AL RT	PS+SGTC	after	NO	NO		PG			39.00
CONFORMATION AL RT	PS+SGTC	after	NO	YES	SIDE EFFECTS	PG	LEV		16.00
CONFORMATION AL RT	PS+SGTC	before	NO	YES	SIDE EFFECTS	PG	VPA		14.00
CONFORMATION AL RT	PS+SGTC	after	YES	YES	UNCONTROLLED SEIZURE	PG	LEV	TPM	23.00
CONFORMATION AL RT	PS+SGTC	before	NO	NO		VPA			26.00
WHOLE BRAIN RT	PS+PC	before	YES	YES	SIDE EFFECTS	OXC	LEV	LCM	8.00
	PS	after	NO	YES	UNCONTROLLED SEIZURE	VPA	OTHER		37.00
	PS	before	YES	YES	UNCONTROLLED SEIZURE	VPA	CBZ		423.00
	PS+SGTC	0.00	YES	YES	UNCONTROLLED SEIZURE	OXC	CBZ		38.00
	PC	before	NO	YES	SIDE EFFECTS	VPA	LEV		104.00
	PC+SGTC	before	NO	YES	UNCONTROLLED SEIZURE	PHT	PG	CBZ	92.00

	PS+PC	before	NO	YES	SIDE EFFECTS	PG	PHT	LEV	87.00
	PS+SGTC	before	YES	YES	UNCONTROLLED SEIZURE	CBZ	LEV		22.00
	PS	before	NO	YES	SIDE EFFECTS	OXC	OTHER	CBZ	75.00
	PS	after	YES	NO		LTG			35.00
	PS	before	NO	YES	UNCONTROLLED SEIZURE	VPA	LEV		159.00
	PS	before	NO	YES	UNCONTROLLED SEIZURE	VPA	CBZ	LCM	99.00
	PS	before	YES	YES	SIDE EFFECTS	CBZ	LEV		39.00
	PS	0.00	NO	YES	SIDE EFFECTS	CBZ	OTHER		47.00
	PS	before	NO	YES	UNCONTROLLED SEIZURE	PHT	CBZ	LTG	51.00
	PS	before	NO	YES	UNCONTROLLED SEIZURE	VPA	CBZ		101.00
	GTC	before	NO	YES	SIDE EFFECTS	PG	OXC		138.00
	PS+SGTC	before	YES	YES	SIDE EFFECTS	PHT	LEV		24.00
WHOLE BRAIN RT	PS+SGTC	before	NO	YES	SIDE EFFECTS	VPA	CBZ		104.00
		0.00		NO		OXC			63.00
WHOLE BRAIN RT	PS+PC	before	YES	YES	UNCONTROLLED SEIZURE	LEV	CBZ	LCM	16.00
	PC+SGTC	before	NO	NO		OXC			82.00
	PS	before	NO	NO		OXC			49.00
	PS	before	NO	NO		CBZ			51.00
WHOLE BRAIN RT	PS	before	YES	YES	UNCONTROLLED SEIZURE	OXC	LEV	LCM	44.00
	PS	before	NO	YES	SIDE EFFECTS	CBZ	OXC		76.00
	PC+SGTC	before	NO	YES	UNCONTROLLED SEIZURE	PG	CBZ		159.00
	PC+SGTC	before	NO	NO		OXC			63.00
	PC+SGTC	before	YES	YES	UNCONTROLLED SEIZURE	VPA	LCM	LEV	26.00
	PC+SGTC	before	YES	YES	UNCONTROLLED SEIZURE	PG	CBZ		375.00
WHOLE BRAIN RT	PS	before	NO	YES	SIDE EFFECTS	VPA	LEV		63.00
	PC+SGTC	before	NO	YES	UNCONTROLLED SEIZURE	VPA	LEV		42.00
	PS+SGTC	before	NO	YES	UNCONTROLLED SEIZURE	OXC	LEV		63.00
	GTC	before	NO	NO		OXC			48.00
	PS	before	NO	NO		OTHER			123.00
	PS	before	YES	YES	UNCONTROLLED SEIZURE	VPA	CBZ		30.00
	PS	before	YES	YES	UNCONTROLLED SEIZURE	CBZ	OTHER	LCM	243.00
	PS	before	YES	YES	UNCONTROLLED SEIZURE	OTHER	OXC	CBZ	108.00
	GTC	after	NO	YES	UNCONTROLLED SEIZURE	LEV	CBZ		16.00
	PS	before	YES	YES	SIDE EFFECTS	LEV	OXC		32.00
	PS	before	NO	NO		OXC			43.00
	PS	before	YES	YES	UNCONTROLLED SEIZURE	PG	OXC	LEV	159.00
	PS	before	NO	NO		CBZ			50.00
	PS+SGTC	before	NO	NO		CBZ			94.00
	PC	before	NO	YES	UNCONTROLLED SEIZURE	PG	OXC	TPM	171.00
	PS+SGTC	before	YES	YES	UNCONTROLLED SEIZURE	PG	OXC	CBZ	28.00
WHOLE BRAIN RT	PS	before	NO	YES	UNCONTROLLED SEIZURE	OXC	LCM		48.00
	PS	before	NO	YES	UNCONTROLLED SEIZURE	TPM	CBZ	LEV	77.00

		PS+SGTC	before	NO	YES	SIDE EFFECTS	VPA	LEV		99.00
		PS+SGTC	before	NO	YES	UNCONTROLLED SEIZURE	VPA	OTHER	OXC	95.00
		PC	before	YES	YES	UNCONTROLLED SEIZURE	OXC	LEV		89.00
WHOLE BRAIN RT		PS+PC	before	NO	YES	UNCONTROLLED SEIZURE	CBZ	LEV		180.00
WHOLE BRAIN RT		PC+SGTC	after	NO	NO		PG			9.00
WHOLE BRAIN RT		PS	before	YES	YES	SIDE EFFECTS	CBZ	PHT	PG	24.00
WHOLE BRAIN RT		PS	before	YES	NO		PHT	LEV		12.00
WHOLE BRAIN RT		PC	before	NO	NO		PG	VPA		5.00
IMRT		PS+SGTC	before	NO	NO		PHT			2.00
IMRT		PS	before	NO	NO		PHT			3.00
WHOLE BRAIN RT		PS+SGTC	before	NO	NO		LTG			4.00
		PS+SGTC	0.00		NO		PG			0.00
		PC	before	NO	NO		OXC			9.00
CONFORMATION AL RT		PS	before	YES	YES	UNCONTROLLED SEIZURE	LEV	PHT		8.00
CONFORMATION AL RT		PC	after	YES	NO		PG	OXC	LEV+TPM	105.00
CONFORMATION AL RT		PS+SGTC	before	YES	NO		VPA			11.00
CONFORMATION AL RT		PS	before	YES	NO		OXC			7.00
CONFORMATION AL RT		PC	before	NO	NO		PG			32.00
CONFORMATION AL RT		PC	before	NO	NO		OXC			4.00
CONFORMATION AL RT		PS+SGTC	before	YES	NO		PHT	OXC		6.00
CONFORMATION AL RT		PC	before	NO	NO		LEV			2.00
IMRT			before	NO	NO		LEV			4.00
CONFORMATION AL RT		PS+PC+SGTC	before	NO	NO		CBZ+PB			96.00
CONFORMATION AL RT		PC+SGTC	before	NO	NO		OXC			57.00
CONFORMATION AL RT			before	NO	NO		PG			36.00
CONFORMATION AL RT		GTC	0.00	NO	NO		PHT			18.00
CONFORMATION AL RT		GTC	after	NO	NO		LEV			23.00
CONFORMATION AL RT	CONVULSIVE	PS	0.00	NO	NO		CBZ			26.00
CONFORMATION AL RT			0.00	NO	NO		LEV			35.00
CONFORMATION AL RT		PC+SGTC	before	NO	NO		LEV			10.00
CONFORMATION AL RT			before	NO	NO		CBZ			16.00
CONFORMATION AL RT		PC	before	NO	NO		LEV			29.00
CONFORMATION AL RT		PC	before	YES	YES	UNCONTROLLED SEIZURE	LEV			12.00
IMRT		PS	after	NO	NO		PHT			34.00
CONFORMATION AL RT		PS	after	YES	NO		OXC			26.00
CONFORMATION AL RT		GTC	before	NO	NO		PG			45.00
CONFORMATION AL RT		GTC	before	NO	NO		CBZ	LEV		13.00
		PC+SGTC	before	NO	NO		LEV			1.00
CONFORMATION AL RT		PC+SGTC	before+after	YES	NO		LEV			28.00
CONFORMATION AL RT		PC+SGTC	before	NO	YES	SIDE EFFECTS	PG	LEV		33.00
CONFORMATION AL RT		GTC	before	NO	NO		LEV			28.00
CONFORMATION AL RT		PS	after	NO	NO		PHT	TPM	LEV	61.00

CONFORMATION AL RT	GTC	before+after	NO	NO		CBZ			6.00
CONFORMATION AL RT	PS+SGTC	before+after	YES	YES	UNCONTROLLED SEIZURE	CBZ+PHT	1000.00		21.00
CONFORMATION AL RT	GTC	after	YES	NO		LEV			6.00
CONFORMATION AL RT	PS	before	NO	NO		OXC			10.00
CONFORMATION AL RT	PC	before	YES	NO		LEV	1000.00		29.00
	PC	before	NO	NO		OXC+PB	OXC		72.00
CONFORMATION AL RT	PS	before	NO	NO		PG			39.00
STEREOTASSICA	PS+SGTC	before	YES	NO		CBZ	OXC	TPM	93.00
CONFORMATION AL RT	GTC	before	NO	NO		LEV			29.00
CONFORMATION AL RT	GTC	before	YES	NO		OXC			50.00
CONFORMATION AL RT	PS	after	YES	NO		LEV	300.00		20.00
CONFORMATION AL RT	GTC	before	YES	YES	UNCONTROLLED SEIZURE	PHT	LEV	VPA	43.00
STEREOTASSICA	PC+SGTC	before	NO	NO		PG			74.00
CONFORMATION AL RT	PC	after	NO	NO		PG			59.00
CONFORMATION AL RT	PS	before	NO	NO		CBZ			65.00
CONFORMATION AL RT	PC	before	NO	NO		PG			51.00
	PC	before	YES	NO		LEV	CBZ	VPA	21.00
CONFORMATION AL RT	PS	before	NO	NO		LEV			25.00
CONFORMATION AL RT		before	NO	NO		LEV			24.00
CONFORMATION AL RT		before	NO	NO		PG			65.00
CONFORMATION AL RT	GTC	after	NO	NO		PG			48.00
CONFORMATION AL RT	PS	before	NO	NO		PHT	OXC		87.00
CONFORMATION AL RT	GTC	before	NO	NO		PHT+PB	PG		12.00
CONFORMATION AL RT		before		NO		CBZ	LEV+CBZ		74.00
CONFORMATION AL RT	PS	before	YES	NO		OXC			7.00
CONFORMATION AL RT		before	YES	NO		PG	OXC+PB	CBZ	67.00
IMRT		after	NO	NO		LEV			18.00
CONFORMATION AL RT	GTC	before	NO	NO		LEV			13.00
CONFORMATION AL RT	PS	before	NO	NO		CBZ			44.00
CONFORMATION AL RT	PC	before	NO	NO		CBZ			18.00
CONFORMATION AL RT	PC+SGTC	before	YES	NO		VPA	LEV+VPA		12.00
CONFORMATION AL RT	GTC	after	NO	NO		OXC			3.00
CONFORMATION AL RT	PC	before	NO	NO		LEV			37.00
CONFORMATION AL RT	PS	before	NO	NO		LEV			24.00
CONFORMATION AL RT	PC+SGTC	before	NO	NO		OXC			58.00
CONFORMATION AL RT	PC	before	NO	NO		LEV	30.00		5.00
CONFORMATION AL RT	PC	before	NO	NO		LEV			14.00
	PS	before	NO	NO		LEV			21.00
CONFORMATION AL RT	PC	before	NO	NO		LEV			5.00
CONFORMATION AL RT	PC	before	NO	NO		LEV			2.00
CONFORMATION AL RT	PS+SGTC	before	NO	NO		LEV			9.00
CONFORMATION AL RT	GTC	before	NO	NO	OTHER	PG			66.00

CONFORMATION AL RT		GTC	after		YES	NO		LEV			3.00
CONFORMATION AL RT		PC	before		NO	NO		LEV			4.00
CONFORMATION AL RT		PS+SGTC	before		NO	NO		LEV			7.00
WHOLE BRAIN RT		PC		0.00	NO	NO		LEV			13.00
		GTC	after		YES	NO		LEV			1.00
CONFORMATION AL RT		GTC	after		YES	YES	UNCONTROLLED SEIZURE	LEV	30.00		4.00
CONFORMATION AL RT		PS	before		NO	NO		LEV			5.00
CONFORMATION AL RT		GTC	before		YES	NO		LEV			7.00
CONFORMATION AL RT		GTC	before		NO	NO		LEV			6.00
CONFORMATION AL RT		PS	before		NO	NO		LEV+PHT			6.00
CONFORMATION AL RT		GTC	before		NO	NO		LEV			7.00
CONFORMATION AL RT	CONVULSIVE	PS	before		YES	YES	UNCONTROLLED SEIZURE	LEV			2.00
CONFORMATION AL RT		PC	before		NO	NO		LEV+OXC			9.00
CONFORMATION AL RT		PC		0.00	YES	NO		LEV			4.00
CONFORMATION AL RT		GTC	after		YES	YES	UNCONTROLLED SEIZURE	PG	LEV+VPA		10.00
CONFORMATION AL RT		PC+SGTC	before		NO	NO		LEV			9.00
CONFORMATION AL RT		GTC	before		YES	YES	UNCONTROLLED SEIZURE	LEV	LCM+LEV		8.00
CONFORMATION AL RT		GTC	before		NO	NO		PG			14.00
CONFORMATION AL RT		GTC	before		NO	NO		LEV+VPA			4.00
		GTC	before		NO	NO		LEV			1.00
CONFORMATION AL RT		GTC	before		NO	NO		LEV			2.00
CONFORMATION AL RT		GTC	before		NO	NO		LEV			5.00
CONFORMATION AL RT		PC	before		NO	NO		LEV			4.00
CONFORMATION AL RT		GTC	before		NO	NO		LEV			3.00
CONFORMATION AL RT		PC		0.00	NO	YES	UNCONTROLLED SEIZURE	LTG	PHT	LEV	6.00
		GTC		0.00	NO	NO		CBZ			3.00
CONFORMATION AL RT		PC		0.00	YES	YES	UNCONTROLLED SEIZURE	PHT	OXC		17.00
CONFORMATION AL RT		PC+SGTC		0.00	NO	YES	UNCONTROLLED SEIZURE		OXC	LEV	16.00
CONFORMATION AL RT		PS+SGTC		0.00	NO	NO		LEV			10.00
CONFORMATION AL RT		GTC		0.00	NO	YES	SCARCE COMPLIANCE	PHT			15.00
		GTC		0.00		YES	UNCONTROLLED SEIZURE	PG	VPA	CBZ	
CONFORMATION AL RT		PS		0.00	YES	YES	SIDE EFFECTS	CBZ	OXC		17.00
				0.00	YES	YES	UNCONTROLLED SEIZURE	PG	LEV		12.00
CONFORMATION AL RT		GTC	after		YES	YES	SIDE EFFECTS+SCARCE COMPL	OXC	LEV		20.00
CONFORMATION AL RT		PS+SGTC		0.00	YES	YES	UNCONTROLLED SEIZURE	OXC			6.00
		PC+SGTC	after		NO	YES		PG	OXC		6.00
CONFORMATION AL RT		GTC		0.00	NO	YES	SIDE EFFECTS	CBZ	VPA	LEV	23.00
CONFORMATION AL RT		PC+SGTC		0.00	NO	NO		VPA			
CONFORMATION AL RT		PC		0.00	NO	NO		VPA			
CONFORMATION AL RT		PS		0.00	YES	YES	UNCONTROLLED SEIZURE	LEV			
CONFORMATION AL RT		PS		0.00	YES	YES	UNCONTROLLED SEIZURE	OXC	TPM	LTG	21.00
RADIOSURGERY		PS		0.00	NO	YES	UNCONTROLLED SEIZURE	OXC	PG		2.00

IMRT	PS		0.00	NO	YES	UNCONTROLLED SEIZURE	OXC	LEV		
CONFORMATION AL RT	PC		0.00		NO		VPA			
CONFORMATION AL RT	PC		0.00	NO	NO		VPA			6.00
CONFORMATION AL RT	GTC		0.00	NO	NO		LEV			12.00
CONFORMATION AL RT	PS		0.00	NO	YES	UNCONTROLLED SEIZURE	PG	LEV	OTHER	10.00
CONFORMATION AL RT	GTC		0.00		YES	UNCONTROLLED SEIZURE	OXC	LEV		
CONFORMATION AL RT	PS		0.00	YES	YES	UNCONTROLLED SEIZURE	LEV			4.00
CONFORMATION AL RT	PC+SGTC		0.00		YES	UNCONTROLLED SEIZURE	LEV	OXC		
CONFORMATION AL RT	PS		0.00		YES	SCARCE COMPLIANCE	CBZ	LEV		
CONFORMATION AL RT	PC+SGTC		0.00	YES	YES	UNCONTROLLED SEIZURE	TPM	LEV		5.00
	PS		0.00	YES	NO		LEV			17.00
CONFORMATION AL RT	PC		0.00				LEV			4.00
IMRT	PS+SGTC		0.00	NO	YES	UNCONTROLLED SEIZURE	PG	LEV		3.00
CONFORMATION AL RT	PC+SGTC		0.00		NO		LEV			
WHOLE BRAIN RT	PS		0.00		NO		VPA			15.00
CONFORMATION AL RT	PS		0.00	YES	NO		VPA			4.00
CONFORMATION AL RT	PS		0.00	NO	YES	SIDE EFFECTS	CBZ	LCM	OXC	1.00
CONFORMATION AL RT	PS		0.00	YES	YES	UNCONTROLLED SEIZURE	LEV	OXC		8.00
CONFORMATION AL RT	PS		0.00		NO		LEV			
	GTC		0.00	NO	YES	UNCONTROLLED SEIZURE	CBZ	LEV	TPM	20.00
CONFORMATION AL RT	PC		0.00	NO	YES	UNCONTROLLED SEIZURE	LEV			5.00
	GTC		0.00	NO	NO		CBZ			18.00
CONFORMATION AL RT	PS		0.00	NO	NO		VPA			8.00
CONFORMATION AL RT	PS		0.00	NO	NO		LEV			
CONFORMATION AL RT	GTC		0.00	NO	YES	UNCONTROLLED SEIZURE	CBZ	LEV		12.00
CONFORMATION AL RT	PS		0.00	NO	NO		LEV			1.00
CONFORMATION AL RT	PS+PC		0.00	NO	YES	UNCONTROLLED SEIZURE	LEV	OXC		12.00
CONFORMATION AL RT	GTC		0.00	NO	NO		VPA			13.00
CONFORMATION AL RT	PC		0.00	NO	YES	SIDE EFFECTS	CBZ	PG		
CONFORMATION AL RT	PS		0.00	NO	NO		LEV			17.00
	PS+SGTC		0.00		YES	UNCONTROLLED SEIZURE	LEV			
CONFORMATION AL RT	PS		0.00	NO	NO		OXC			17.00
	PS		0.00		NO		PG			
	PC		0.00	YES	NO		TPM			15.00
CONFORMATION AL RT	PC		0.00	NO	YES	SIDE EFFECTS	PG	LEV		24.00
	GTC		0.00	NO	YES	SIDE EFFECTS	CBZ	LEV		13.00
CONFORMATION AL RT	GTC		0.00	NO	NO		OXC			1.00
	PS+SGTC	after			YES	UNCONTROLLED SEIZURE	LEV	PHT		
	PS	after			NO		LEV			
	PS+SGTC		0.00	NO	YES	UNCONTROLLED SEIZURE		LEV	TPM	23.00
CONFORMATION AL RT	PS	before		NO	NO		OTHER			10.00
	GTC	before		NO	YES	UNCONTROLLED SEIZURE	CBZ	LEV		7.00

	PS	after		NO		LEV			3.00
	GTC	before	NO	YES	SCARCE COMPLIANCE	OTHER	PHT	VPA	3.00
		before		YES	SCARCE COMPLIANCE	PHT	PG		
		before	NO	YES	SIDE EFFECTS	PG	LEV		
	GTC	before		YES	UNCONTROLLED SEIZURE	PG	VPA	CBZ	
RADIOSURGERY	GTC	before	YES	YES	UNCONTROLLED SEIZURE	VPA	LTG	LEV	14.00
CONFORMATION AL RT	GTC	before	NO	YES		PHT			5.00
CONFORMATION AL RT	PS	before	NO	YES	UNCONTROLLED SEIZURE	CBZ	TPM	LEV	25.00
RADIOSURGERY	PS	before	NO	YES	UNCONTROLLED SEIZURE	LEV	OXC		28.00
CONFORMATION AL RT		before	NO	YES	UNCONTROLLED SEIZURE	CBZ	LEV		6.00
	PS	before	NO	YES	UNCONTROLLED SEIZURE	VPA	TPM		5.00
	GTC	before	NO	NO		VPA	LEV		
	GTC	before	NO	NO		LEV	PHT		11.00
CONFORMATION AL RT	GTC	before	NO	NO		LEV			26.00
	GTC	before	NO	NO		VPA			25.00
	GTC	before	NO	NO		LEV			24.00
	PS	before	YES	NO		LEV			120.00
	PS	before	NO	NO		CBZ	PG		20.00
	GTC	before	NO	NO		LEV			9.00
	GTC	before	NO	NO		LEV			12.00
CONFORMATION AL RT	PS+SGTC+GTC	before	YES	YES	UNCONTROLLED SEIZURE	LEV	OXC		85.00
	GTC	before	NO	NO		LEV			18.00
	PS+SGTC	before	NO	NO		LEV			19.00
	PS	before	NO	NO		TPM	PG		2.00
	PS	before	NO	YES	SIDE EFFECTS	PHT	LEV		15.00
	PC+SGTC	before	NO	NO		LTG			22.00
	GTC	before	NO	NO		LEV			6.00
	PS	after	NO	NO		LEV	TPM		1.00
	PS	after	NO	NO		LEV			8.00
	PS	before	NO	NO		LEV			34.00
RADIOSURGERY	GTC	before	NO	NO		VPA			46.00
	PS	before	YES	NO		LEV	OXC		14.00
	GTC	before	NO	NO		LEV	PG		12.00
	PC+SGTC	before	NO	NO		LEV			20.00
	PS	before	NO	NO		OXC			5.00
	GTC	before	NO	NO		PG			21.00
	PS	before	NO	YES	UNCONTROLLED SEIZURE	CBZ	LEV		18.00
	PS	before	NO	NO		LEV			26.00
	PS	before	NO	NO		LEV			11.00
	PS	before	NO	NO		CBZ			
CONFORMATION AL RT	PS	before	NO	NO		LEV			14.00
	PS+SGTC	before		NO		OXC			

	PS+SGTC+GTC	before+after	YES	NO		LEV			20.00
CONFORMATION AL RT	GTC	before	NO	NO		PG	TPM		26.00
CONFORMATION AL RT	PS	before	NO	YES	UNCONTROLLED SEIZURE	PHT	LEV		33.00
CONFORMATION AL RT	PC+SGTC	after	NO	NO		LEV			10.00
WHOLE BRAIN RT	PS	before	NO	NO		LEV			17.00
	PC	before	NO	YES	UNCONTROLLED SEIZURE	VPA	TPM	LEV	139.00
CONFORMATION AL RT	PS+SGTC	before	NO	YES	UNCONTROLLED SEIZURE	TPM	VPA		20.00
	PS	before	NO	YES	SCARCE COMPLIANCE	PG	OXC		84.00
CONFORMATION AL RT	PC	before	NO	YES	UNCONTROLLED SEIZURE	LEV	LCM		17.00
	GTC	before	NO	NO		VPA			7.00
CONFORMATION AL RT	PC	before	NO	NO		LEV			23.00
	PS+SGTC	before	NO	NO		LEV			5.00
CONFORMATION AL RT	PC	before	NO	NO		LEV			96.00
	PC	before	NO	NO		LEV	LCM		36.00
CONFORMATION AL RT	PC	after	NO	NO		LEV			2.00
CONFORMATION AL RT	PS	before	NO	YES	SIDE EFFECTS	PG	LEV		7.00
CONFORMATION AL RT	PS	after	NO	NO		LEV			2.00
CONFORMATION AL RT	GTC	before	NO	YES	UNCONTROLLED SEIZURE	LEV	OXC		96.00
	PC	before	NO	NO		LEV			4.00
CONFORMATION AL RT	GTC	after	NO	NO		OXC			20.00
CONFORMATION AL RT	PS	before	NO	NO		LEV			8.00
CONFORMATION AL RT	GTC	before	NO	NO		OXC			11.00
	PS	before	NO	YES	UNCONTROLLED SEIZURE	PGB	LEV		30.00
	PS	before	NO	NO		CBZ			72.00
CONFORMATION AL RT	GTC	after	NO	NO		LEV	VPA	LEV	7.00
CONFORMATION AL RT	PS	after	NO	NO		LEV			5.00
	GTC	before	NO	NO		LEV			20.00
	PS	before	NO	YES	UNCONTROLLED SEIZURE	PHT	LEV		4.00
	GTC	before	NO	NO		LEV			4.00
CONFORMATION AL RT	GTC	before	NO	NO		LEV			21.00
	PS	before	YES	NO		LEV			25.00
CONFORMATION AL RT	PC	before	NO	NO		LEV			7.00
CONFORMATION AL RT	PS	after	NO	NO		LEV			13.00
	PC	before	NO	YES	UNCONTROLLED SEIZURE	CBZ	LEV		12.00
CONFORMATION AL RT	GTC	before	NO	YES	UNCONTROLLED SEIZURE	LEV	PHT		19.00
CONFORMATION AL RT	GTC	before	NO	YES	UNCONTROLLED SEIZURE	LEV	VPA	PHT	15.00
CONFORMATION AL RT	GTC	before	NO	NO		LEV			4.00
	GTC	before	NO	NO		LEV			7.00
CONFORMATION AL RT	GTC	before	NO	NO		LEV			19.00
CONFORMATION AL RT	PS	after	NO	NO		LEV			13.00
CONFORMATION AL RT	PS	before	NO	NO		LEV			36.00
CONFORMATION AL RT	GTC	before	NO	NO		LEV			13.00

	GTC	before		NO	NO		LEV			4.00
CONFORMATION AL RT	GTC	before		NO	NO		PG			14.00
	PS	before		NO	NO		LEV			3.00
	PC	before		NO	NO		LEV			17.00
	PS	before		NO	NO		CBZ			2.00
RADIOSURGERY	GTC	before		NO	NO		LEV			15.00
WHOLE BRAIN RT	PS	before		NO	YES	UNCONTROLLED SEIZURE	PG	OXC	LEV	120.00
	GTC	before		NO	NO		LEV			15.00
	GTC	before		NO	NO		LEV			1.00
	PS	before		NO	NO		CBZ			10.00
	PC	before		NO	NO		LEV			15.00
	GTC	after		NO	NO		LEV			1.00
CONFORMATION AL RT	PC	before		NO	YES	UNCONTROLLED SEIZURE	CBZ	VPA		17.00
	PC	before		NO	NO		VPA			7.00
CONFORMATION AL RT		before		NO	NO		LEV			2.00
	GTC	before		NO	NO		LEV			16.00
	GTC	before		YES	NO		LEV			15.00
CONFORMATION AL RT	PS	before		NO	NO					15.00
	PC	before		NO	NO		CBZ			6.00
CONFORMATION AL RT	PC	before		NO	NO		LEV			18.00
CONFORMATION AL RT	PS	before		NO	NO		LEV			8.00
CONFORMATION AL RT	PS	before		NO	YES	UNCONTROLLED SEIZURE	VPA	LEV		2.00
CONFORMATION AL RT	GTC	before		NO	NO		OXC			45.00
CONFORMATION AL RT	PC	before		NO	NO		LEV			14.00
CONFORMATION AL RT	PS	before		NO	NO		LEV			19.00
	PS	after		NO	YES	SCARCE COMPLIANCE	PHT	LEV		1.00
	GTC	before		NO	NO		OXC			25.00
CONFORMATION AL RT	PS+SGTC	before		NO	NO		LEV			16.00
CONFORMATION AL RT	PS	before		NO	NO		LEV			6.00
	PS	before		NO	YES	UNCONTROLLED SEIZURE	LEV	PHT		4.00
RADIOSURGERY	GTC	before		NO						14.00
CONFORMATION AL RT	PS	after		NO	YES	UNCONTROLLED SEIZURE	LEV	VPA		1.00
CONFORMATION AL RT	PS	before		NO	NO		LEV			98.00
CONFORMATION AL RT	GTC	before		NO	NO		PHT	LEV	VPA	194.00
CONFORMATION AL RT	GTC	before		NO	YES	SIDE EFFECTS	VPA	LEV		12.00
CONFORMATION AL RT	PS	before		NO	YES	SIDE EFFECTS	VPA	LEV		33.00
	GTC	before		NO	NO		LEV			2.00
RADIOSURGERY	PS	before		NO	NO		LEV			11.00
CONFORMATION AL RT	PS+SGTC		0.00	YES	YES	SIDE EFFECTS+UNCONTROLLEI	VPA	LEV	PHT	2.00
CONFORMATION AL RT	PS		0.00	NO	NO		LEV			13.00
	GTC		0.00		NO		PG			
CONFORMATION AL RT	PS+SGTC	after		YES	YES	UNCONTROLLED SEIZURE	LEV			3.00

CONFORMATION AL RT	PC		0.00		NO		LEV			12.00
CONFORMATION AL RT			0.00	NO	NO		LEV			11.00
CONFORMATION AL RT	GTC		0.00	NO	YES	UNCONTROLLED SEIZURE	LEV			13.00
	PC	after		NO	YES	UNCONTROLLED SEIZURE	OTHER	PHT		1.00
	GTC		0.00		NO		LEV			
CONFORMATION AL RT	GTC		0.00	NO	YES	UNCONTROLLED SEIZURE	PG	LEV		8.00
	PS		0.00		NO		LEV			
CONFORMATION AL RT	GTC		0.00	NO	YES	UNCONTROLLED SEIZURE	LEV	OTHER		4.00
CONFORMATION AL RT	PS	before		NO	YES	UNCONTROLLED SEIZURE	TPM			3.00
	PS+SGTC	before+after		NO	YES	UNCONTROLLED SEIZURE	LEV	VPA		4.00
CONFORMATION AL RT			0.00	NO	YES	SIDE EFFECTS	LEV			19.00
CONFORMATION AL RT	PS		0.00	NO	YES	SIDE EFFECTS	LEV	OXC	VPA	10.00
	PS+SGTC		0.00		NO		LEV			
CONFORMATION AL RT	PC		0.00	NO	NO					20.00
CONFORMATION AL RT	GTC		0.00	NO	NO		LEV			3.00
STEREOTASSICA	PS		0.00		NO					
	PC		0.00	NO	NO		PHT			2.00
CONFORMATION AL RT	PC		0.00	YES	YES	UNCONTROLLED SEIZURE	LEV	VPA		4.00
CONFORMATION AL RT	PS		0.00	NO	NO		LEV			7.00
CONFORMATION AL RT	PS+SGTC		0.00	NO	NO		LEV			18.00
CONFORMATION AL RT	PS	after		NO	YES	UNCONTROLLED SEIZURE	LEV			11.00
CONFORMATION AL RT	PC		0.00	YES	YES	SIDE EFFECTS+UNCONTROLLEI	CBZ	LEV	TPM	21.00
CONFORMATION AL RT	PS		0.00	NO	NO		LEV			3.00
CONFORMATION AL RT	PS		0.00	NO	YES	SIDE EFFECTS	CBZ	LEV	TPM	19.00
	PS		0.00		NO		LEV			
CONFORMATION AL RT	PS+SGTC		0.00	YES	YES	UNCONTROLLED SEIZURE	OXC	OTHER	LEV	22.00
CONFORMATION AL RT	GTC		0.00	NO	NO		LEV			15.00
CONFORMATION AL RT	PC		0.00	NO	NO		LEV			20.00
CONFORMATION AL RT	PC		0.00	NO	NO					23.00
CONFORMATION AL RT	GTC		0.00	NO	YES	SCARCE COMPLIANCE	VPA	OXC	LEV	22.00
CONFORMATION AL RT	PS		0.00	YES	YES	SIDE EFFECTS	PHT	LEV		13.00
	PS+SGTC		0.00		YES	UNCONTROLLED SEIZURE	LEV			
	PC	after			YES	UNCONTROLLED SEIZURE	LEV			
	PS		0.00	NO	NO		LEV			17.00
CONFORMATION AL RT	PC		0.00	NO	NO		LEV			16.00
CONFORMATION AL RT	PS		0.00	YES	YES	UNCONTROLLED SEIZURE	LEV			13.00
	PS		0.00		NO		LEV			
			0.00	NO	NO		LEV			
	PC+SGTC	after		YES			VPA	LEV		4.00
WHOLE BRAIN RT	PS+SGTC	before		YES			OXC	LEV	LCM	12.00
	GTC	before		NO			OXC			24.00
CONFORMATION AL RT	GTC	before		NO			PG			15.00

CONFORMATION AL RT	GTC	before	NO	OXC		13.00
WHOLE BRAIN RT	PS	before	YES	LEV	CBZ	6.00
CONFORMATION AL RT	PS	before	YES	VPA		9.00
CONFORMATION AL RT	PS	after	NO	LEV		12.00
CONFORMATION AL RT	GTC	before	NO	OXC		18.00
	PC	before	NO	LEV		17.00
	PC	before	YES	VPA		17.00
WHOLE BRAIN RT	PS	after	YES	LEV		9.00
CONFORMATION AL RT	PC	before	NO	LEV		13.00
CONFORMATION AL RT	PC+SGTC	before	YES	LEV		8.00
CONFORMATION AL RT	PC	after	YES	OXC		9.00
CONFORMATION AL RT	GTC	after	YES	LEV		9.00
CONFORMATION AL RT	PS	before	NO	VPA		7.00

time_mod_aed	proximity	mod_aed	mod_1_aed		first_aed
0.93	1.00	change			OXC
-10.10	1.00	change	SIDE EFFECTS+UNCONTROLLED SEIZURE		PHT
36.53	1.00	no		0.00	LEV
2.03	1.00	change	SIDE EFFECTS		CBZ
1.03	1.00	change	UNCONTROLLED SEIZURE		OXC
33.47	1.00	no		0.00	LEV
40.57	1.00	no		0.00	LEV
34.47	1.00	no		0.00	OXC
3.07	1.00	change	SIDE EFFECTS		PG
26.43	1.00	no		0.00	LEV
		change			LEV
4.00	1.00	add_on	UNCONTROLLED SEIZURE		OXC
34.57		no		0.00	LEV
2.03		change	SIDE EFFECTS		OXC
29.37		no		0.00	LEV
50.73		no		0.00	OXC
2.03		change	SIDE EFFECTS		PHT
33.47		no		0.00	LEV
3.03	1.00	add_on	UNCONTROLLED SEIZURE		OXC
1.00	1.00	change	UNCONTROLLED SEIZURE		PG
2.03		change	SIDE EFFECTS		CBZ
24.37		no		0.00	OXC
2.03		add_on	UNCONTROLLED SEIZURE		CBZ
13.20		no		0.00	LEV
33.57		no		0.00	OXC
14.20	1.00	no		0.00	CBZ
31.53		no		0.00	LEV
19.27		no		0.00	OXC
1.00		change	UNCONTROLLED SEIZURE		PHT
21.33		no		0.00	LEV
	1.00	change	SIDE EFFECTS+UNCONTROLLED SEIZURE		CBZ
19.27		no		0.00	OXC
38.50		no		0.00	OXC
20.27		no		0.00	OXC
2.03		change	SIDE EFFECTS		PHT
25.37		no		0.00	CBZ
36.53		no		0.00	PG
2.03		change	SIDE EFFECTS		PG
2.03	1.00	change	UNCONTROLLED SEIZURE		PG

3.07	1.00	change	UNCONTROLLED SEIZURE		VPA
2.03	1.00	change	UNCONTROLLED SEIZURE		PG
11.17	1.00	add_on	UNCONTROLLED SEIZURE		CBZ
1.47	1.00	change	UNCONTROLLED SEIZURE		
11.17	1.00	add_on	UNCONTROLLED SEIZURE		
	1.00				OXC
4.07	1.00	change	SIDE EFFECTS		PG
	1.00				OXC
	1.00				
	1.00				CBZ
	1.00				
12.63	1.00	change	UNCONTROLLED SEIZURE		CBZ
1.00	2.00	change	SIDE EFFECTS		LEV
44.63	2.00	add_on	UNCONTROLLED SEIZURE		PG
42.57	2.00	no		0.00	VPA
0.00	2.00	change	UNCONTROLLED SEIZURE		VPA
80.40	2.00	no		0.00	VPA
38.63	2.00	no		0.00	VPA
1.03	2.00	change	UNCONTROLLED SEIZURE		VPA
0.00	2.00	add_on	UNCONTROLLED SEIZURE		VPA
55.77	1.00	change	SE+OTHER		PHT
176.03	1.00	add_on	OTHER		PG
13.77	1.00	no		0.00	OXC
1.67	1.00	change	OTHER		VPA
31.00	1.00	change	OTHER		
31.80	1.00	no		0.00	LEV
35.67	1.00	no		0.00	LEV
15.27	1.00	add_on	UNCONTROLLED SEIZURE		LEV
29.23	1.00	no		0.00	LEV
26.83	1.00	no		0.00	LEV
6.57	1.00	change	OTHER		OXC
0.47	1.00	change	OTHER		PG
1.13	1.00	change	OTHER		LEV
3.20	1.00	no		0.00	LEV
18.20	1.00	no		0.00	LEV
5.00	1.00	no		0.00	LEV
5.10	1.00	change	OTHER		PHT
	1.00				
11.13	1.00	change	OTHER		CBZ
	1.00				
	1.00				
17.07	1.00	add_on	UNCONTROLLED SEIZURE		LEV

	1.00				
	1.00				
20.13	1.00	add_on	UNCONTROLLED SEIZURE		LEV
15.37	1.00	add_on	UNCONTROLLED SEIZURE		LEV
27.20	1.00	add_on	UNCONTROLLED SEIZURE		LEV
13.27	1.00	add_on	UNCONTROLLED SEIZURE		LEV
-0.97	1.00	change	SIDE EFFECTS		LEV
	1.00				VPA
	1.00				
20.23	1.00	change	OTHER		PG
21.27	1.00	add_on	UNCONTROLLED SEIZURE		LEV
14.37	1.00	add_on	UNCONTROLLED SEIZURE		LEV
4.17	1.00	add_on	UNCONTROLLED SEIZURE		VPA
19.17	1.00	add_on	UNCONTROLLED SEIZURE		LEV
5.80	1.00	no		0.00	CBZ
10.97	1.00	no		0.00	LEV
	1.00				
9.10	1.00	no		0.00	OXC
36.53	1.00	change	UNCONTROLLED SEIZURE		PG
129.53	1.00	no		0.00	CBZ
14.40	1.00	no		0.00	CBZ
72.60	1.00	change	UNCONTROLLED SEIZURE		PG
13.20	1.00	no		0.00	OXC
1.00	1.00	change	UNCONTROLLED SEIZURE		PHT
6.23	1.00	no		0.00	LEV
19.43	1.00	change	OTHER		LEV
1.77	1.00	add_on	UNCONTROLLED SEIZURE		PHT
6.67	1.00	add_on	UNCONTROLLED SEIZURE		CBZ
24.37	2.00	add_on	UNCONTROLLED SEIZURE		LEV
0.00	2.00	change	SIDE EFFECTS		CBZ
11.70	2.00	no		0.00	PG
12.20	2.00	no		0.00	LEV
12.20	2.00	no		0.00	LEV
11.70	2.00	change	UNCONTROLLED SEIZURE		TPM
11.70	2.00	change	UNCONTROLLED SEIZURE		PG
0.00	2.00	add_on	UNCONTROLLED SEIZURE		CBZ
0.00	2.00	add_on	UNCONTROLLED SEIZURE		PG
0.00	2.00	change	SIDE EFFECTS		PG
11.70	2.00	change	UNCONTROLLED SEIZURE		CBZ
1.13	1.00	change	SIDE EFFECTS		OXC
8.87	1.00	add_on	UNCONTROLLED SEIZURE		PG
8.53	1.00	add_on	UNCONTROLLED SEIZURE		PG

5.63	1.00	add_on	SIDE EFFECTS		PG
1.57	1.00	change	SIDE EFFECTS		PG
1.13	1.00	no		0.00	OXC
8.17	1.00	change	UNCONTROLLED SEIZURE		LEV
0.27	1.00	no		0.00	OXC
51.33	1.00	no		0.00	LEV
1.13	1.00	add_on	UNCONTROLLED SEIZURE		PG
63.87	1.00	change	SCARCE COMPLIANCE		LEV
0.90	1.00	no		0.00	OXC
13.63	1.00	no		0.00	PG
6.10	1.00	no		0.00	
-1.13	1.00	change	SIDE EFFECTS		OXC
8.13	1.00	no		0.00	OXC
0.00	1.00	no		0.00	OXC
6.73	1.00	add_on	UNCONTROLLED SEIZURE		OXC
5.70	1.00	change	SIDE EFFECTS		PG
0.23	1.00	change	SIDE EFFECTS		CBZ
17.47	1.00	change	UNCONTROLLED SEIZURE		OXC
-0.10	1.00	add_on	UNCONTROLLED SEIZURE		OXC
4.40	1.00	change	SCARCE COMPLIANCE		OXC
15.80	1.00	change	UNCONTROLLED SEIZURE		OXC
15.67	1.00	add_on	UNCONTROLLED SEIZURE		OXC
73.03	1.00	change	UNCONTROLLED SEIZURE		OXC
10.90	1.00	add_on	UNCONTROLLED SEIZURE		VPA
239.10	1.00	add_on	UNCONTROLLED SEIZURE		PG
41.53	1.00	no		0.00	OXC
15.67	1.00	add_on	UNCONTROLLED SEIZURE		LEV
68.57	1.00	add_on	SCARCE COMPLIANCE		LEV
1.53	1.00	change	SIDE EFFECTS		LEV
17.20	1.00	add_on	UNCONTROLLED SEIZURE		PG
2.87	1.00	no		0.00	LEV
2.33	1.00	change	SIDE EFFECTS		PG
3.37	1.00	change	SIDE EFFECTS		CBZ
0.17	1.00	add_on	UNCONTROLLED SEIZURE		PG
0.00	1.00	no		0.00	LEV
18.53	1.00	add_on	UNCONTROLLED SEIZURE		OXC
30.37	1.00	add_on	UNCONTROLLED SEIZURE		PG
0.00	1.00	change	SIDE EFFECTS		OXC
6.60	1.00	add_on	UNCONTROLLED SEIZURE		PG
0.00	1.00	change	SIDE EFFECTS		LEV
96.50	1.00	change	SCARCE COMPLIANCE		CBZ
210.10	1.00	no		0.00	CBZ

6.47	1.00	add_on	SIDE EFFECTS		CBZ
14.00	1.00	add_on	SIDE EFFECTS		OXC
8.17	1.00	no		0.00	OXC
3.47	1.00	change	SIDE EFFECTS		PG
13.13	1.00	no		0.00	LEV
1.87	1.00	change	UNCONTROLLED SEIZURE		OXC
42.70	1.00	change	UNCONTROLLED SEIZURE		TPM
0.00	1.00	add_on	UNCONTROLLED SEIZURE		PG
-2.03	1.00	change	SIDE EFFECTS		PG
7.70	1.00	no		0.00	LEV
81.07	1.00	change	SIDE EFFECTS		OXC
5.83	1.00	no		0.00	LEV
11.90	1.00	change	UNCONTROLLED SEIZURE		PG
0.50	1.00	no		0.00	LEV
0.13	1.00	add_on	UNCONTROLLED SEIZURE		PHT
31.90	1.00	add_on	UNCONTROLLED SEIZURE		PG
0.03	1.00	add_on	UNCONTROLLED SEIZURE		PG
8.60	1.00	add_on	UNCONTROLLED SEIZURE		LEV
0.37	1.00	no		0.00	TPM
1.03	1.00	add_on	UNCONTROLLED SEIZURE		PHT
0.20	1.00	no		0.00	LEV
3.53	1.00	no		0.00	CBZ
4.07		change	UNCONTROLLED SEIZURE		TPM
36.03	1.00	add_on	UNCONTROLLED SEIZURE		VPA
1.00	1.00	change	UNCONTROLLED SEIZURE		PG
0.50	1.00	change	SIDE EFFECTS		OXC
4.33	1.00	no		0.00	PHT
		no		0.00	LEV
		no		0.00	VPA
		no		0.00	
		no		0.00	CBZ
		no		0.00	
		no		0.00	
		no		0.00	VPA
2.03	1.00	change	SIDE EFFECTS		PG
7.07	1.00	add_on	UNCONTROLLED SEIZURE		LEV
55.87	1.00	no		0.00	LEV
87.20	1.00	change	SIDE EFFECTS		OXC
0.00	1.00	change	SIDE EFFECTS		PG
5.10	1.00	change	SIDE EFFECTS		PG
0.97	1.00	add_on	SIDE EFFECTS		PG
1.03	1.00	change	SIDE EFFECTS		PG

12.17	1.00	change	SIDE EFFECTS		VPA
122.23	1.00	change	UNCONTROLLED SEIZURE		CBZ
4.07	2.00	add_on	UNCONTROLLED SEIZURE		LEV
5.50	2.00	change	SIDE EFFECTS		CBZ
128.90	1.00	add_on	UNCONTROLLED SEIZURE		LEV
9.17	1.00	change	SIDE EFFECTS		LEV
1.00	1.00	change	SIDE EFFECTS		LEV
121.77	1.00	no		0.00	PG
156.23	2.00	no		0.00	PG
112.63	1.00	no		0.00	TPM
10.60	2.00	add_on	UNCONTROLLED SEIZURE		OXC
14.13	1.00	no		0.00	PG
1.03	1.00	change	SIDE EFFECTS		PG
8.17	1.00	add_on	UNCONTROLLED SEIZURE		OXC
4.07	1.00	change	SIDE EFFECTS		PHT
0.00	1.00	change	SIDE EFFECTS		PG
36.53	1.00	no		0.00	LEV
28.40	1.00	change	UNCONTROLLED SEIZURE		LEV
2.03	1.00	change	SIDE EFFECTS		CBZ
7.07	1.00	no		0.00	LEV
8.10	1.00	add_on	UNCONTROLLED SEIZURE		PG
64.87	1.00	no		0.00	LEV
109.57	2.00	add_on	UNCONTROLLED SEIZURE		LEV
107.53	2.00	add_on	UNCONTROLLED SEIZURE		CBZ
7.70	1.00	change	UNCONTROLLED SEIZURE		PG
1.00	2.00	add_on	UNCONTROLLED SEIZURE		OXC
2.03	1.00	change	SIDE EFFECTS		CBZ
111.60	1.00	change	UNCONTROLLED SEIZURE		TPM
0.00	2.00	add_on	UNCONTROLLED SEIZURE		CBZ
84.73	1.00	add_on	UNCONTROLLED SEIZURE		CBZ
4.10	2.00	add_on	UNCONTROLLED SEIZURE		LEV
4.10	1.00	no		0.00	LEV
0.00	1.00	no		0.00	LEV
11.13	1.00	change	SIDE EFFECTS		LEV
-1.03	1.00	no		0.00	LEV
12.17	2.00	change	UNCONTROLLED SEIZURE		CBZ
0.00	2.00	change	SIDE EFFECTS		LEV
4.10	1.00	add_on	UNCONTROLLED SEIZURE		PG
199.83	1.00	no		0.00	PG
19.30	1.00	change	SIDE EFFECTS		PG
0.00	2.00	add_on	UNCONTROLLED SEIZURE		OXC
133.90	2.00	change	UNCONTROLLED SEIZURE		PHT

0.00	1.00	add_on	UNCONTROLLED SEIZURE		LEV
0.00	1.00	change	SIDE EFFECTS		PG
25.37	1.00	no		0.00	LEV
17.20	1.00	no		0.00	OXC
1.03	1.00	no		0.00	PG
63.47	2.00	no		0.00	TPM
0.00	1.00	change	UNCONTROLLED SEIZURE		PG
0.00	1.00	add_on	UNCONTROLLED SEIZURE		LEV
-6.10	2.00	add_on	UNCONTROLLED SEIZURE		
2.03	2.00	change	UNCONTROLLED SEIZURE		LEV
11.17	2.00	change	UNCONTROLLED SEIZURE		PG
-2.07	1.00	add_on	UNCONTROLLED SEIZURE		OXC
16.23	2.00	add_on	UNCONTROLLED SEIZURE		TPM
-3.60	2.00	add_on	UNCONTROLLED SEIZURE		LEV
13.17	1.00	no		0.00	LEV
5.03	1.00	add_on	UNCONTROLLED SEIZURE		LEV
0.00	1.00	add_on	UNCONTROLLED SEIZURE		LEV
-7.07	1.00	no		0.00	LEV
0.00	2.00	add_on	UNCONTROLLED SEIZURE		LEV
119.23	1.00	change	SIDE EFFECTS		PG
6.03	1.00	change	SIDE EFFECTS		OXC
1.03	1.00	change	SIDE EFFECTS		PHT
	1.00	no		0.00	PG
440.33	1.00	change	SIDE EFFECTS		OXC
0.00	1.00	no		0.00	LEV
2.03	1.00	add_on	UNCONTROLLED SEIZURE		LEV
4.07	1.00	change	SIDE EFFECTS		OXC
	1.00	no		0.00	LEV
94.90	1.00	add_on	UNCONTROLLED SEIZURE		OXC
6.67	2.00	change	SIDE EFFECTS		LEV
1.03	2.00	change	SIDE EFFECTS		LEV
0.00	2.00	change	UNCONTROLLED SEIZURE		PG
1.03	1.00	no		0.00	PG
3.00	1.00	no		0.00	PG
4.07	1.00	add_on	UNCONTROLLED SEIZURE		LEV
3.00	1.00	no		0.00	CBZ
13.20	1.00	no		0.00	TPM
6.13	1.00	no		0.00	OXC
4.07	1.00	no		0.00	PG
3.07	1.00	no		0.00	LEV
21.27	1.00	no		0.00	PG
5.10	1.00	no		0.00	OXC

7.07	1.00	no		0.00	LEV
	1.00				LEV
12.17		change	SIDE EFFECTS		PG
23.87		no		0.00	OXC
23.90	1.00	change	SIDE EFFECTS		PHT
23.90		no		0.00	OXC
11.70		change			PG
43.47	2.00	no		0.00	
49.23		change	SCARCE COMPLIANCE		
					PG
					LEV
10.20		change	SIDE EFFECTS		PG
2.03		add_on	SIDE EFFECTS		PG
					PG
					LEV
4.00		change	SCARCE COMPLIANCE		PG
1.03		change	SIDE EFFECTS		PG
					CBZ
					LEV
					PG
		change	SIDE EFFECTS		PG
0.00		change	OTHER		CBZ
2.03		change	SIDE EFFECTS		PG
0.00		change	SIDE EFFECTS		LEV
					LEV
0.97		change	SIDE EFFECTS		PHT
		add_on	UNCONTROLLED SEIZURE		LEV
					PG
0.00		change			PG
1.03		no		0.00	PG
			SCARCE COMPLIANCE		CBZ
					LEV
402.23		change	SIDE EFFECTS		PG
12.17		change	SIDE EFFECTS		PG
					PG
					LEV
					LEV
					PG
19.33	1.00	change	UNCONTROLLED SEIZURE		CBZ
19.40	1.00	no		0.00	
1.03	1.00	change	UNCONTROLLED SEIZURE		PG

8.17	1.00	change	UNCONTROLLED SEIZURE		LEV
37.57	1.00	add_on	UNCONTROLLED SEIZURE		PG
					PHT
					CBZ
					LEV
					LEV
	1.00				PG
	1.00				CBZ
	1.00	add_on	UNCONTROLLED SEIZURE		TPM
	1.00	add_on	UNCONTROLLED SEIZURE		CBZ
	1.00	no		0.00	CBZ
	1.00	no		0.00	PG
	1.00	no		0.00	PG
	1.00	no		0.00	PHT
	1.00	change	UNCONTROLLED SEIZURE		CBZ
24.33	1.00	add_on	UNCONTROLLED SEIZURE		CBZ
	1.00				
108.00	1.00	add_on	UNCONTROLLED SEIZURE		PG
127.07	1.00	add_on	UNCONTROLLED SEIZURE		CBZ
4.90	1.00	change	SIDE EFFECTS		CBZ
101.47	2.00	change	SIDE EFFECTS		VPA
1.03	1.00	change	SIDE EFFECTS		VPA
13.73	2.00	no		0.00	LEV
20.03		no		0.00	LEV
32.73		no		0.00	LEV
24.40		no		0.00	LEV
35.07		no		0.00	LEV
3.07	1.00	add_on	UNCONTROLLED SEIZURE		CBZ
1.03	1.00	change	SCARCE COMPLIANCE		LEV
14.77	1.00	change	SIDE EFFECTS		CBZ
39.27	1.00	no		0.00	LEV
41.93		no		0.00	OXC
47.67	1.00	add_on	UNCONTROLLED SEIZURE		VPA
24.37	2.00	add_on	UNCONTROLLED SEIZURE		VPA
20.53	1.00	no		0.00	LEV
7.07	2.00	change	SIDE EFFECTS		CBZ
74.10	1.00	no		0.00	LEV
3.93		no		0.00	LEV
1.03	2.00	add_on	UNCONTROLLED SEIZURE		LEV
7.77	1.00	no		0.00	LEV
26.87		add_on	UNCONTROLLED SEIZURE		VPA
6.10	2.00	add_on	SIDE EFFECTS+UNCONTROLLED SEIZURE		CBZ

16.03		no		0.00	LEV
3.90		no		0.00	OXC
14.20	1.00	no		0.00	CBZ
18.30	1.00	no		0.00	LEV
11.20	1.00	no		0.00	LEV
17.20	1.00	change	UNCONTROLLED SEIZURE		OXC
25.37	1.00	no		0.00	PG
12.17	1.00	no		0.00	LEV
11.20	1.00	change	UNCONTROLLED SEIZURE		CBZ
4.00	1.00	no		0.00	PG
18.27	1.00	no		0.00	VPA
24.37	1.00	no		0.00	LEV
8.07	1.00	add_on	UNCONTROLLED SEIZURE		OXC
31.50	1.00	add_on	UNCONTROLLED SEIZURE		CBZ
9.10	1.00	no		0.00	LEV
1.03	1.00	add_on	UNCONTROLLED SEIZURE		PG
10.20	1.00	no		0.00	PG
20.27	1.00	change	UNCONTROLLED SEIZURE		CBZ
11.20	1.00	no		0.00	VPA
10.13	1.00	change	UNCONTROLLED SEIZURE		LEV
11.13	1.00	no		0.00	LEV
72.03	1.00	no		0.00	PG
55.80	1.00	no		0.00	PG
8.10	1.00	add_on	UNCONTROLLED SEIZURE		CBZ
10.13	1.00	add_on	UNCONTROLLED SEIZURE		CBZ
26.43	1.00	no		0.00	LEV
32.40	1.00	no		0.00	LEV
22.37	1.00	no		0.00	LEV
45.63	1.00	no		0.00	CBZ
23.40	1.00	no		0.00	VPA
8.13	1.00	no		0.00	VPA
39.60	1.00	no		0.00	PG
14.17	1.00	add_on	SIDE EFFECTS		PG
5.10	1.00	add_on	SIDE EFFECTS		PG
5.10	1.00	add_on	UNCONTROLLED SEIZURE		PG
15.23	1.00	no		0.00	VPA
3.07	1.00	change	SIDE EFFECTS		OXC
97.40	1.00	no		0.00	VPA
20.27	1.00	add_on	UNCONTROLLED SEIZURE		VPA
13.20	1.00	change	UNCONTROLLED SEIZURE		OXC
8.17	1.00	change	SIDE EFFECTS		VPA
	1.00	change	UNCONTROLLED SEIZURE		PHT

143.60	1.00	add_on	UNCONTROLLED SEIZURE		PG
93.87	1.00	add_on	UNCONTROLLED SEIZURE		CBZ
	1.00	change	SIDE EFFECTS		OXC
182.63	1.00	no		0.00	
89.83	1.00	add_on	UNCONTROLLED SEIZURE		VPA
77.67	1.00	add_on	UNCONTROLLED SEIZURE		VPA
0.00	1.00	change	SIDE EFFECTS		CBZ
0.93	1.00	change	SIDE EFFECTS		CBZ
	1.00	change	UNCONTROLLED SEIZURE		PHT
2.03	1.00	change	UNCONTROLLED SEIZURE		VPA
12.17	1.00	change	SIDE EFFECTS		PG
12.20	1.00	add_on	UNCONTROLLED SEIZURE		PHT
50.73	1.00	change	SIDE EFFECTS		VPA
66.90	1.00	no		0.00	OXC
0.00	1.00	add_on	UNCONTROLLED SEIZURE		LEV
91.90	1.00	no		0.00	OXC
32.50	1.00	no		0.00	OXC
61.40	1.00	no		0.00	CBZ
11.17	1.00	add_on	UNCONTROLLED SEIZURE		OXC
11.17	1.00	change	SIDE EFFECTS		CBZ
39.10	1.00	change	SIDE EFFECTS		PG
82.17	1.00	no		0.00	OXC
11.13	1.00	change	UNCONTROLLED SEIZURE		VPA
	1.00	no		0.00	PG
25.33	1.00	change	SIDE EFFECTS		VPA
0.00	1.00	add_on	UNCONTROLLED SEIZURE		VPA
31.43	1.00	add_on	UNCONTROLLED SEIZURE		OXC
26.37	1.00	no		0.00	OXC
34.47	1.00	no		0.00	
15.23	1.00	change	UNCONTROLLED SEIZURE		VPA
	1.00	add_on	UNCONTROLLED SEIZURE		CBZ
15.23	1.00	change	UNCONTROLLED SEIZURE		
121.30	1.00	add_on	UNCONTROLLED SEIZURE		LEV
15.23	1.00	no		0.00	LEV
55.77	1.00	no		0.00	OXC
152.20	1.00	change	SIDE EFFECTS		PG
15.77	1.00	change	UNCONTROLLED SEIZURE		CBZ
64.93	1.00	no		0.00	CBZ
79.73	1.00	add_on	UNCONTROLLED SEIZURE		PG
40.13	1.00	change	UNCONTROLLED SEIZURE		PG
19.30	1.00	add_on	UNCONTROLLED SEIZURE		OXC
4.07	1.00	change	UNCONTROLLED SEIZURE		TPM

36.07	1.00	change	SIDE EFFECTS		VPA
9.20	1.00	change	UNCONTROLLED SEIZURE		VPA
22.30	1.00	change	SIDE EFFECTS		OXC
183.10	1.00	add_on	UNCONTROLLED SEIZURE		CBZ
8.90	1.00	no		0.00	PG
24.33	1.00	change	UNCONTROLLED SEIZURE		CBZ
0.00	1.00	change	UNCONTROLLED SEIZURE		PHT
12.20	1.00	change	UNCONTROLLED SEIZURE		PG
3.17	1.00	no		0.00	PHT
7.20	1.00	no		0.00	PHT
8.37	1.00	no		0.00	
24.97	1.00	no		0.00	PG
24.97	1.00	no		0.00	OXC
8.13	1.00	change	UNCONTROLLED SEIZURE		LEV
26.80	1.00	change	UNCONTROLLED SEIZURE		PG
18.23	1.00	no		0.00	VPA
43.13	1.00	no		0.00	OXC
90.73	1.00	no		0.00	PG
118.20	1.00	no		0.00	OXC
1.60	1.00	add_on	UNCONTROLLED SEIZURE		PHT
43.00	1.00	no		0.00	LEV
8.87	1.00	no		0.00	LEV
215.70	1.00	no		0.00	
95.33	1.00	no		0.00	OXC
70.43	1.00	no		0.00	PG
	1.00	add_on	UNCONTROLLED SEIZURE		PHT
58.33	1.00	no		0.00	LEV
26.87	1.00	no		0.00	CBZ
29.97	1.00	no		0.00	LEV
11.53	1.00	no		0.00	LEV
272.50	1.00	no		0.00	CBZ
69.17	1.00	no		0.00	LEV
14.10	1.00	no		0.00	LEV
37.57	1.00	no		0.00	PHT
57.20	1.00	no		0.00	OXC
17.43	1.00	no		0.00	PG
178.73	1.00	no		0.00	CBZ
1.53	1.00	no		0.00	LEV
61.33	1.00	no		0.00	LEV
16.57	1.00	change	SIDE EFFECTS		PG
30.73	1.00	no		0.00	LEV
1.00	1.00	change			PHT

7.50	1.00	no		0.00	CBZ
55.30	1.00	change	UNCONTROLLED SEIZURE		
8.43	1.00	no		0.00	LEV
10.20	1.00	no		0.00	OXC
4.50	1.00	add_on	UNCONTROLLED SEIZURE		LEV
26.77	1.00	change			
75.37	1.00	no		0.00	PG
25.40	1.00	change	UNCONTROLLED SEIZURE		CBZ
31.23	1.00	no		0.00	LEV
74.63	1.00	no		0.00	OXC
6.30	1.00	add_on	UNCONTROLLED SEIZURE		LEV
17.63	1.00	add_on	UNCONTROLLED SEIZURE		PHT
27.40	1.00	change			PG
55.77	1.00	no		0.00	PG
191.83	1.00	no		0.00	CBZ
169.37	1.00	no		0.00	PG
11.37	1.00	change	SIDE EFFECTS		LEV
55.37	1.00	no		0.00	LEV
60.60	1.00	no		0.00	LEV
102.87	1.00	change			PG
49.90	1.00	no		0.00	PG
17.03	1.00	change	UNCONTROLLED SEIZURE		PHT
13.67	1.00	no		0.00	
72.90	1.00	no		0.00	CBZ
11.20	1.00	no		0.00	OXC
87.27	1.00	add_on	UNCONTROLLED SEIZURE		PG
51.10	1.00	no		0.00	LEV
14.47	1.00	no		0.00	LEV
66.30	1.00	no		0.00	CBZ
43.57	1.00	no		0.00	CBZ
129.57	1.00	add_on	UNCONTROLLED SEIZURE		VPA
4.57	1.00	no		0.00	OXC
	1.00	no		0.00	LEV
26.13	1.00	no		0.00	LEV
86.37	1.00	no		0.00	OXC
0.90	1.00	add_on	UNCONTROLLED SEIZURE		LEV
15.50	1.00	no		0.00	LEV
55.00	1.00	no		0.00	LEV
4.90	1.00	no		0.00	LEV
	1.00	no		0.00	LEV
9.30	1.00	no		0.00	LEV
20.87	1.00	change			PG

4.40	1.00	no		0.00	LEV
5.10	1.00	no		0.00	LEV
10.37	1.00	no		0.00	LEV
4.43	1.00	add_on	UNCONTROLLED SEIZURE		LEV
2.03	1.00	no		0.00	LEV
4.03	1.00	add_on	UNCONTROLLED SEIZURE		LEV
5.20	1.00	no		0.00	LEV
45.67	1.00	no		0.00	LEV
16.97	1.00	no		0.00	LEV
13.40	1.00	no		0.00	
8.50	1.00	no		0.00	LEV
4.87	1.00	add_on	UNCONTROLLED SEIZURE		LEV
46.17	1.00	no		0.00	
1.63	1.00	no		0.00	LEV
11.30	1.00	change	UNCONTROLLED SEIZURE		PG
19.87	1.00	no		0.00	LEV
4.63	1.00	add_on	UNCONTROLLED SEIZURE		LEV
14.87	1.00	no		0.00	PG
14.97	1.00	no		0.00	
0.93	1.00	no		0.00	LEV
9.30	1.00	no		0.00	LEV
10.00	1.00	no		0.00	LEV
14.57	1.00	no		0.00	LEV
3.53	1.00	no		0.00	LEV
					CBZ
					PHT
					LEV
					PHT
					PG
					CBZ
					PG
					OXC
					OXC
					PG
					CBZ
					VPA
					VPA
					LEV
					OXC
					OXC

OXC
VPA
VPA
LEV
PG
OXC
LEV
LEV
CBZ
TPM
LEV
LEV
PG
LEV
VPA
VPA
CBZ
LEV
LEV
CBZ
LEV
CBZ
VPA
LEV
CBZ
LEV
LEV
VPA
CBZ
LEV
LEV
OXC
PG
TPM
PG
CBZ
OXC
LEV
LEV

CBZ

LEV

PHT
PG
PG
VPA
PHT
CBZ
LEV
CBZ
VPA
VPA
LEV
LEV
VPA
LEV
LEV
CBZ
LEV
LEV
LEV
LEV
LEV
TPM
PHT

LEV
LEV
LEV
LEV
VPA
LEV
LEV
LEV
OXC
PG
CBZ
LEV
LEV
CBZ
LEV
OXC

LEV
PG
PHT
LEV
LEV
VPA
TPM
PG
LEV
VPA
LEV
LEV
LEV
LEV
LEV
PG
LEV
LEV
LEV
OXC
LEV
OXC

CBZ
LEV
LEV
LEV
PHT
LEV
LEV
LEV
LEV
LEV
CBZ
LEV
LEV
LEV
LEV
LEV
LEV
LEV
LEV

LEV
PG
LEV
LEV
CBZ
LEV
PG
LEV
LEV
CBZ
LEV
LEV
CBZ
VPA
LEV
LEV
LEV

CBZ
LEV
LEV
VPA
OXC
LEV
LEV
PHT
OXC
LEV
LEV
LEV

LEV
LEV
PHT
VPA
VPA
LEV
LEV
VPA
LEV
PG
LEV

LEV
LEV
LEV

LEV
PG
LEV
LEV
TPM
LEV
LEV
LEV
LEV

LEV

PHT
LEV
LEV
LEV
LEV
CBZ
LEV
CBZ
LEV
OXC
LEV
LEV

VPA
PHT
LEV
LEV
LEV
LEV
LEV
LEV
LEV
VPA
OXC
OXC
PG

OXC
LEV
VPA
LEV
OXC
LEV
VPA
LEV
LEV
LEV
OXC
LEV
VPA
